# Correction: RTN3 inhibits RIG-I-mediated antiviral responses by impairing TRIM25-mediated K63-linked polyubiquitination

**DOI:** 10.7554/eLife.73737

**Published:** 2021-09-09

**Authors:** Ziwei Yang, Jun Wang, Bailin He, Xiaolin Zhang, Xiaojuan Li, Ersheng Kuang

Yang Z, Wang J, He B, Zhang X, Li X, Kuang E. 2021. RTN3 inhibits RIG-I-mediated antiviral responses by impairing TRIM25-mediated K63-linked polyubiquitination. *eLife* **10**:e68958. doi: 10.7554/eLife.68958.

Published 27, July 2021

We have been recently made aware through PubPeer of mistakes in Figure 2E and Figure 5A.

In Figure 2E, it was pointed out that the α-HA blot band was not a new Western blot band but a replicate of the α-HA blot band that appeared in Figure 2E from the preprint version of the paper, which was before the experiments were repeated and improved for the eLife version of the paper. After careful inspection of original data, we indeed inadvertently used α-HA band from the preprint version when organizing and updating the Figure 2E file for submitting to eLife. A new α-HA blot band has been obtained from original frozen sample and incorporated into the corrected Figure 2E.

The results of quantitative comparison of the indicated protein levels were analyzed by gray intensity scanning of the Western blots, including pIKKαβ/Actin, pTBK1/TBK1, pP65/P65 and pIRF3/IRF3, all which are not related to the α-HA band.

Corrected Figure 2 panel E is shown here:

**Figure 1:**
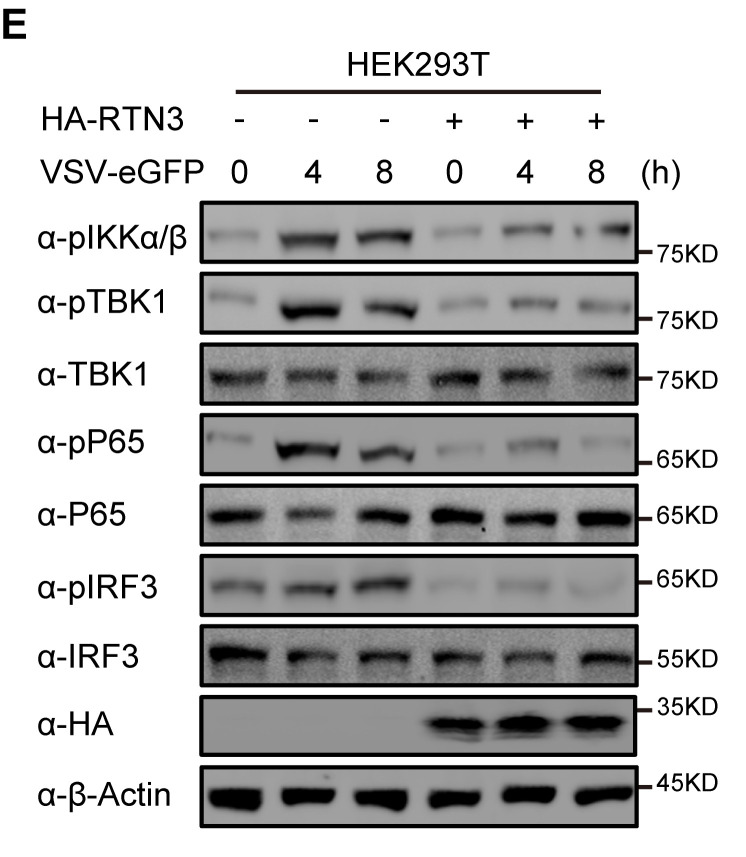


The original Figure 2 panel E is shown here for reference:

**Figure 2:**
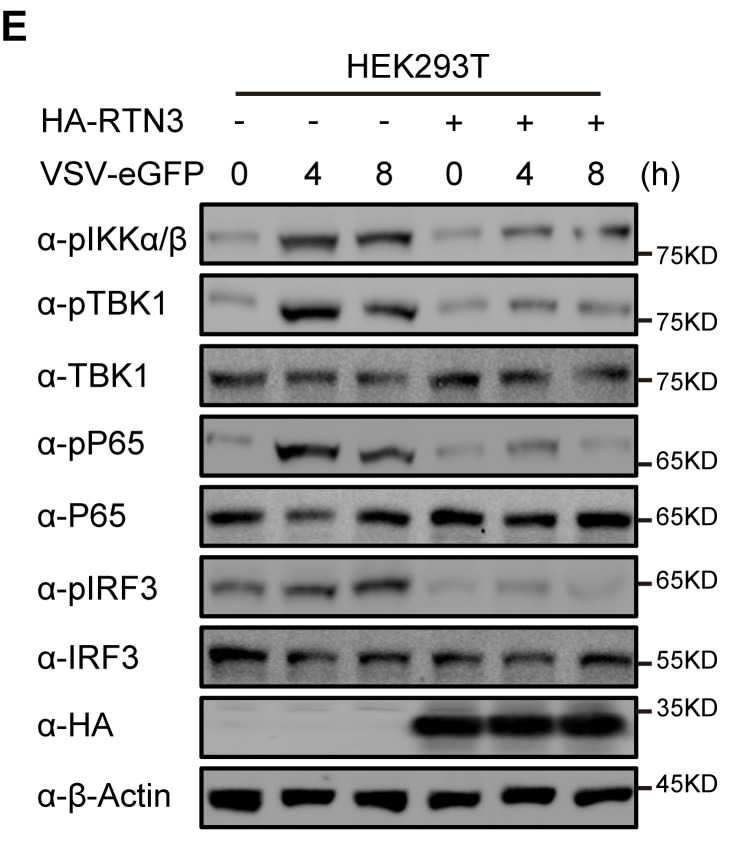


In Figure 5A, α-HA and α-TRIM25 blot bands were pointed out to be identical with α-HA and α-TRIM25 blot bands in Figure 4E of the preprint version of this article. The Figure 5A in eLife paper was the improved version of Figure 4A in the preprint paper, in which the experiments were repeated with the different treatment. α-HA and α-TRIM25 blot bands in the preprint version were inadvertently incorporated into Figure 5A when submitting to eLife. A new α-HA blot band has now been obtained from original frozen sample, and the correct α-TRIM25 blot band has been found from the original images of the repeated experiments, and then Figure 5A has been corrected with these images.

Corrected Figure 5 panel A is shown here:

**Figure 3:**
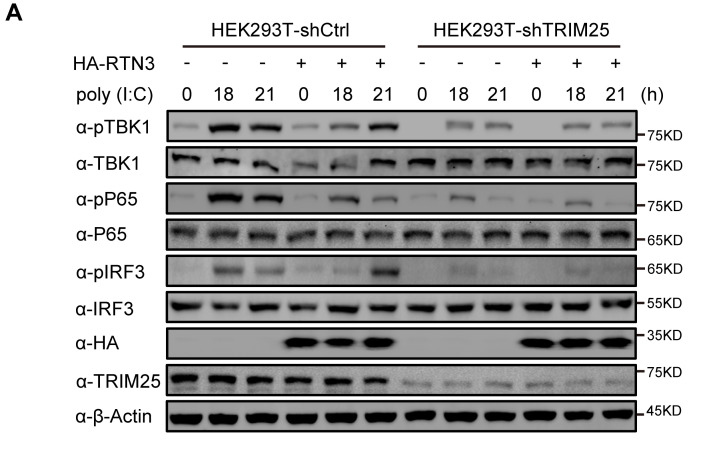


The original Figure 5 panel A is shown here for reference:

**Figure 4:**
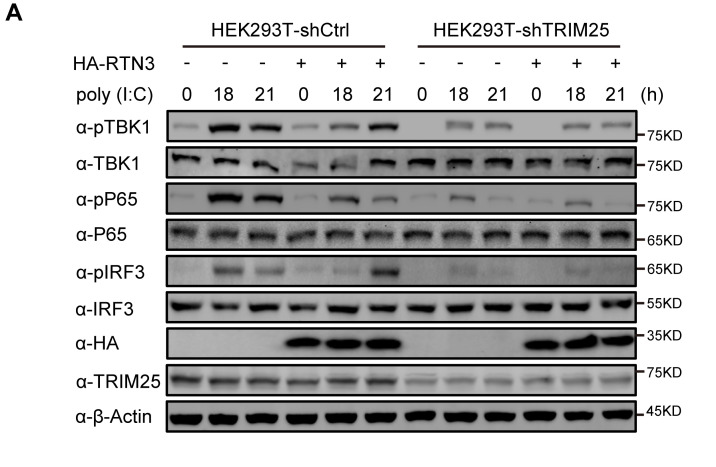


These corrections do not change the conclusions, figure legend and the text of the article. We sincerely apologize for the mistake and any inconvenience that may have caused.

The article has been corrected accordingly.

