# Supplementary figures and images for: RTN3 inhibits RIG-I-mediated antiviral responses by impairing TRIM25-mediated K63-linked polyubiquitination (part 1 of 3)
Source: eLife. 2021 Jul 27;10:e68958. doi: 10.7554/eLife.68958 (PMC8315805; doi:10.7554/eLife.68958)

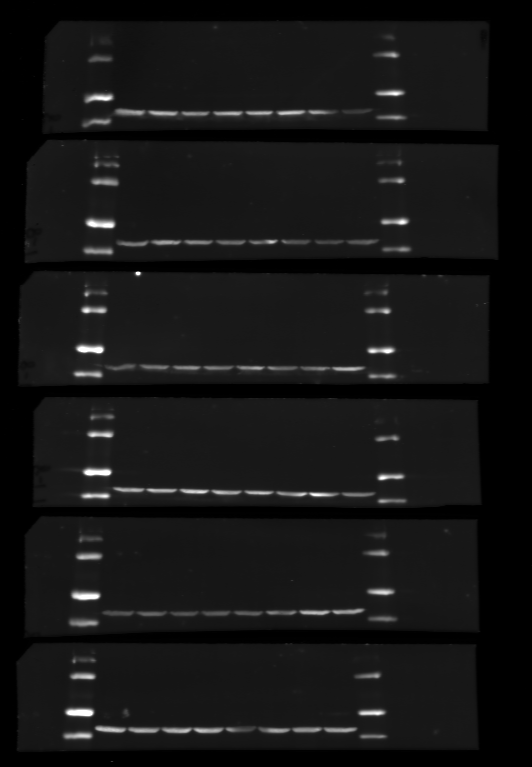

Supplement: Figure 1—source data 1. [file elife-68958-fig1-data1.zip › Figure 1ΓÇôsource data 1/Figure 1 full raw unedited blots files/original_files for D/2020-10-04-165218/700.TIF]

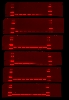

Supplement: Figure 1—source data 1. [file elife-68958-fig1-data1.zip › Figure 1ΓÇôsource data 1/Figure 1 full raw unedited blots files/original_files for D/2020-10-04-165218/2020-10-04-165218_a-Actin_TH.jpg]

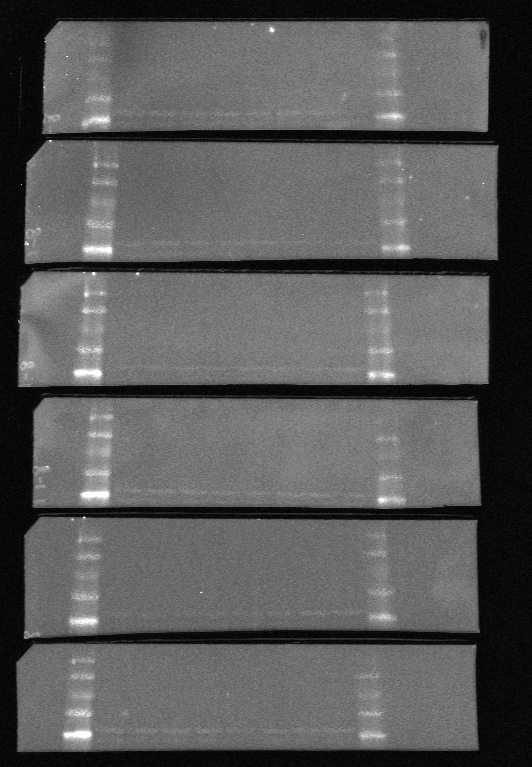

Supplement: Figure 1—source data 1. [file elife-68958-fig1-data1.zip › Figure 1ΓÇôsource data 1/Figure 1 full raw unedited blots files/original_files for D/2020-10-04-165218/800.TIF]

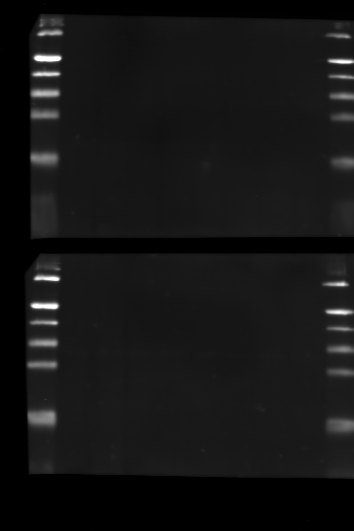

Supplement: Figure 1—source data 1. [file elife-68958-fig1-data1.zip › Figure 1ΓÇôsource data 1/Figure 1 full raw unedited blots files/original_files for D/2020-10-08-122531/700.TIF]

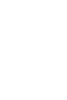

Supplement: Figure 1—source data 1. [file elife-68958-fig1-data1.zip › Figure 1ΓÇôsource data 1/Figure 1 full raw unedited blots files/original_files for D/2020-10-08-122531/2020-10-08-122531_a-RT3_TH.jpg]

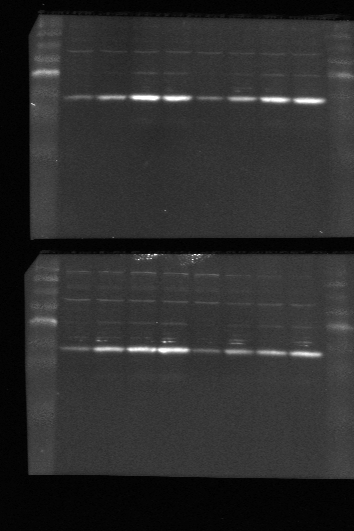

Supplement: Figure 1—source data 1. [file elife-68958-fig1-data1.zip › Figure 1ΓÇôsource data 1/Figure 1 full raw unedited blots files/original_files for D/2020-10-08-122531/800.TIF]

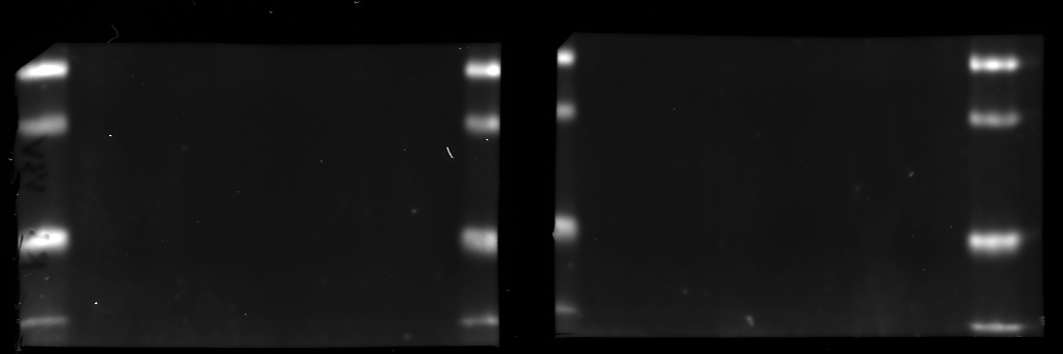

Supplement: Figure 1—source data 1. [file elife-68958-fig1-data1.zip › Figure 1ΓÇôsource data 1/Figure 1 full raw unedited blots files/original_files for F/2021-05-31-170518/700.TIF]

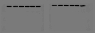

Supplement: Figure 1—source data 1. [file elife-68958-fig1-data1.zip › Figure 1ΓÇôsource data 1/Figure 1 full raw unedited blots files/original_files for F/2021-05-31-170518/2021-05-31-170518_VSV PBMCs RT3_TH.jpg]

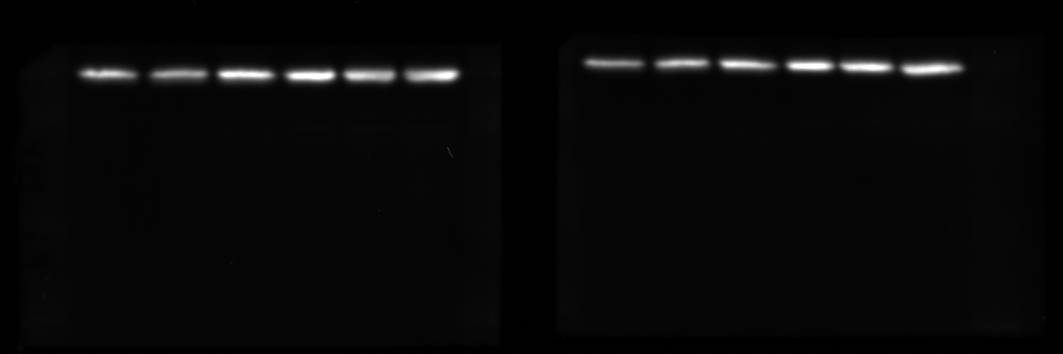

Supplement: Figure 1—source data 1. [file elife-68958-fig1-data1.zip › Figure 1ΓÇôsource data 1/Figure 1 full raw unedited blots files/original_files for F/2021-05-31-170518/800.TIF]

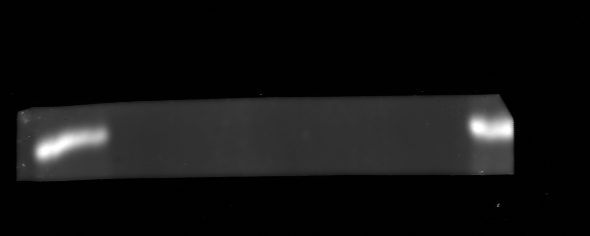

Supplement: Figure 1—source data 1. [file elife-68958-fig1-data1.zip › Figure 1ΓÇôsource data 1/Figure 1 full raw unedited blots files/original_files for F/2021-05-27-203312/700.TIF]

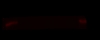

Supplement: Figure 1—source data 1. [file elife-68958-fig1-data1.zip › Figure 1ΓÇôsource data 1/Figure 1 full raw unedited blots files/original_files for F/2021-05-27-203312/2021-05-27-203312_ACTIN_TH.jpg]

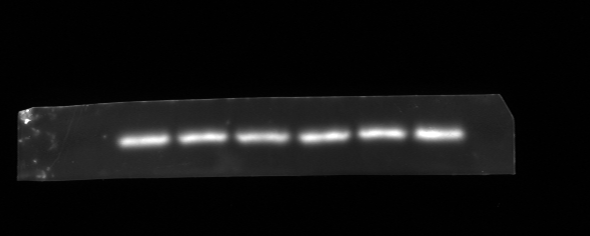

Supplement: Figure 1—source data 1. [file elife-68958-fig1-data1.zip › Figure 1ΓÇôsource data 1/Figure 1 full raw unedited blots files/original_files for F/2021-05-27-203312/800.TIF]

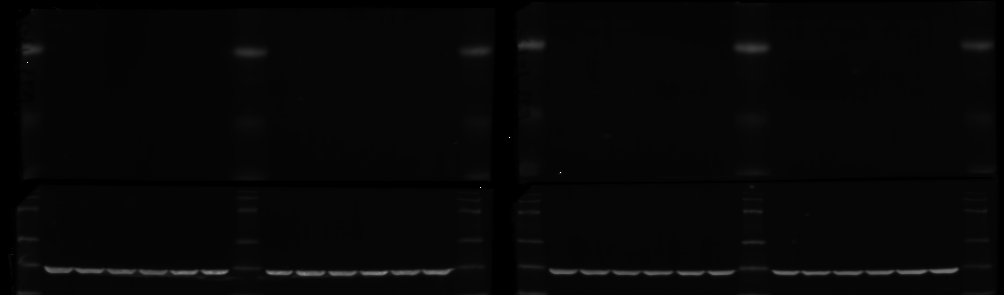

Supplement: Figure 1—source data 1. [file elife-68958-fig1-data1.zip › Figure 1ΓÇôsource data 1/Figure 1 full raw unedited blots files/original_files for A/2020-07-12-220945/700.TIF]

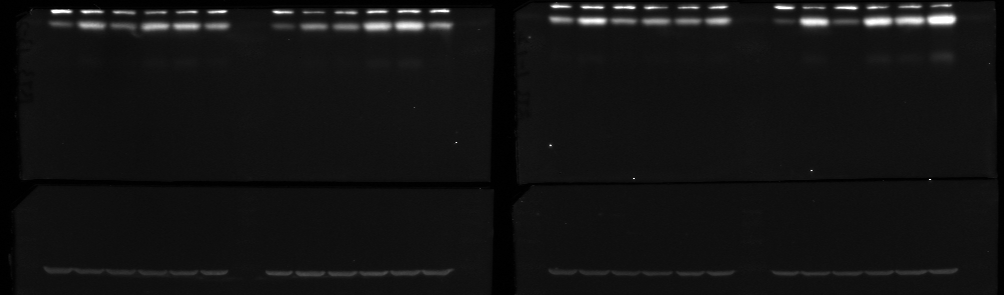

Supplement: Figure 1—source data 1. [file elife-68958-fig1-data1.zip › Figure 1ΓÇôsource data 1/Figure 1 full raw unedited blots files/original_files for A/2020-07-12-220945/800.TIF]

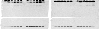

Supplement: Figure 1—source data 1. [file elife-68958-fig1-data1.zip › Figure 1ΓÇôsource data 1/Figure 1 full raw unedited blots files/original_files for A/2020-07-12-220945/2020-07-12-220945_a-RT3 Actin_TH.jpg]

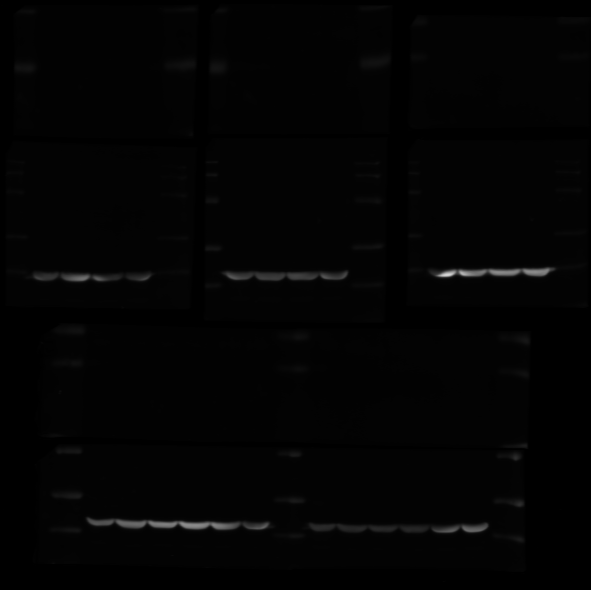

Supplement: Figure 1—source data 1. [file elife-68958-fig1-data1.zip › Figure 1ΓÇôsource data 1/Figure 1 full raw unedited blots files/original_files for A/2020-07-14-202059/700.TIF]

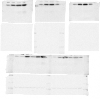

Supplement: Figure 1—source data 1. [file elife-68958-fig1-data1.zip › Figure 1ΓÇôsource data 1/Figure 1 full raw unedited blots files/original_files for A/2020-07-14-202059/2020-07-14-202059_a-RT3 TNFa pIC VSV_TH.jpg]

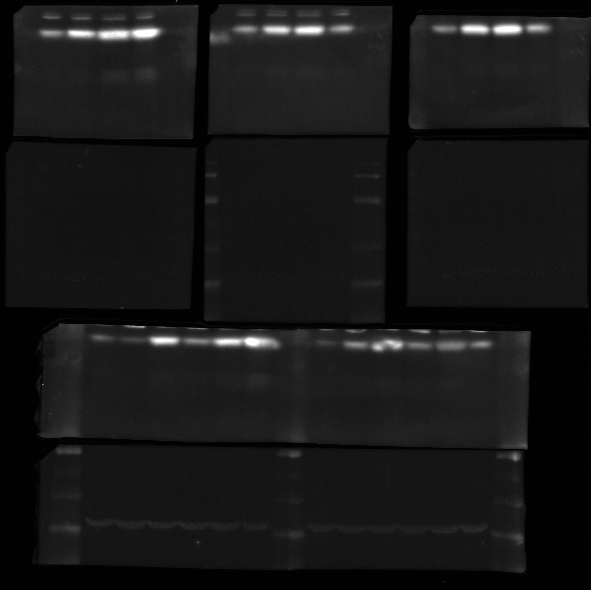

Supplement: Figure 1—source data 1. [file elife-68958-fig1-data1.zip › Figure 1ΓÇôsource data 1/Figure 1 full raw unedited blots files/original_files for A/2020-07-14-202059/800.TIF]

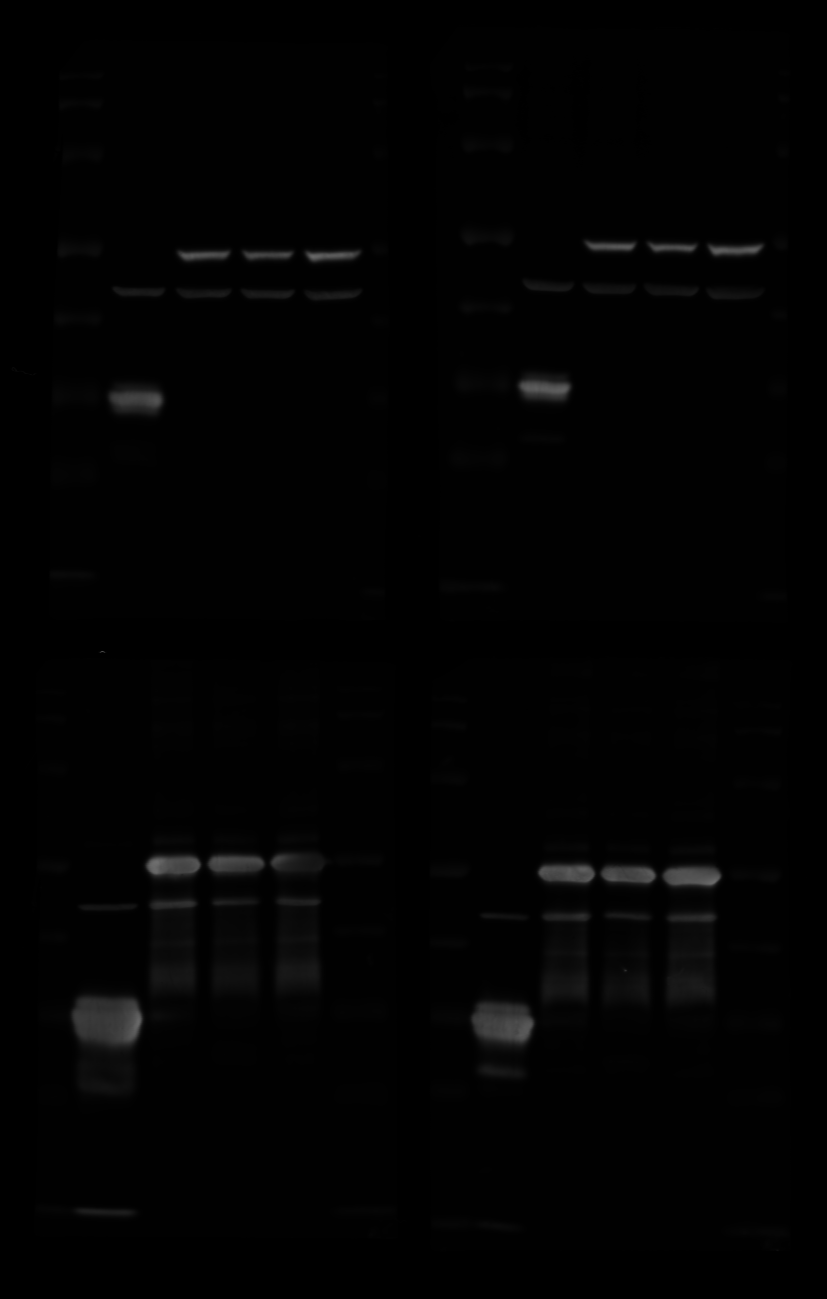

Supplement: Figure 1—figure supplement 1—source data 1. [file elife-68958-fig1-figsupp1-data1.zip › Figure 1-figure supplement 1ΓÇôsource data 1/Figure 1-figure supplement 1 full raw unedited blots files/original_files for H/2021-02-24-132156/700.TIF]

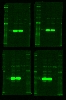

Supplement: Figure 1—figure supplement 1—source data 1. [file elife-68958-fig1-figsupp1-data1.zip › Figure 1-figure supplement 1ΓÇôsource data 1/Figure 1-figure supplement 1 full raw unedited blots files/original_files for H/2021-02-24-132156/2021-02-24-132156_a-Actin_TH.jpg]

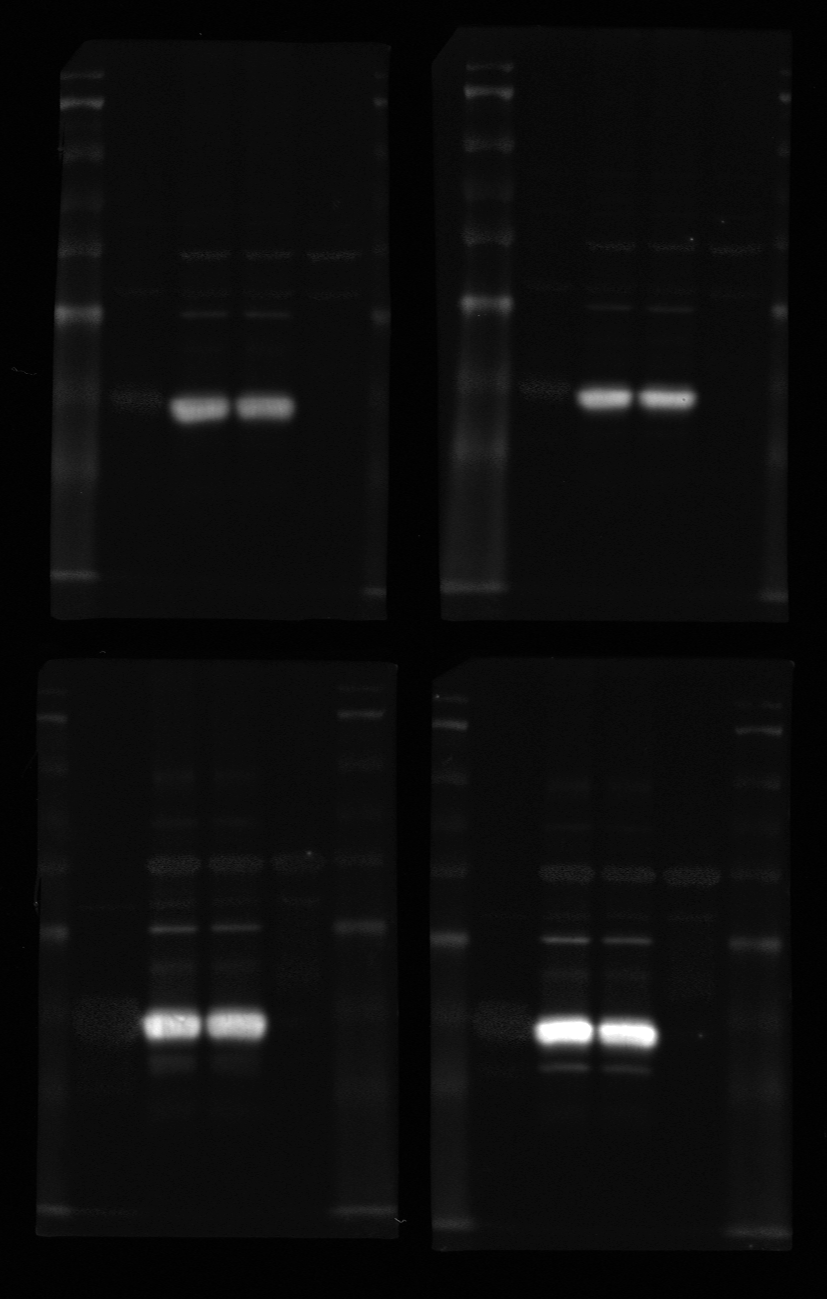

Supplement: Figure 1—figure supplement 1—source data 1. [file elife-68958-fig1-figsupp1-data1.zip › Figure 1-figure supplement 1ΓÇôsource data 1/Figure 1-figure supplement 1 full raw unedited blots files/original_files for H/2021-02-24-132156/800.TIF]

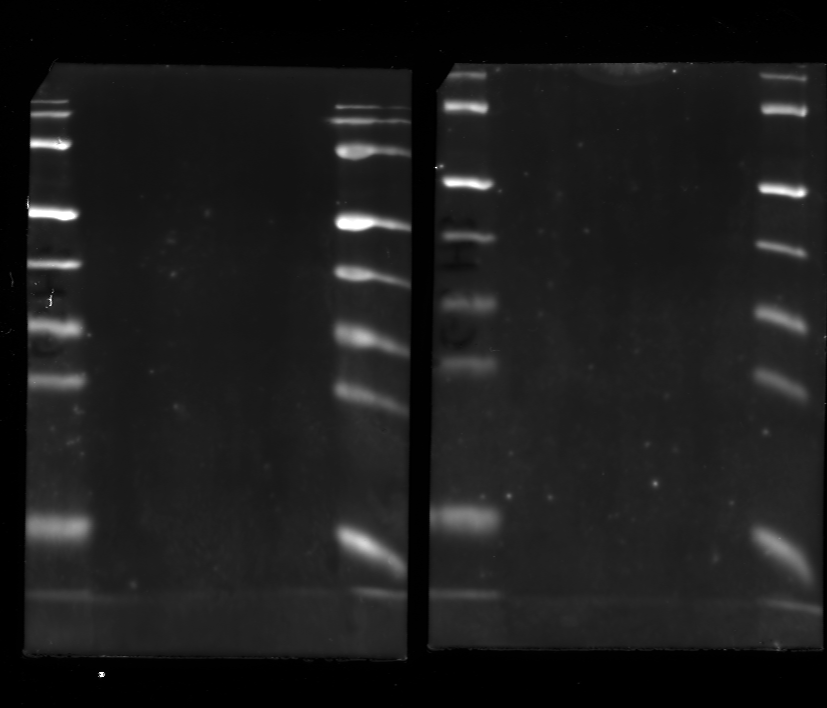

Supplement: Figure 1—figure supplement 1—source data 1. [file elife-68958-fig1-figsupp1-data1.zip › Figure 1-figure supplement 1ΓÇôsource data 1/Figure 1-figure supplement 1 full raw unedited blots files/original_files for H/2021-02-24-133604/700.TIF]

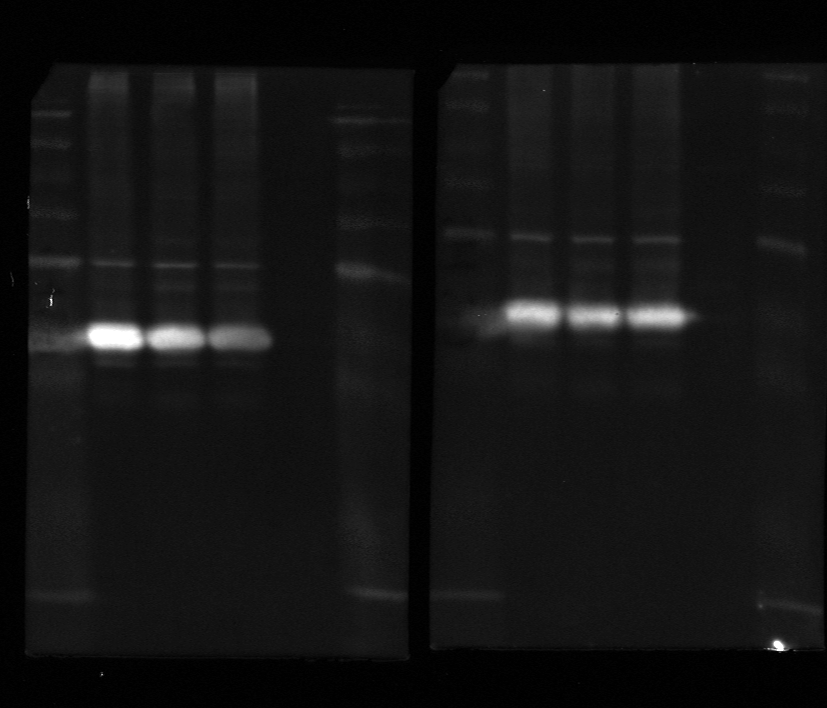

Supplement: Figure 1—figure supplement 1—source data 1. [file elife-68958-fig1-figsupp1-data1.zip › Figure 1-figure supplement 1ΓÇôsource data 1/Figure 1-figure supplement 1 full raw unedited blots files/original_files for H/2021-02-24-133604/800.TIF]

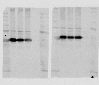

Supplement: Figure 1—figure supplement 1—source data 1. [file elife-68958-fig1-figsupp1-data1.zip › Figure 1-figure supplement 1ΓÇôsource data 1/Figure 1-figure supplement 1 full raw unedited blots files/original_files for H/2021-02-24-133604/2021-02-24-133604_a-HA RT3 input_TH.jpg]

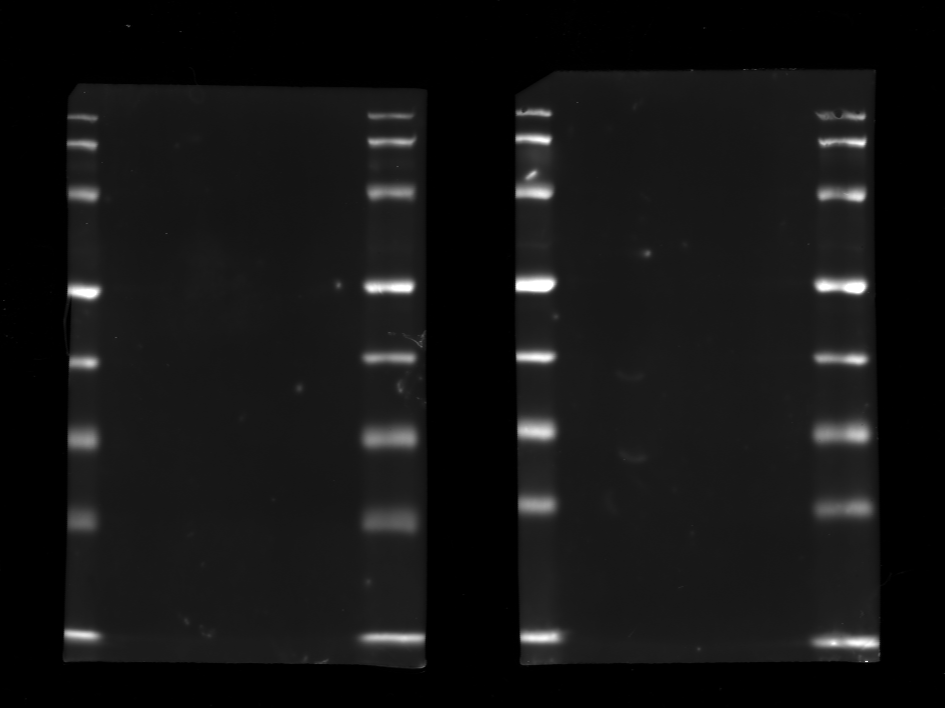

Supplement: Figure 1—figure supplement 1—source data 1. [file elife-68958-fig1-figsupp1-data1.zip › Figure 1-figure supplement 1ΓÇôsource data 1/Figure 1-figure supplement 1 full raw unedited blots files/original_files for H/2021-02-21-193235/700.TIF]

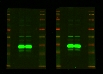

Supplement: Figure 1—figure supplement 1—source data 1. [file elife-68958-fig1-figsupp1-data1.zip › Figure 1-figure supplement 1ΓÇôsource data 1/Figure 1-figure supplement 1 full raw unedited blots files/original_files for H/2021-02-21-193235/2021-02-21-193235_IP a-HA-RT3_TH.jpg]

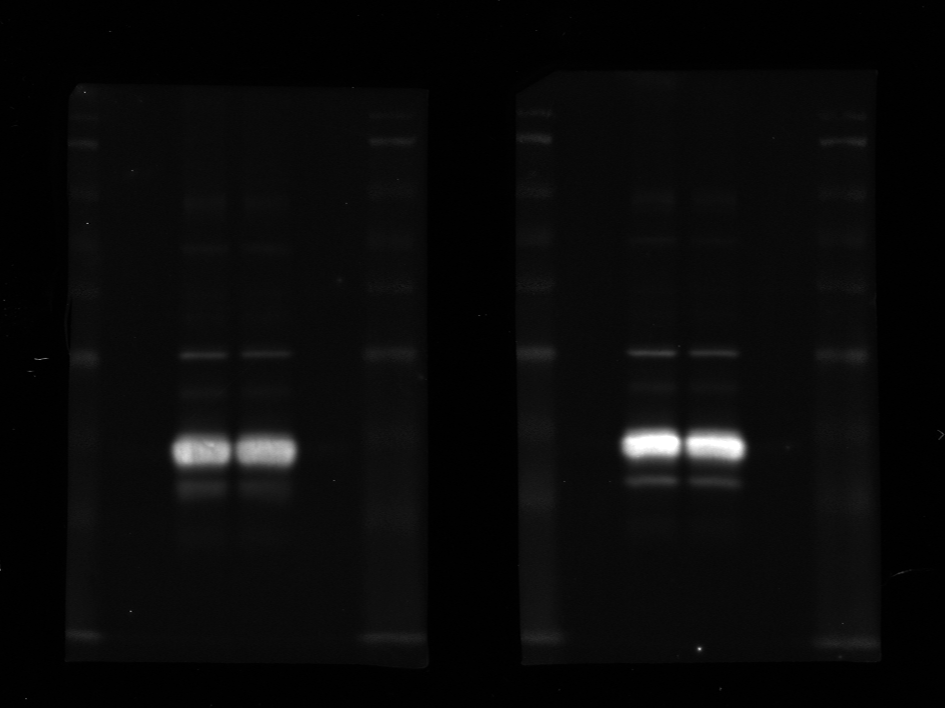

Supplement: Figure 1—figure supplement 1—source data 1. [file elife-68958-fig1-figsupp1-data1.zip › Figure 1-figure supplement 1ΓÇôsource data 1/Figure 1-figure supplement 1 full raw unedited blots files/original_files for H/2021-02-21-193235/800.TIF]

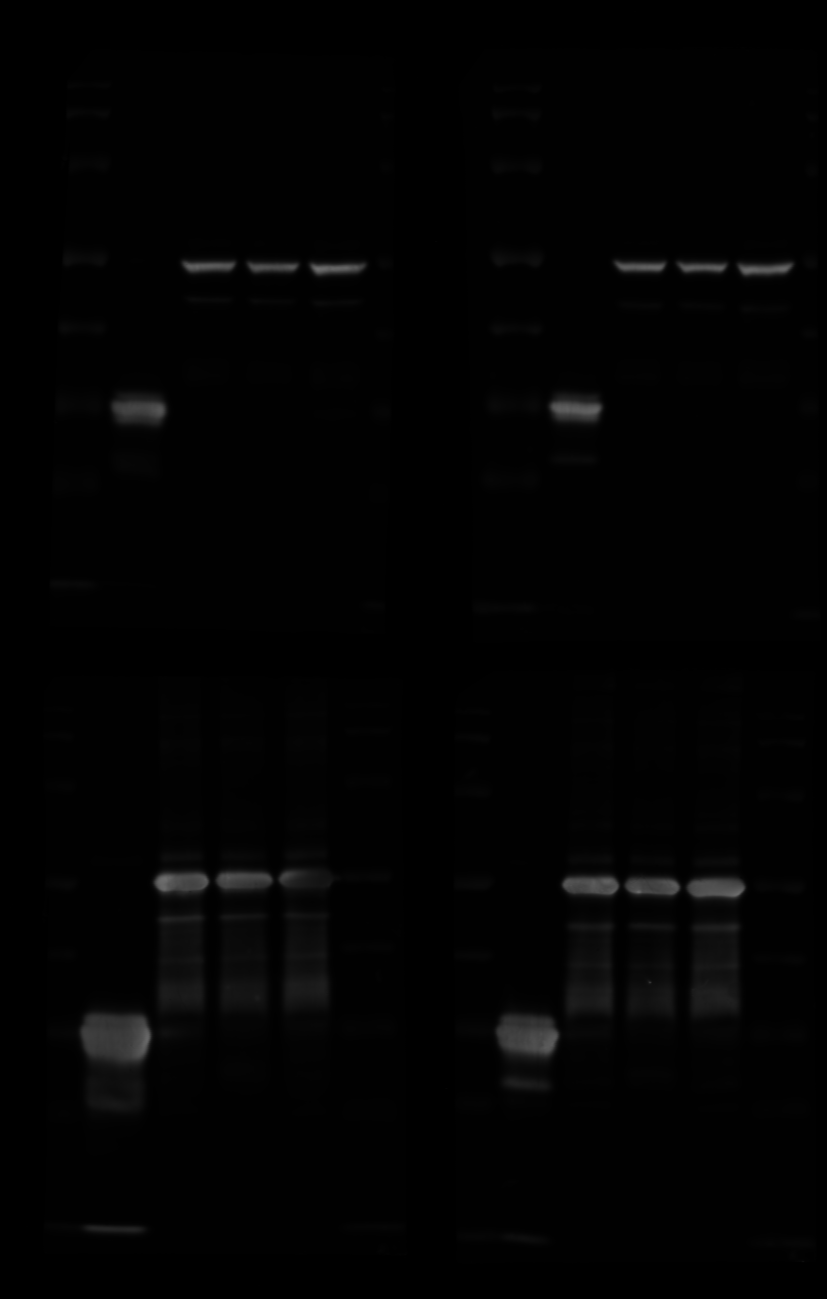

Supplement: Figure 1—figure supplement 1—source data 1. [file elife-68958-fig1-figsupp1-data1.zip › Figure 1-figure supplement 1ΓÇôsource data 1/Figure 1-figure supplement 1 full raw unedited blots files/original_files for H/2021-02-22-210904/700.TIF]

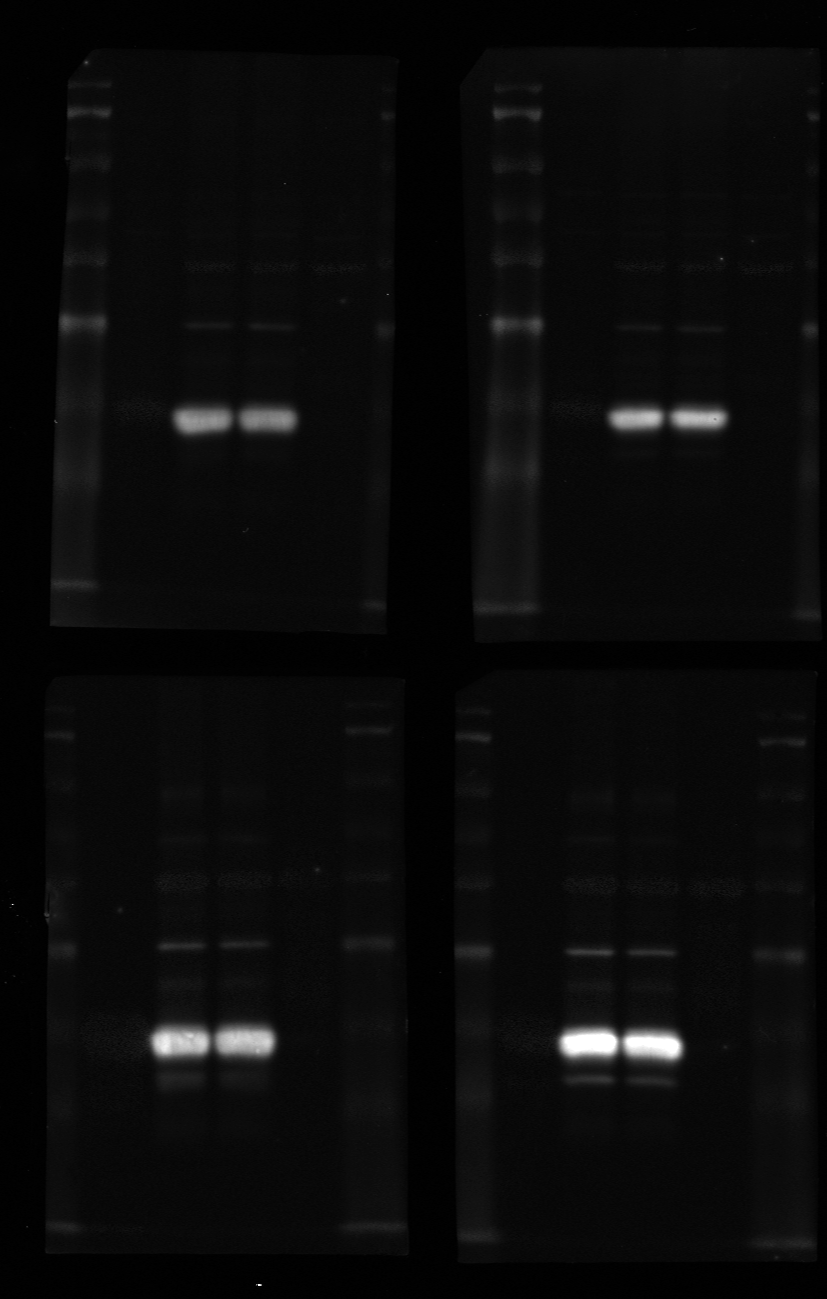

Supplement: Figure 1—figure supplement 1—source data 1. [file elife-68958-fig1-figsupp1-data1.zip › Figure 1-figure supplement 1ΓÇôsource data 1/Figure 1-figure supplement 1 full raw unedited blots files/original_files for H/2021-02-22-210904/800.TIF]

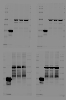

Supplement: Figure 1—figure supplement 1—source data 1. [file elife-68958-fig1-figsupp1-data1.zip › Figure 1-figure supplement 1ΓÇôsource data 1/Figure 1-figure supplement 1 full raw unedited blots files/original_files for H/2021-02-22-210904/2021-02-22-210904_a-GFP RTN3_TH.jpg]

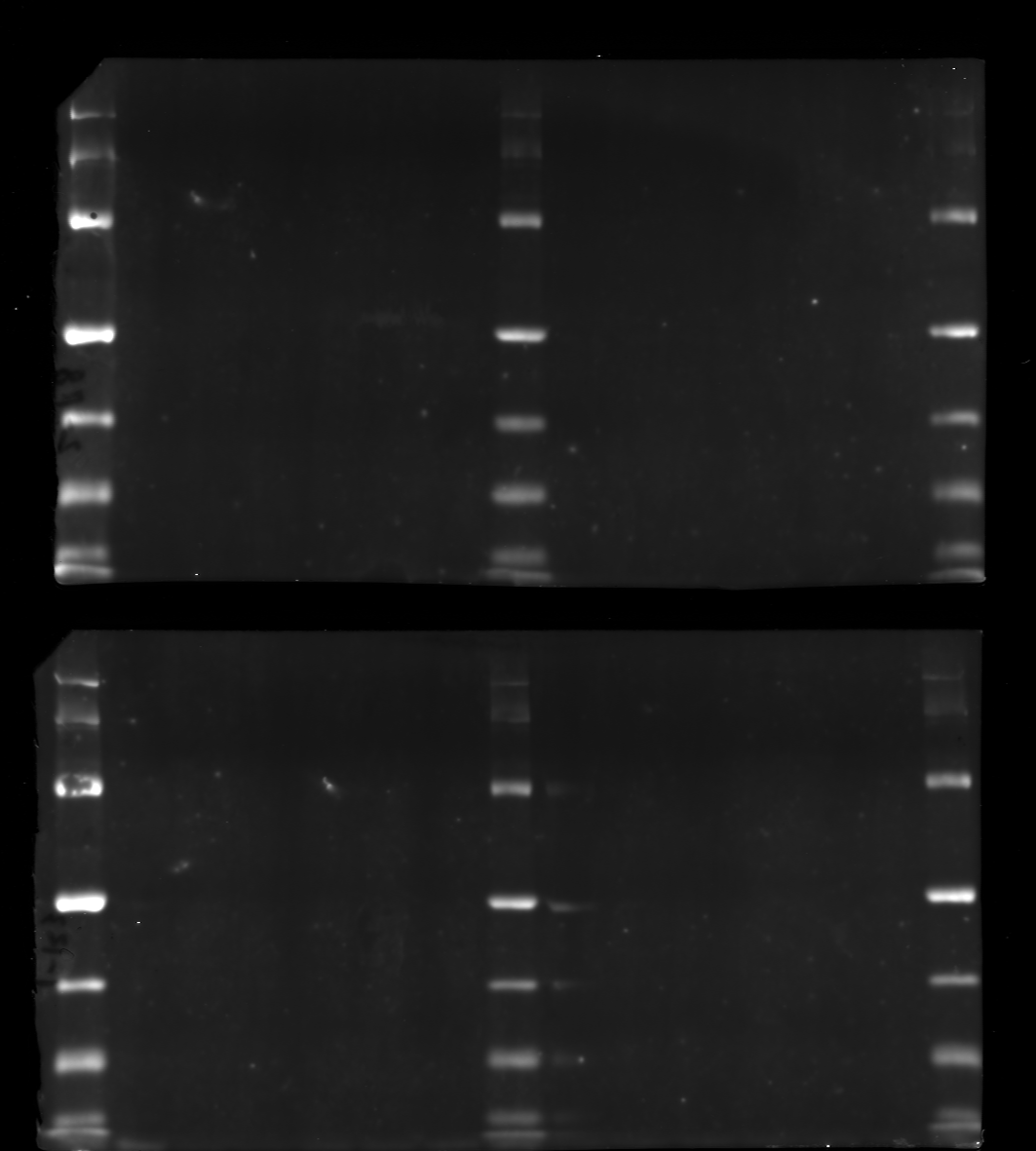

Supplement: Figure 2—source data 1. [file elife-68958-fig2-data1.zip › Figure 2ΓÇôsource data 1/Figure 2 full raw unedited blots files/2021-03-06-211645/700.TIF]

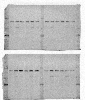

Supplement: Figure 2—source data 1. [file elife-68958-fig2-data1.zip › Figure 2ΓÇôsource data 1/Figure 2 full raw unedited blots files/2021-03-06-211645/2021-03-06-211645_a-pIRF3_TH.jpg]

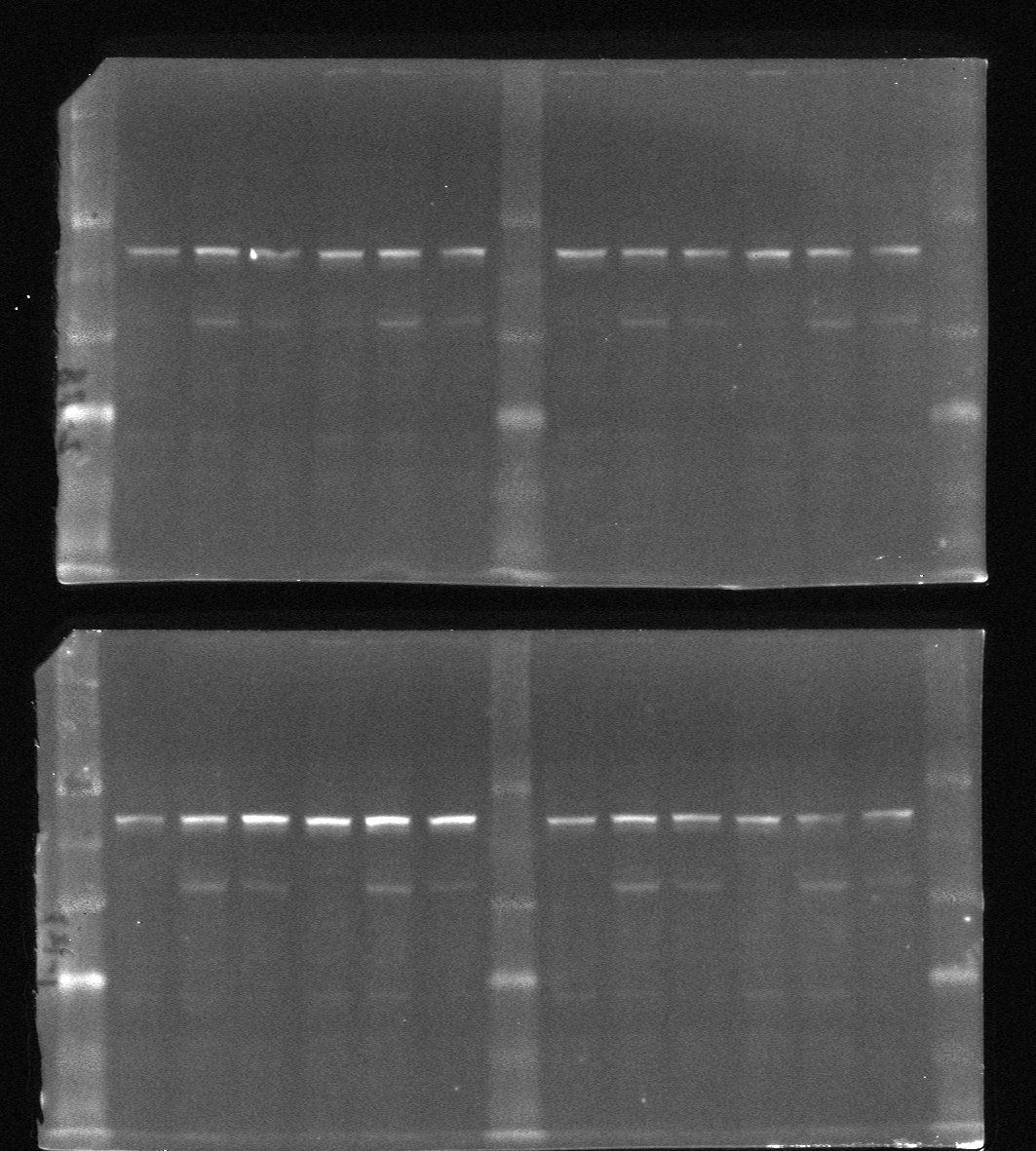

Supplement: Figure 2—source data 1. [file elife-68958-fig2-data1.zip › Figure 2ΓÇôsource data 1/Figure 2 full raw unedited blots files/2021-03-06-211645/800.TIF]

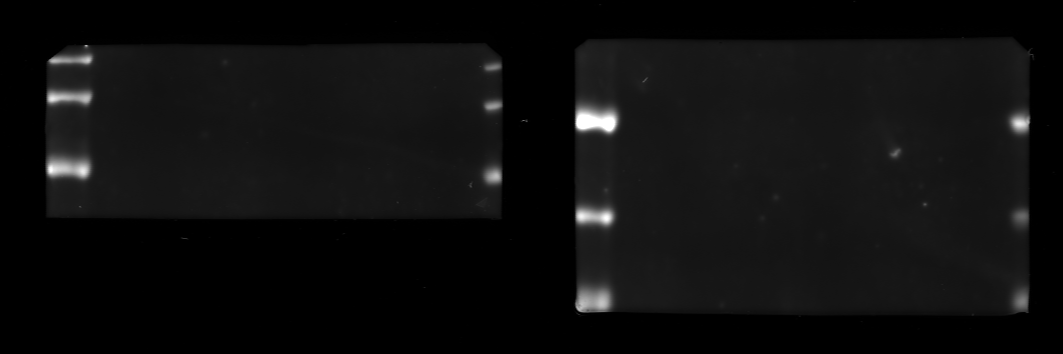

Supplement: Figure 2—source data 1. [file elife-68958-fig2-data1.zip › Figure 2ΓÇôsource data 1/Figure 2 full raw unedited blots files/2021-03-17-192520/700.TIF]

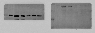

Supplement: Figure 2—source data 1. [file elife-68958-fig2-data1.zip › Figure 2ΓÇôsource data 1/Figure 2 full raw unedited blots files/2021-03-17-192520/2021-03-17-192520_a-pTBK1 pP65_TH.jpg]

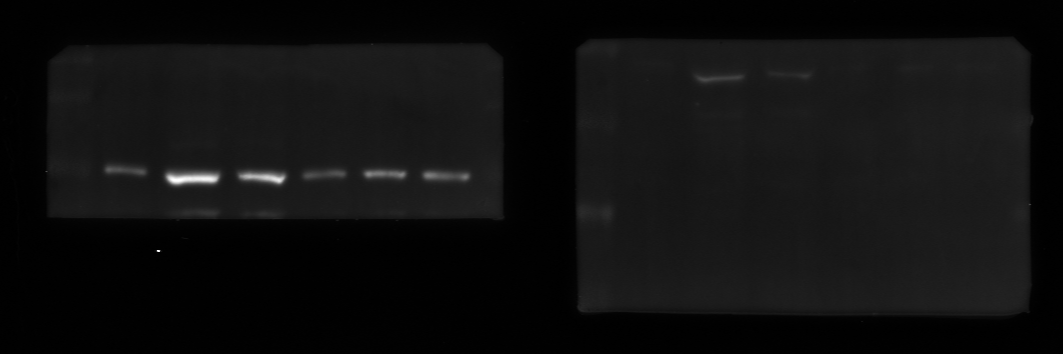

Supplement: Figure 2—source data 1. [file elife-68958-fig2-data1.zip › Figure 2ΓÇôsource data 1/Figure 2 full raw unedited blots files/2021-03-17-192520/800.TIF]

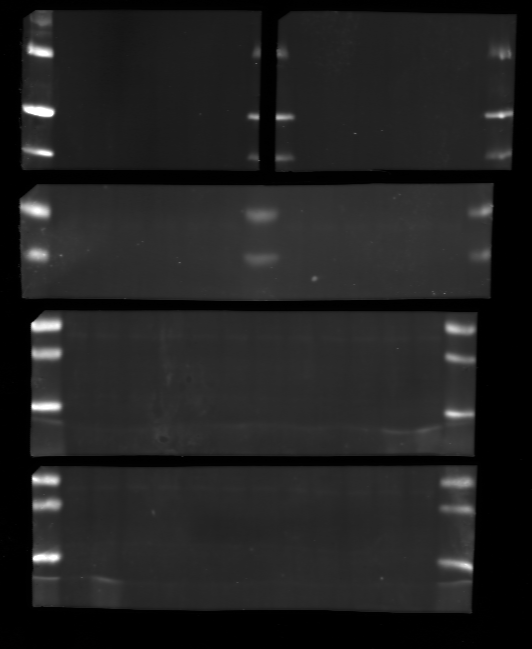

Supplement: Figure 2—source data 1. [file elife-68958-fig2-data1.zip › Figure 2ΓÇôsource data 1/Figure 2 full raw unedited blots files/2020-08-31-213817/700.TIF]

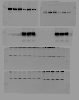

Supplement: Figure 2—source data 1. [file elife-68958-fig2-data1.zip › Figure 2ΓÇôsource data 1/Figure 2 full raw unedited blots files/2020-08-31-213817/2020-08-31-213817_a-pTBK1 pIRF3 HA-RT3_TH.jpg]

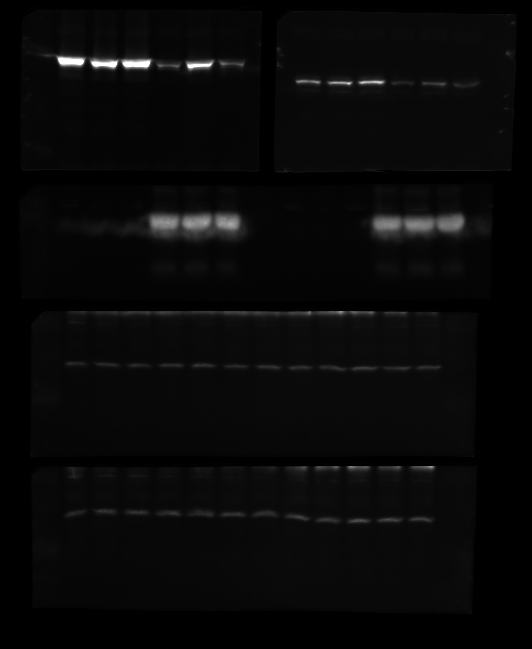

Supplement: Figure 2—source data 1. [file elife-68958-fig2-data1.zip › Figure 2ΓÇôsource data 1/Figure 2 full raw unedited blots files/2020-08-31-213817/800.TIF]

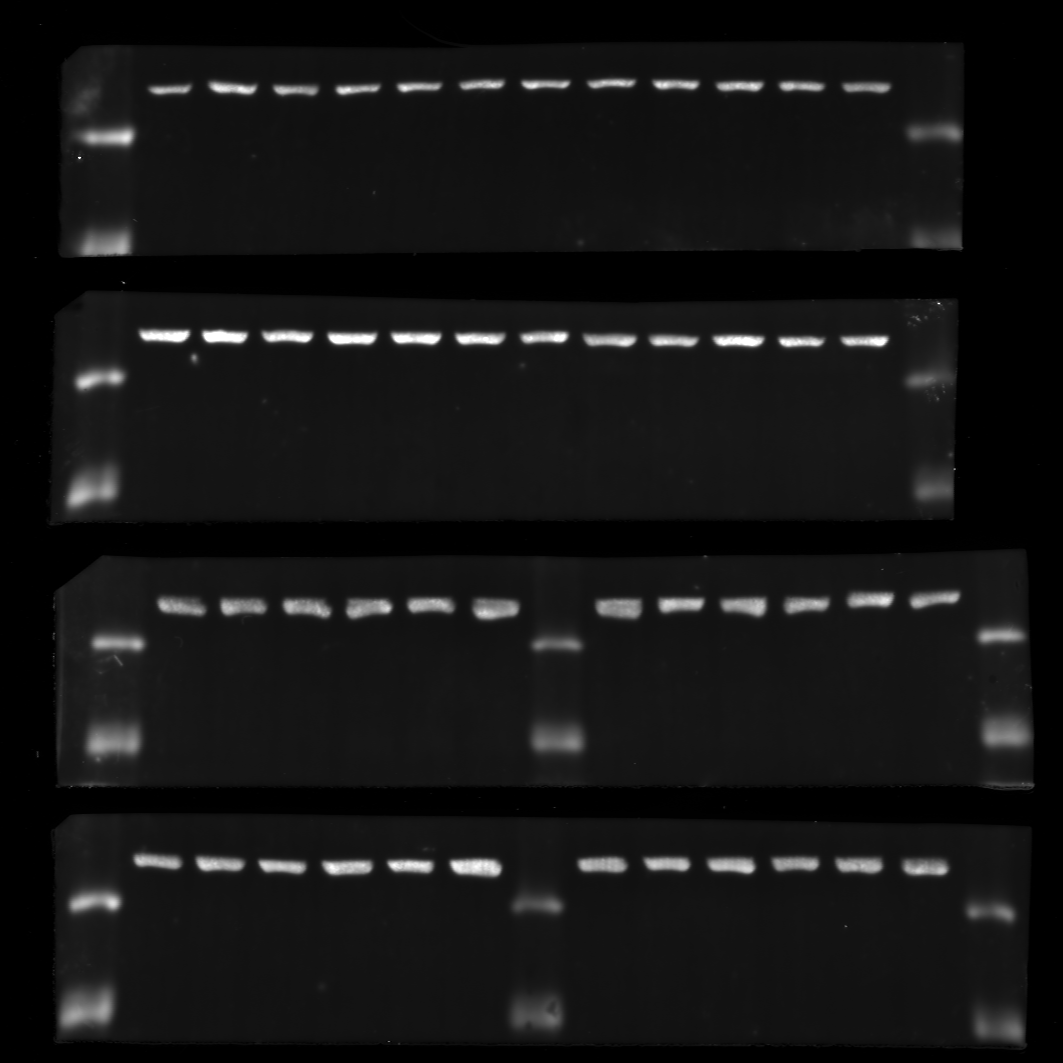

Supplement: Figure 2—source data 1. [file elife-68958-fig2-data1.zip › Figure 2ΓÇôsource data 1/Figure 2 full raw unedited blots files/2021-03-11-192357/700.TIF]

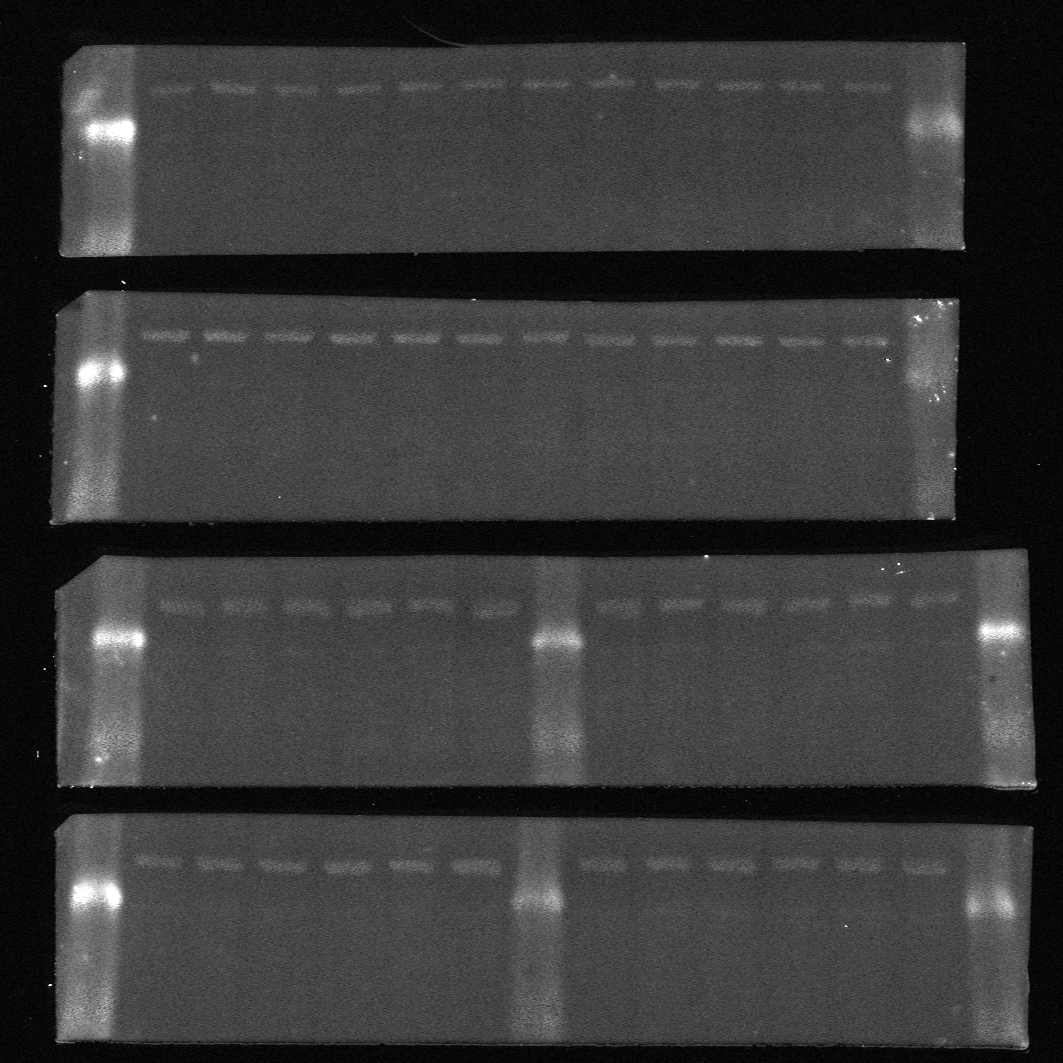

Supplement: Figure 2—source data 1. [file elife-68958-fig2-data1.zip › Figure 2ΓÇôsource data 1/Figure 2 full raw unedited blots files/2021-03-11-192357/800.TIF]

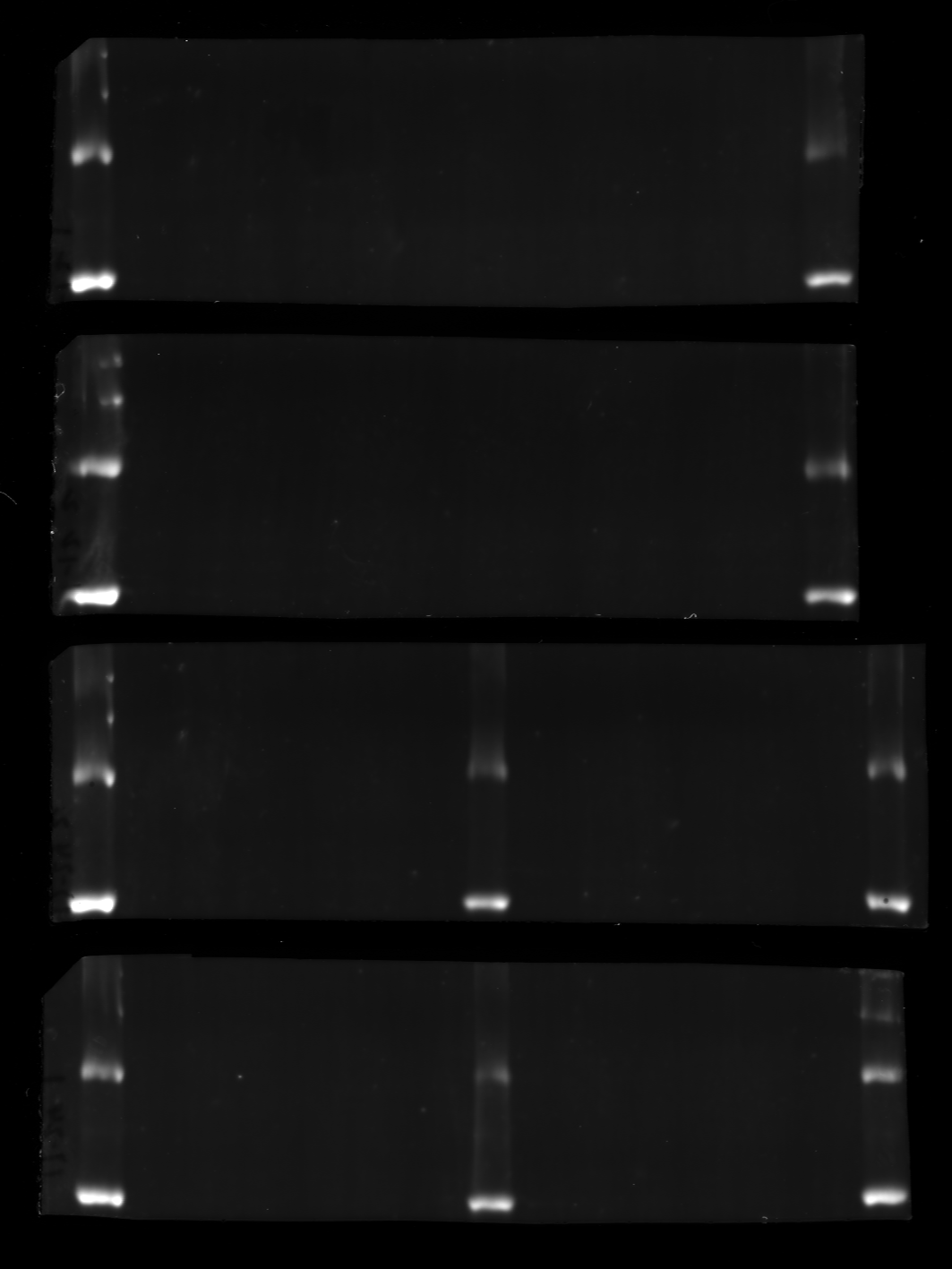

Supplement: Figure 2—source data 1. [file elife-68958-fig2-data1.zip › Figure 2ΓÇôsource data 1/Figure 2 full raw unedited blots files/2021-03-11-193735/700.TIF]

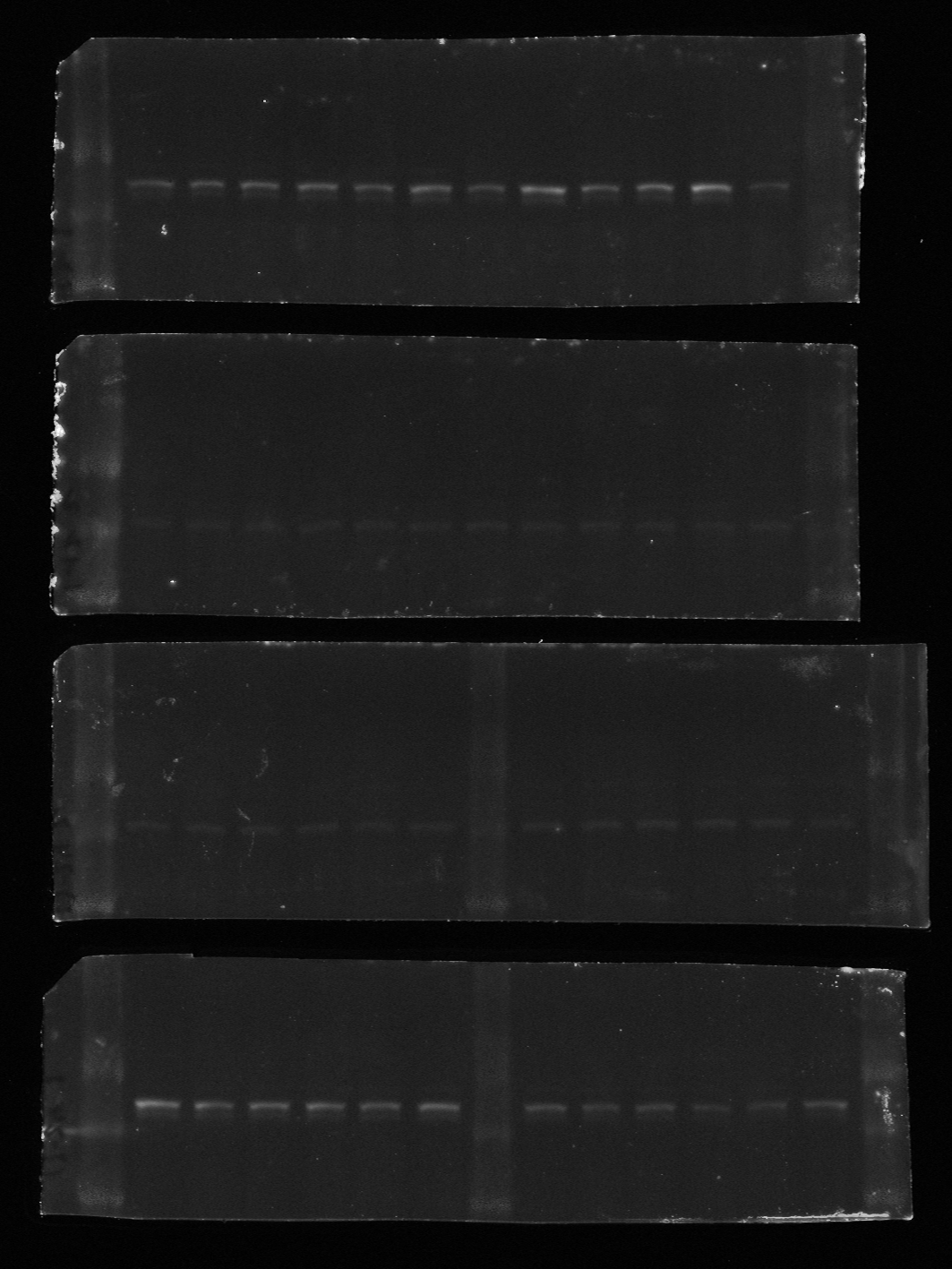

Supplement: Figure 2—source data 1. [file elife-68958-fig2-data1.zip › Figure 2ΓÇôsource data 1/Figure 2 full raw unedited blots files/2021-03-11-193735/800.TIF]

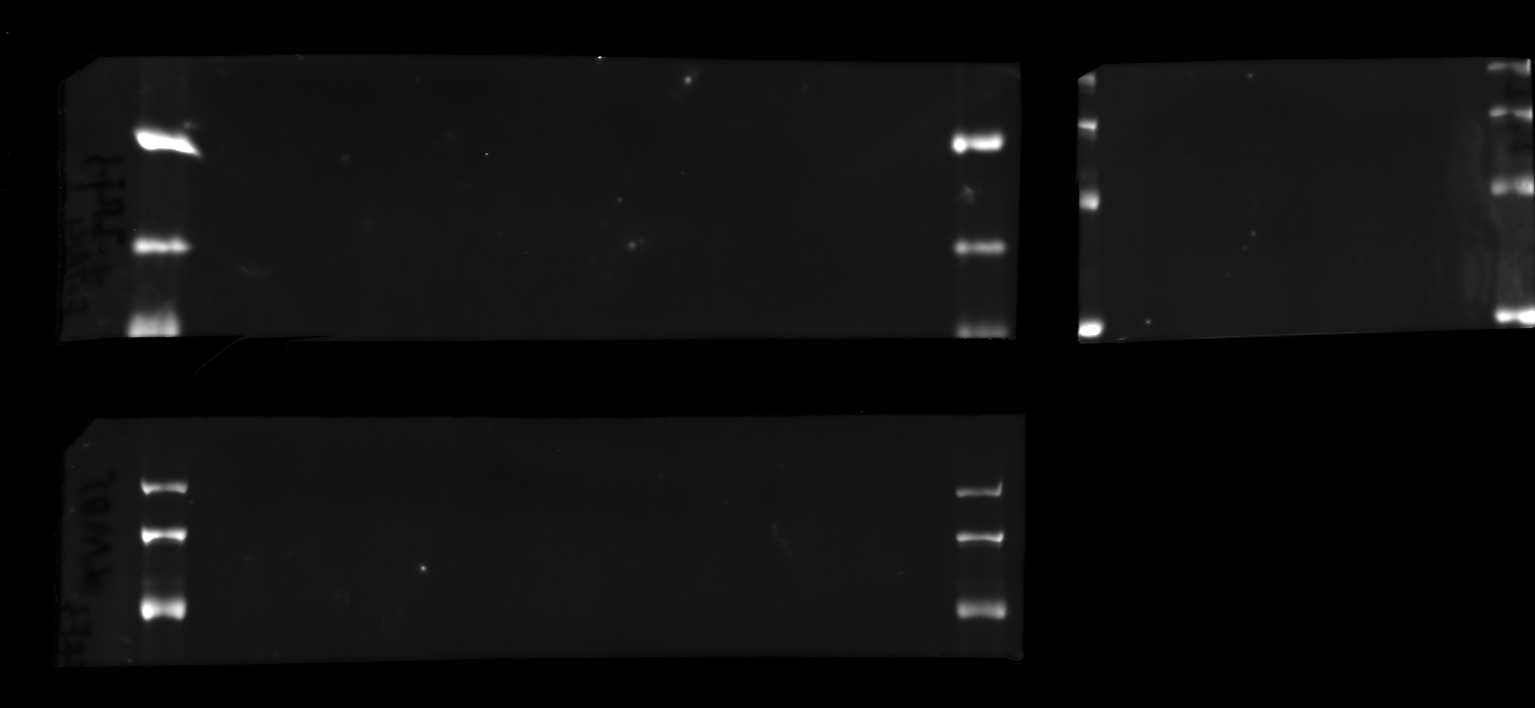

Supplement: Figure 2—source data 1. [file elife-68958-fig2-data1.zip › Figure 2ΓÇôsource data 1/Figure 2 full raw unedited blots files/2021-03-14-184539/700.TIF]

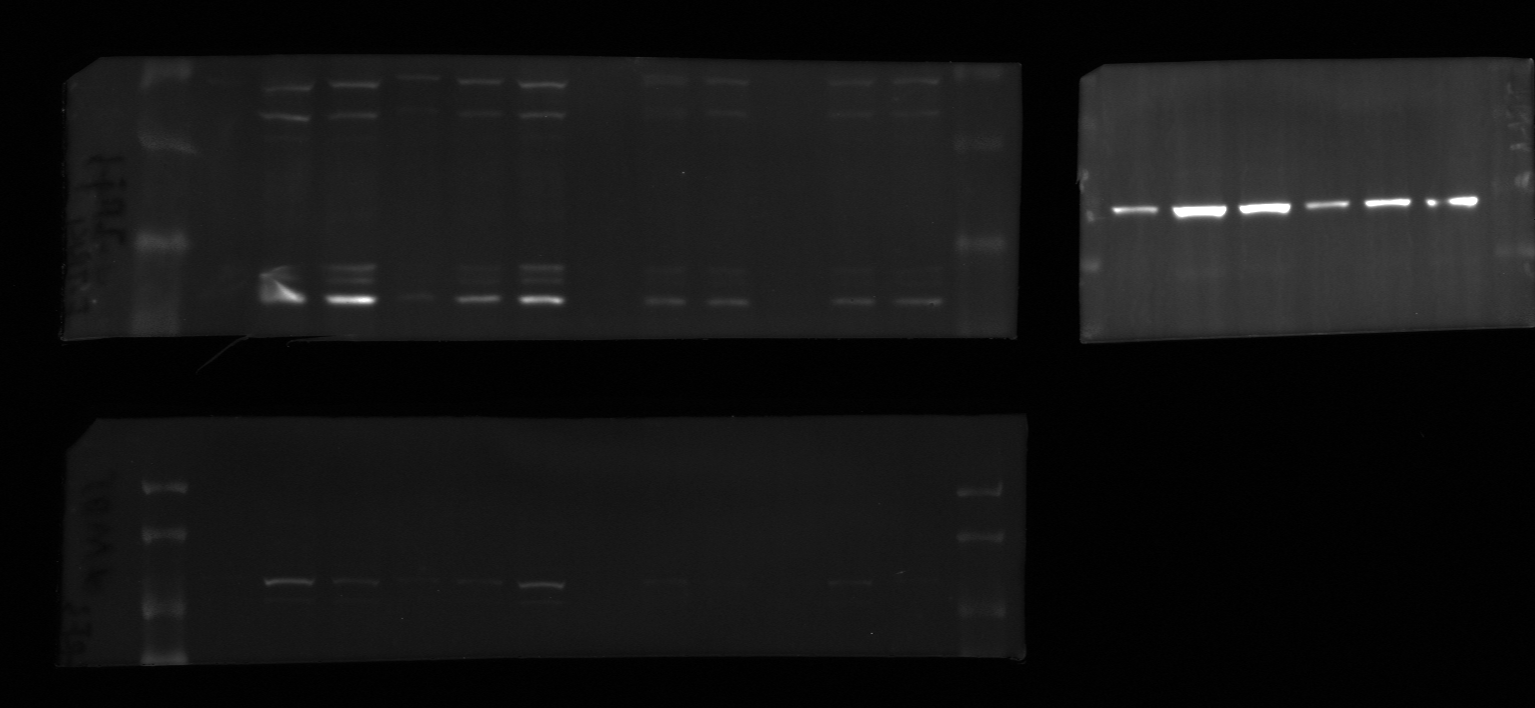

Supplement: Figure 2—source data 1. [file elife-68958-fig2-data1.zip › Figure 2ΓÇôsource data 1/Figure 2 full raw unedited blots files/2021-03-14-184539/800.TIF]

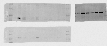

Supplement: Figure 2—source data 1. [file elife-68958-fig2-data1.zip › Figure 2ΓÇôsource data 1/Figure 2 full raw unedited blots files/2021-03-14-184539/2021-03-14-184539_a-pIRF3 12 a-pTBK1 6 a-pP65_TH.jpg]

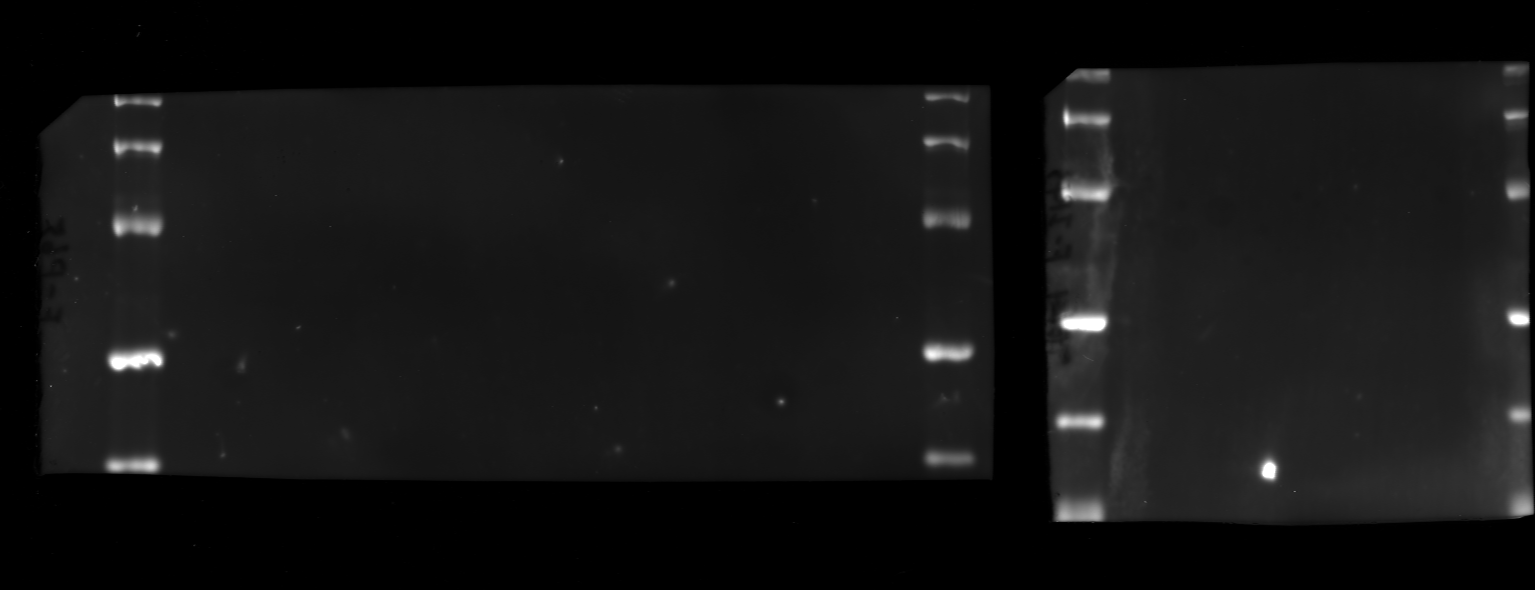

Supplement: Figure 2—source data 1. [file elife-68958-fig2-data1.zip › Figure 2ΓÇôsource data 1/Figure 2 full raw unedited blots files/2021-03-13-194244/700.TIF]

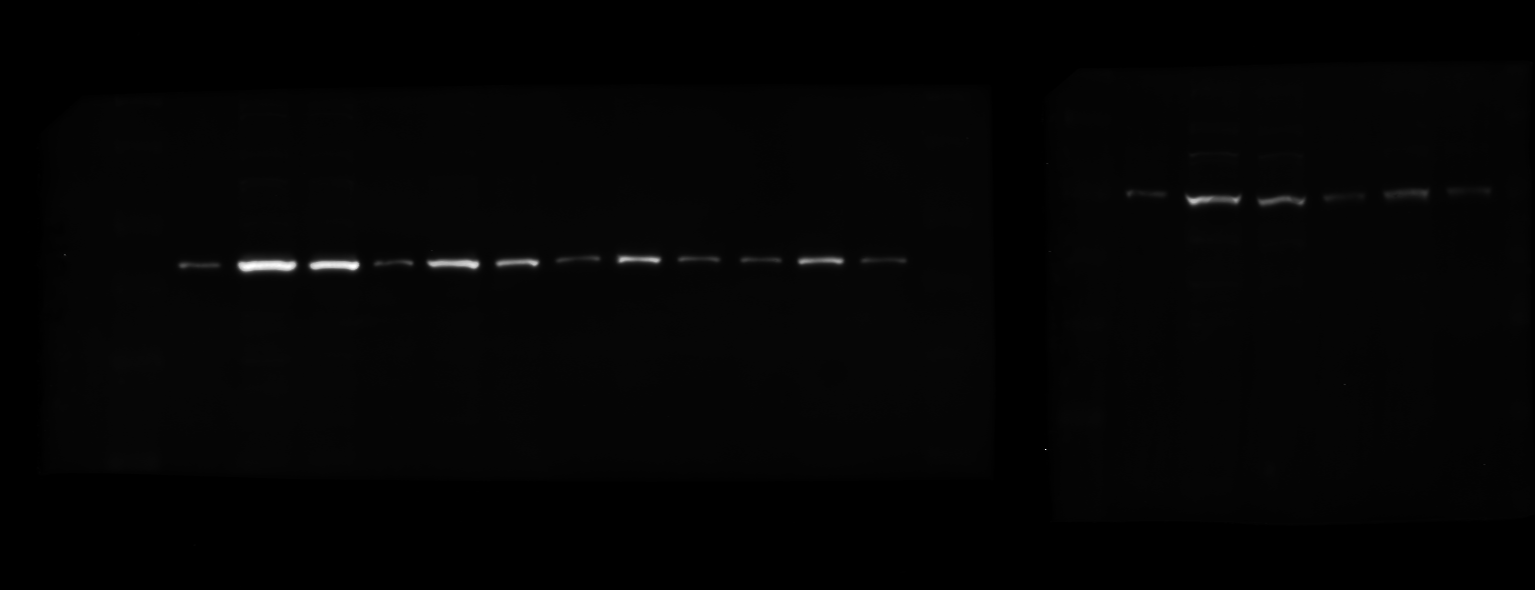

Supplement: Figure 2—source data 1. [file elife-68958-fig2-data1.zip › Figure 2ΓÇôsource data 1/Figure 2 full raw unedited blots files/2021-03-13-194244/800.TIF]

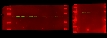

Supplement: Figure 2—source data 1. [file elife-68958-fig2-data1.zip › Figure 2ΓÇôsource data 1/Figure 2 full raw unedited blots files/2021-03-13-194244/2021-03-13-194244_a-pP65 12 pIRF3 6_TH.jpg]

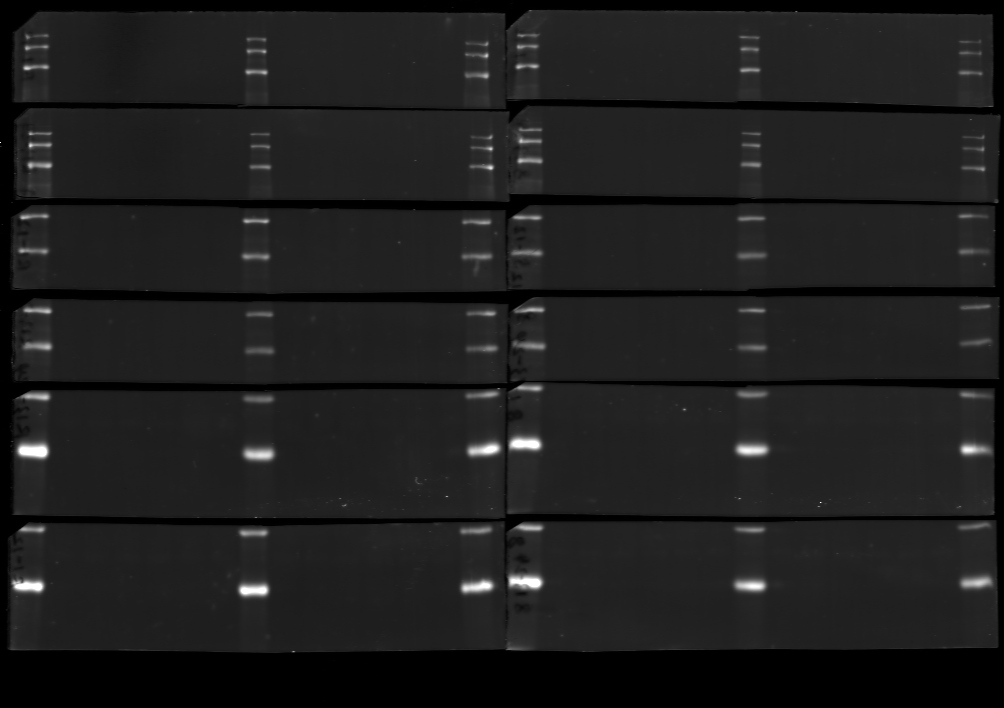

Supplement: Figure 2—source data 1. [file elife-68958-fig2-data1.zip › Figure 2ΓÇôsource data 1/Figure 2 full raw unedited blots files/2020-07-29-204333/700.TIF]

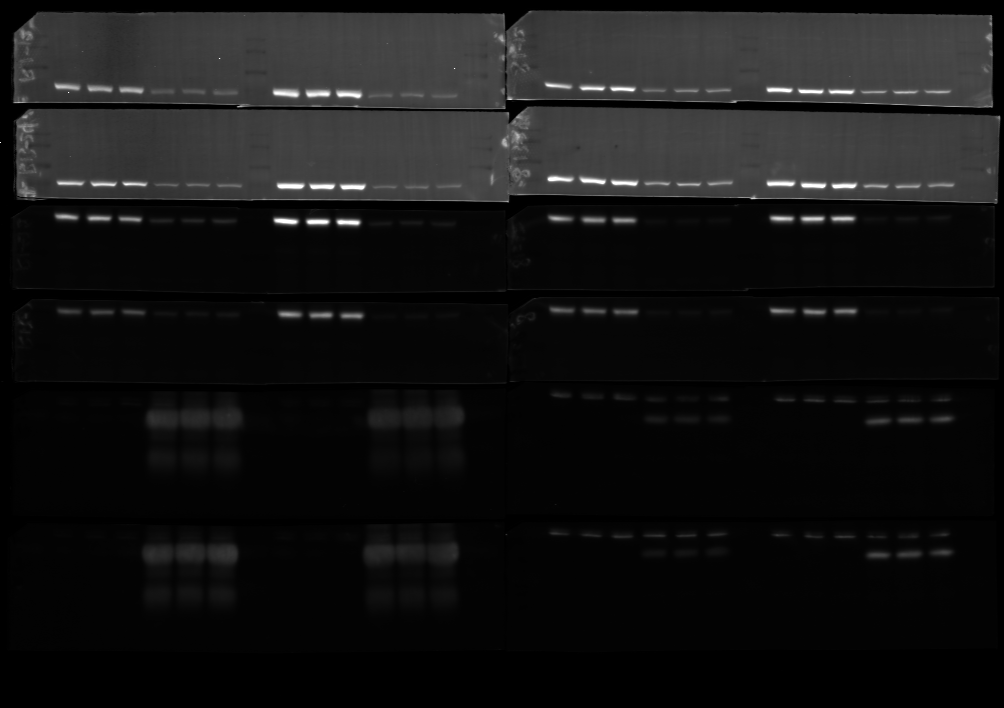

Supplement: Figure 2—source data 1. [file elife-68958-fig2-data1.zip › Figure 2ΓÇôsource data 1/Figure 2 full raw unedited blots files/2020-07-29-204333/800.TIF]

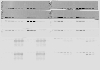

Supplement: Figure 2—source data 1. [file elife-68958-fig2-data1.zip › Figure 2ΓÇôsource data 1/Figure 2 full raw unedited blots files/2020-07-29-204333/2020-07-29-204333_a-IKKi pIKKa b pIRF3 HART3 FlagP8_TH.jpg]

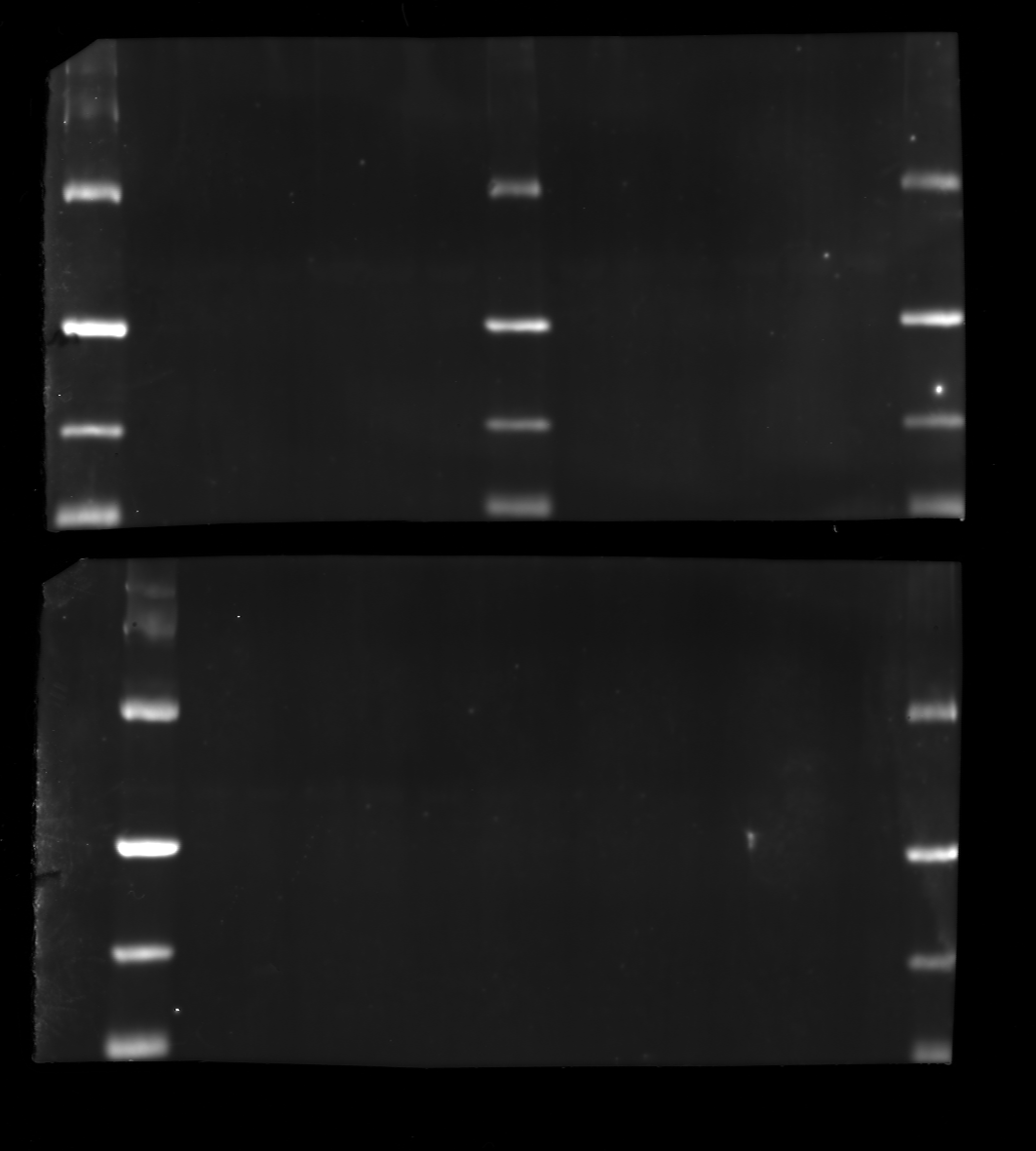

Supplement: Figure 2—source data 1. [file elife-68958-fig2-data1.zip › Figure 2ΓÇôsource data 1/Figure 2 full raw unedited blots files/2021-03-13-191448/700.TIF]

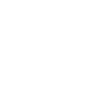

Supplement: Figure 2—source data 1. [file elife-68958-fig2-data1.zip › Figure 2ΓÇôsource data 1/Figure 2 full raw unedited blots files/2021-03-13-191448/2021-03-13-191448_a-pIRF3_TH.jpg]

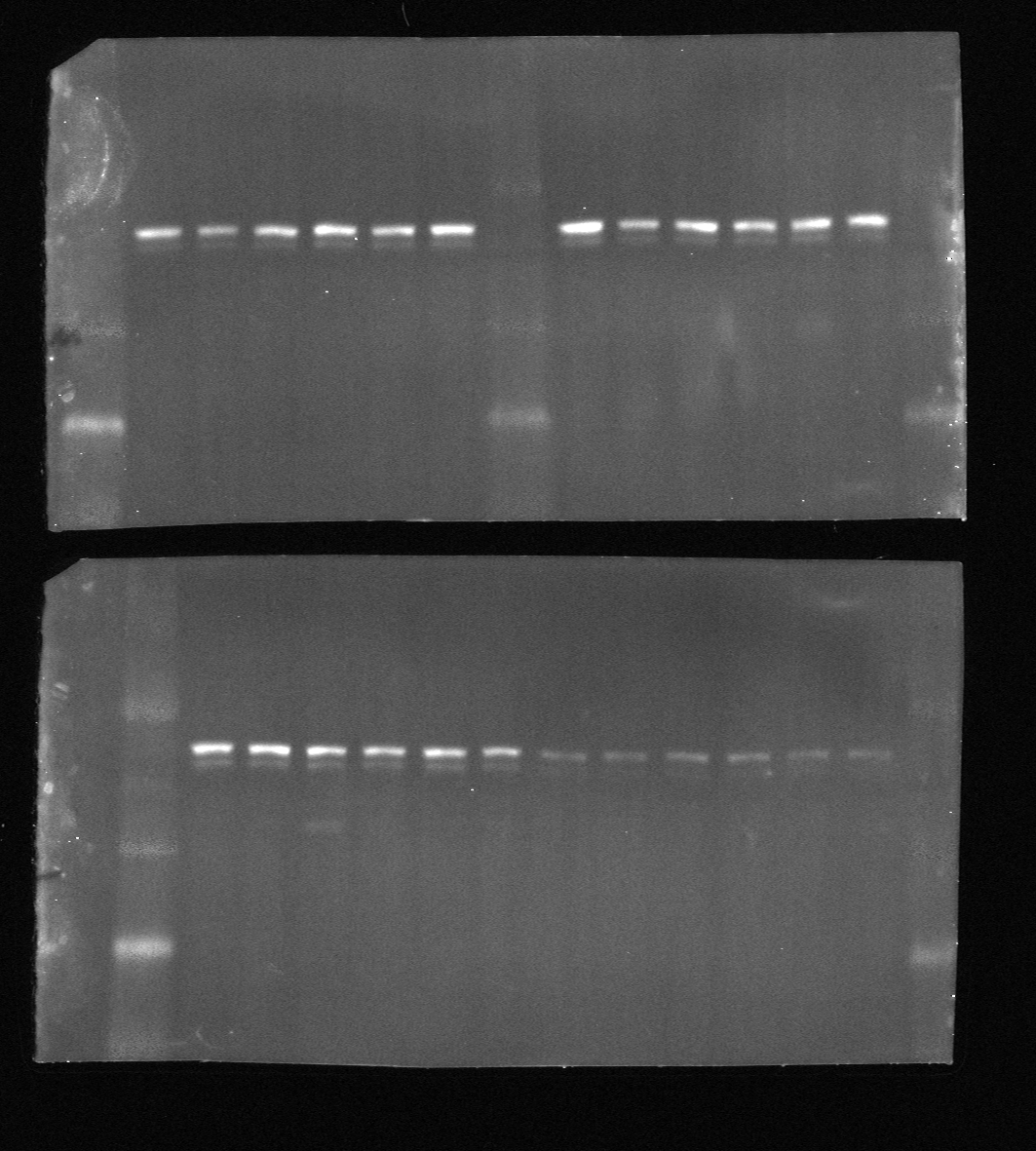

Supplement: Figure 2—source data 1. [file elife-68958-fig2-data1.zip › Figure 2ΓÇôsource data 1/Figure 2 full raw unedited blots files/2021-03-13-191448/800.TIF]

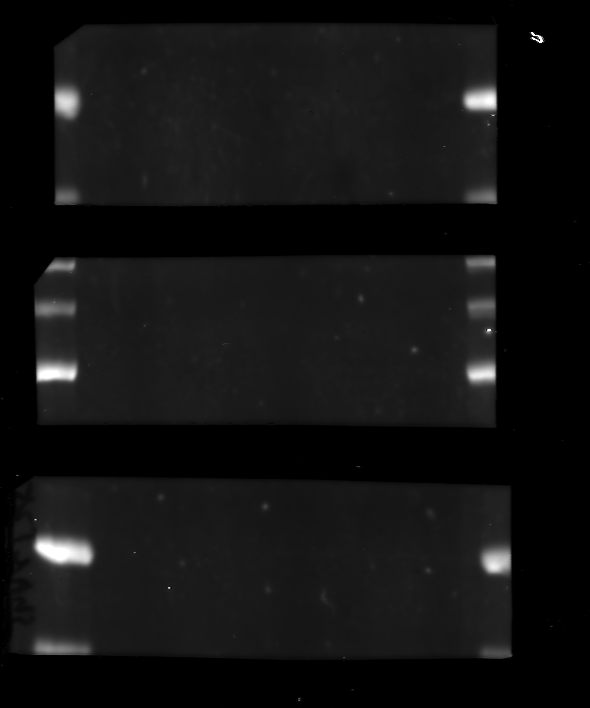

Supplement: Figure 3—source data 1. [file elife-68958-fig3-data1.zip › Figure 3ΓÇôsource data 1/Figure 3 full raw unedited blots files/original_files for B/2021-06-04-155118/700.TIF]

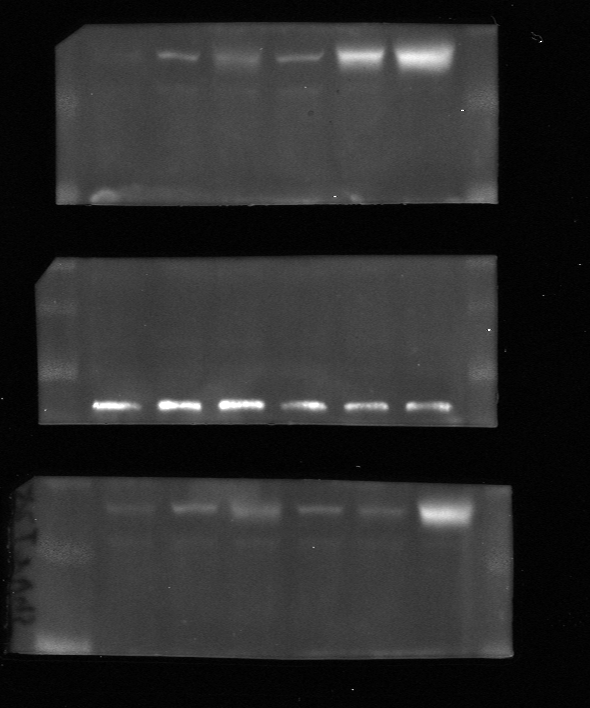

Supplement: Figure 3—source data 1. [file elife-68958-fig3-data1.zip › Figure 3ΓÇôsource data 1/Figure 3 full raw unedited blots files/original_files for B/2021-06-04-155118/800.TIF]

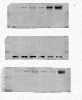

Supplement: Figure 3—source data 1. [file elife-68958-fig3-data1.zip › Figure 3ΓÇôsource data 1/Figure 3 full raw unedited blots files/original_files for B/2021-06-04-155118/2021-06-04-155118_NCSHT25 pIRF3_TH.jpg]

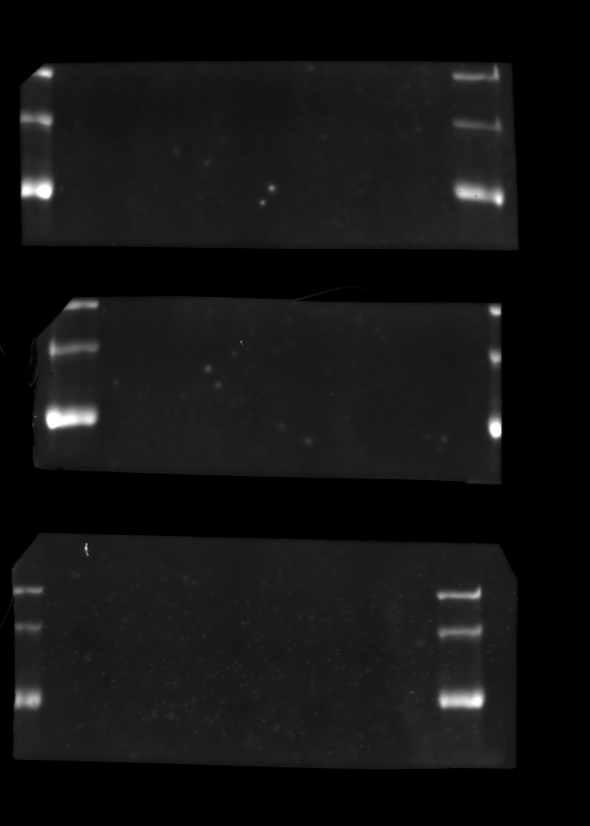

Supplement: Figure 3—source data 1. [file elife-68958-fig3-data1.zip › Figure 3ΓÇôsource data 1/Figure 3 full raw unedited blots files/original_files for B/2021-06-02-164308/700.TIF]

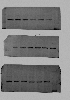

Supplement: Figure 3—source data 1. [file elife-68958-fig3-data1.zip › Figure 3ΓÇôsource data 1/Figure 3 full raw unedited blots files/original_files for B/2021-06-02-164308/2021-06-02-164308_TRIM25 MDA5_TH.jpg]

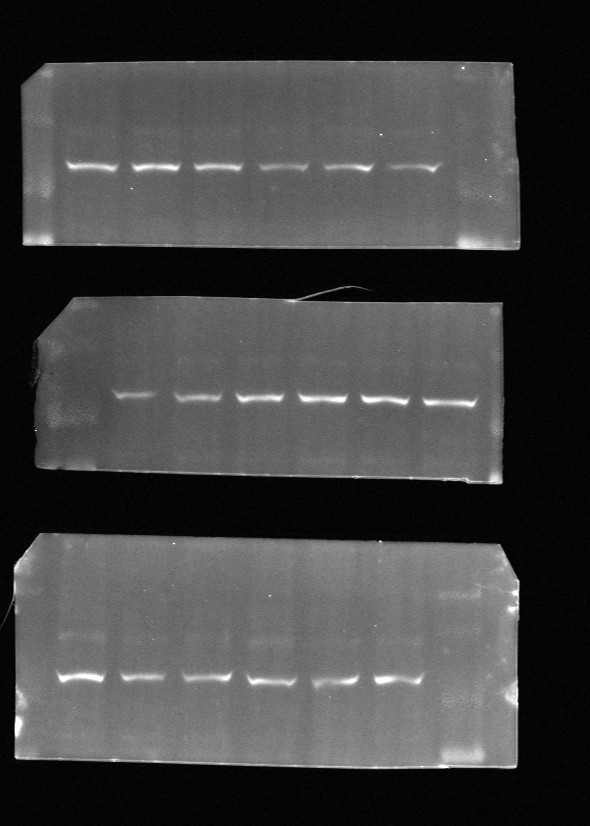

Supplement: Figure 3—source data 1. [file elife-68958-fig3-data1.zip › Figure 3ΓÇôsource data 1/Figure 3 full raw unedited blots files/original_files for B/2021-06-02-164308/800.TIF]

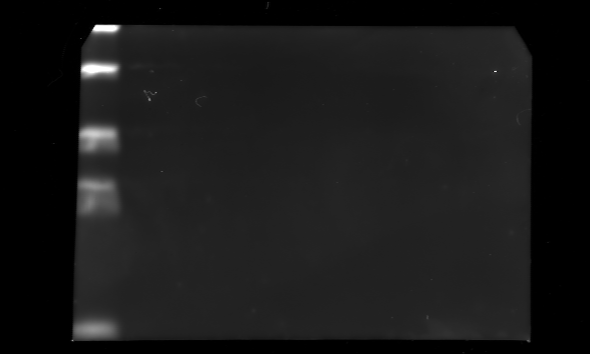

Supplement: Figure 3—source data 1. [file elife-68958-fig3-data1.zip › Figure 3ΓÇôsource data 1/Figure 3 full raw unedited blots files/original_files for B/2021-06-24-102636/700.TIF]

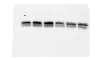

Supplement: Figure 3—source data 1. [file elife-68958-fig3-data1.zip › Figure 3ΓÇôsource data 1/Figure 3 full raw unedited blots files/original_files for B/2021-06-24-102636/2021-06-24-102636_2_TH.jpg]

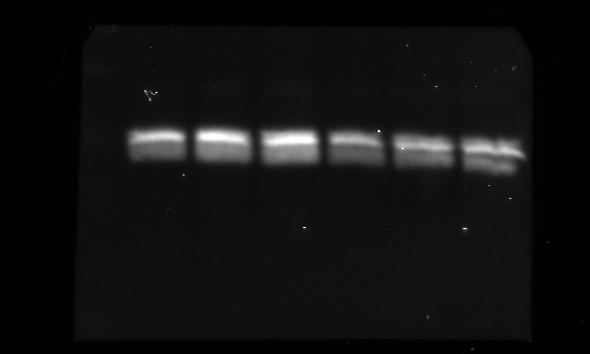

Supplement: Figure 3—source data 1. [file elife-68958-fig3-data1.zip › Figure 3ΓÇôsource data 1/Figure 3 full raw unedited blots files/original_files for B/2021-06-24-102636/800.TIF]

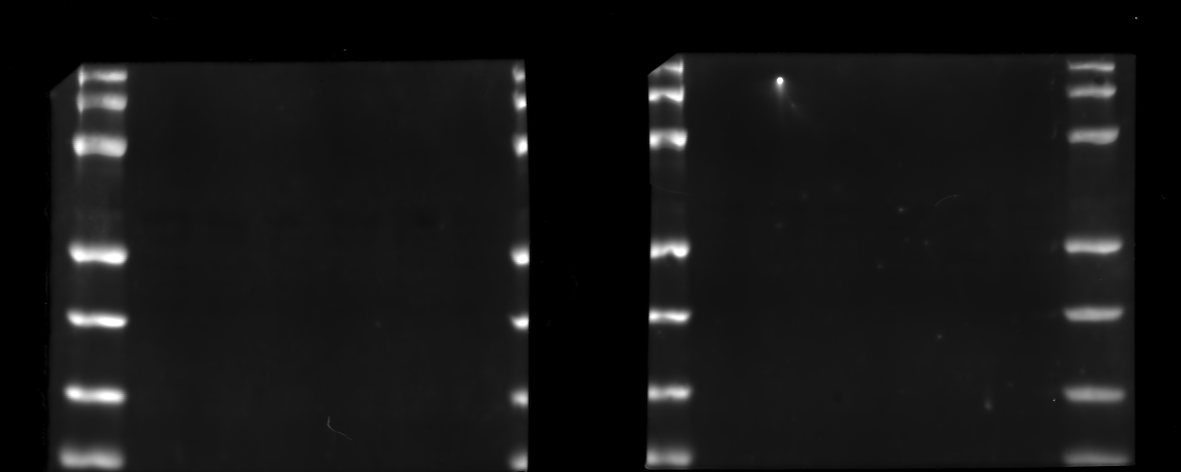

Supplement: Figure 3—source data 1. [file elife-68958-fig3-data1.zip › Figure 3ΓÇôsource data 1/Figure 3 full raw unedited blots files/original_files for B/2021-06-13-185029/700.TIF]

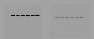

Supplement: Figure 3—source data 1. [file elife-68958-fig3-data1.zip › Figure 3ΓÇôsource data 1/Figure 3 full raw unedited blots files/original_files for B/2021-06-13-185029/2021-06-13-185029_P65 IRF3_TH.jpg]

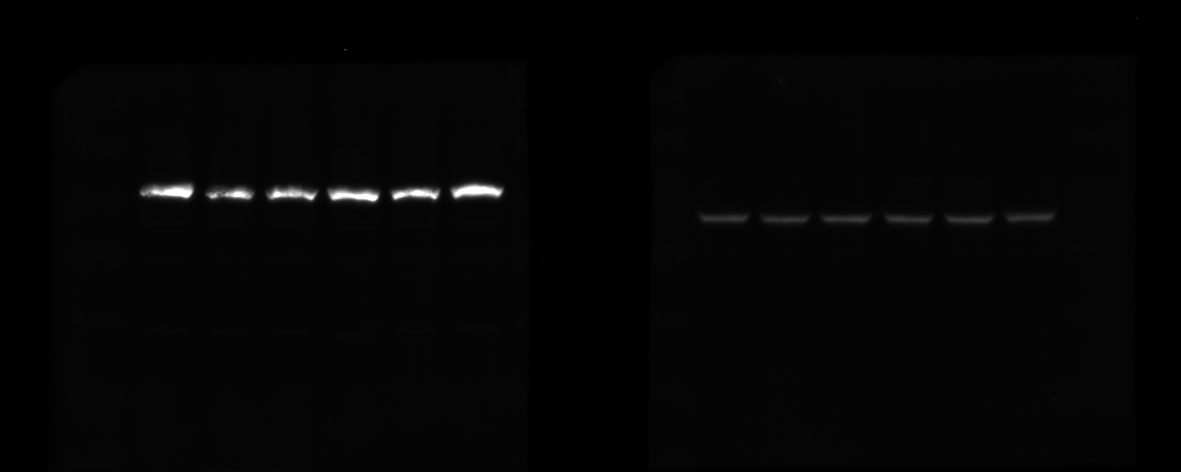

Supplement: Figure 3—source data 1. [file elife-68958-fig3-data1.zip › Figure 3ΓÇôsource data 1/Figure 3 full raw unedited blots files/original_files for B/2021-06-13-185029/800.TIF]

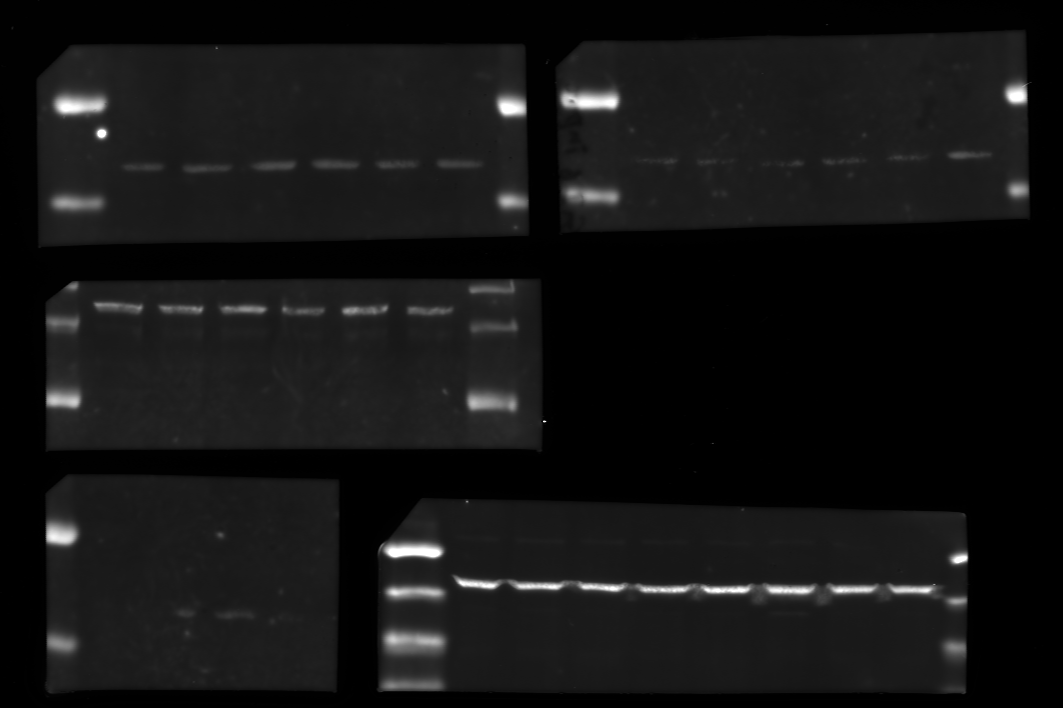

Supplement: Figure 3—source data 1. [file elife-68958-fig3-data1.zip › Figure 3ΓÇôsource data 1/Figure 3 full raw unedited blots files/original_files for B/2021-06-05-164811/700.TIF]

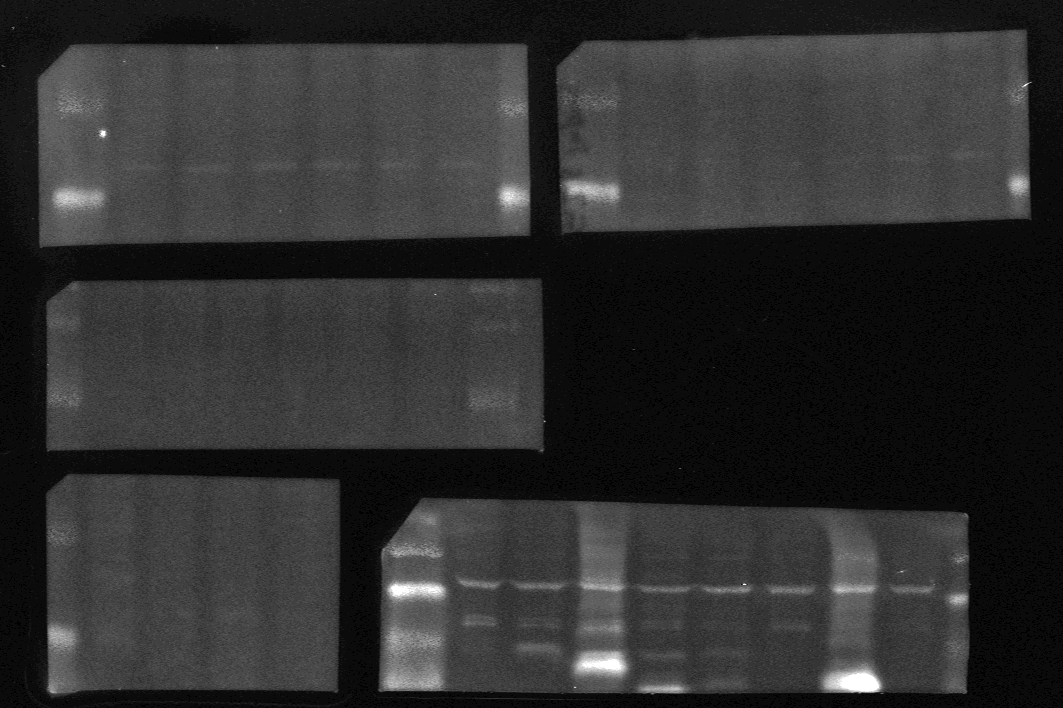

Supplement: Figure 3—source data 1. [file elife-68958-fig3-data1.zip › Figure 3ΓÇôsource data 1/Figure 3 full raw unedited blots files/original_files for B/2021-06-05-164811/800.TIF]

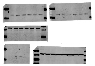

Supplement: Figure 3—source data 1. [file elife-68958-fig3-data1.zip › Figure 3ΓÇôsource data 1/Figure 3 full raw unedited blots files/original_files for B/2021-06-05-164811/2021-06-05-164811_ACTIN_TH.jpg]

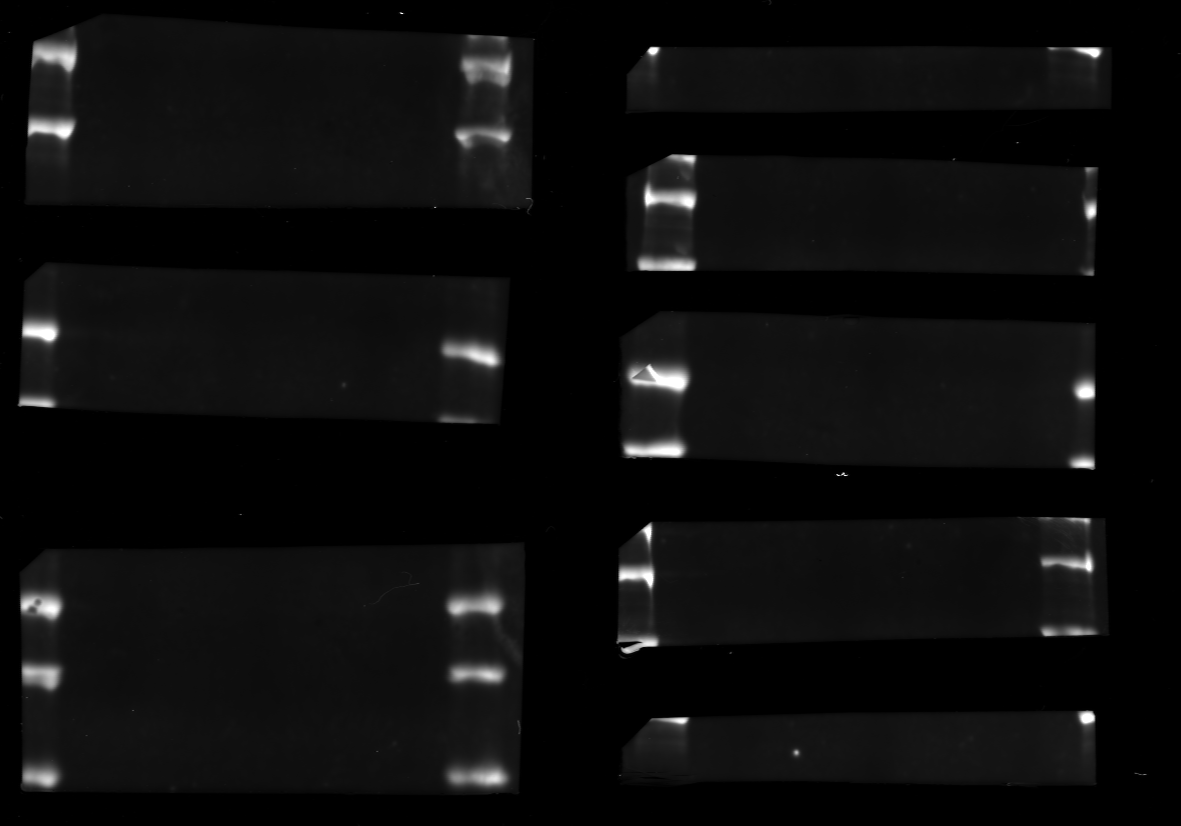

Supplement: Figure 3—source data 1. [file elife-68958-fig3-data1.zip › Figure 3ΓÇôsource data 1/Figure 3 full raw unedited blots files/original_files for B/2021-06-07-114205/700.TIF]

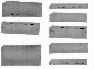

Supplement: Figure 3—source data 1. [file elife-68958-fig3-data1.zip › Figure 3ΓÇôsource data 1/Figure 3 full raw unedited blots files/original_files for B/2021-06-07-114205/2021-06-07-114205_pIKKab pP65 pIRF3_TH.jpg]

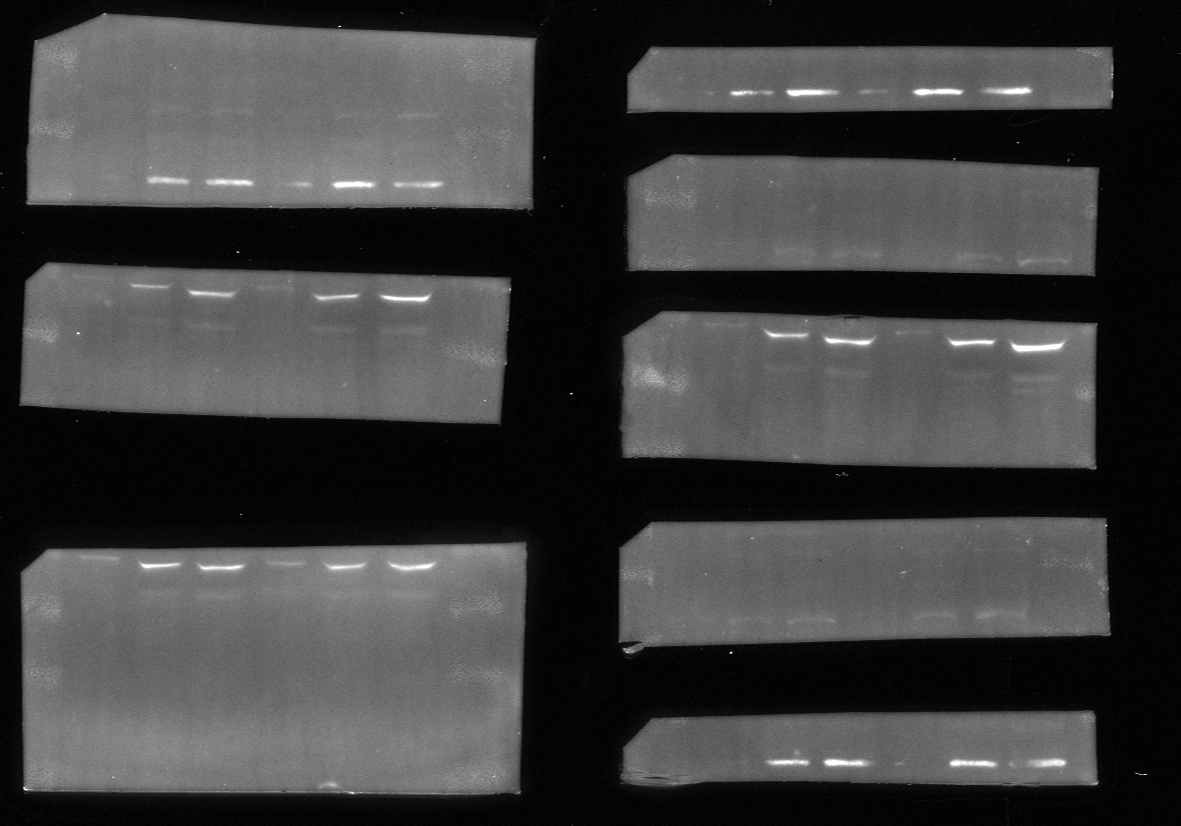

Supplement: Figure 3—source data 1. [file elife-68958-fig3-data1.zip › Figure 3ΓÇôsource data 1/Figure 3 full raw unedited blots files/original_files for B/2021-06-07-114205/800.TIF]

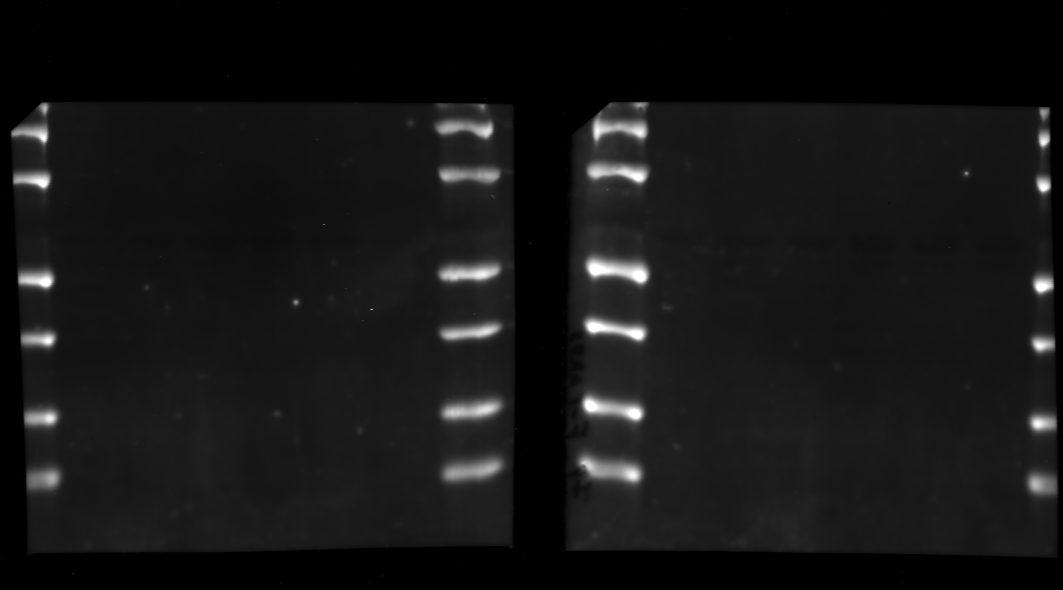

Supplement: Figure 3—source data 1. [file elife-68958-fig3-data1.zip › Figure 3ΓÇôsource data 1/Figure 3 full raw unedited blots files/original_files for B/2021-06-12-145956/700.TIF]

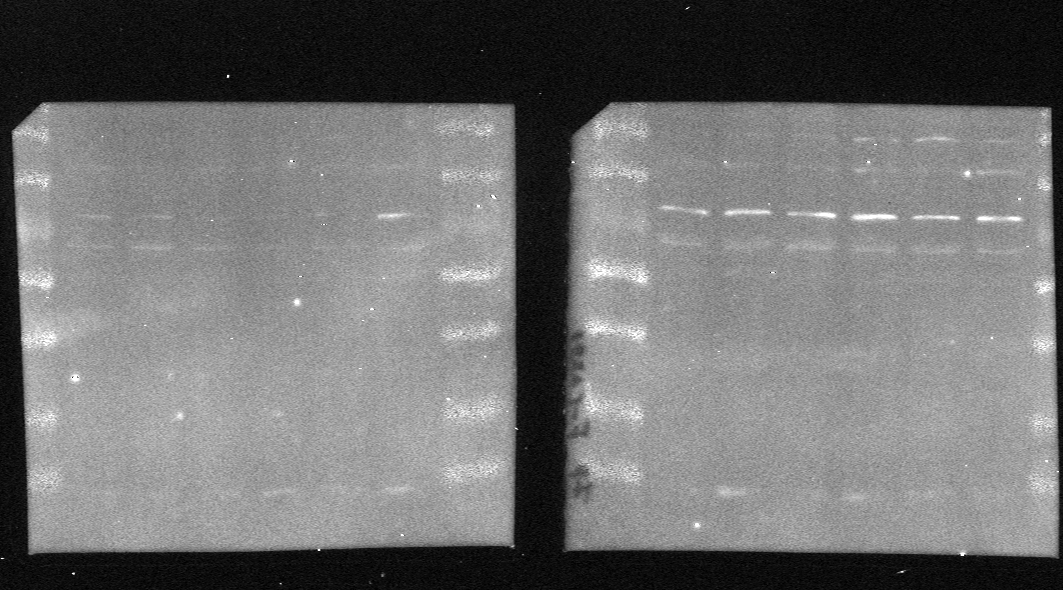

Supplement: Figure 3—source data 1. [file elife-68958-fig3-data1.zip › Figure 3ΓÇôsource data 1/Figure 3 full raw unedited blots files/original_files for B/2021-06-12-145956/800.TIF]

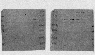

Supplement: Figure 3—source data 1. [file elife-68958-fig3-data1.zip › Figure 3ΓÇôsource data 1/Figure 3 full raw unedited blots files/original_files for B/2021-06-12-145956/2021-06-12-145956_4_TH.jpg]

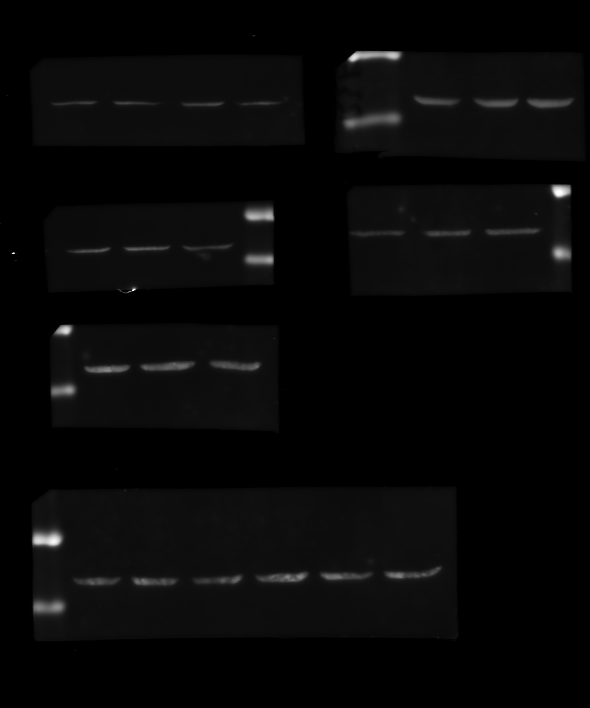

Supplement: Figure 3—source data 1. [file elife-68958-fig3-data1.zip › Figure 3ΓÇôsource data 1/Figure 3 full raw unedited blots files/original_files for A/2021-05-31-162639/700.TIF]

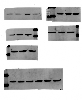

Supplement: Figure 3—source data 1. [file elife-68958-fig3-data1.zip › Figure 3ΓÇôsource data 1/Figure 3 full raw unedited blots files/original_files for A/2021-05-31-162639/2021-05-31-162639_actin_TH.jpg]

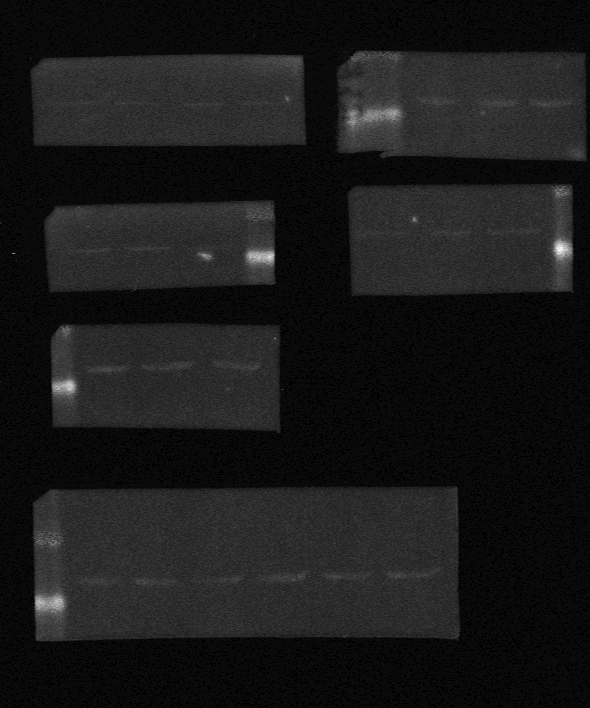

Supplement: Figure 3—source data 1. [file elife-68958-fig3-data1.zip › Figure 3ΓÇôsource data 1/Figure 3 full raw unedited blots files/original_files for A/2021-05-31-162639/800.TIF]

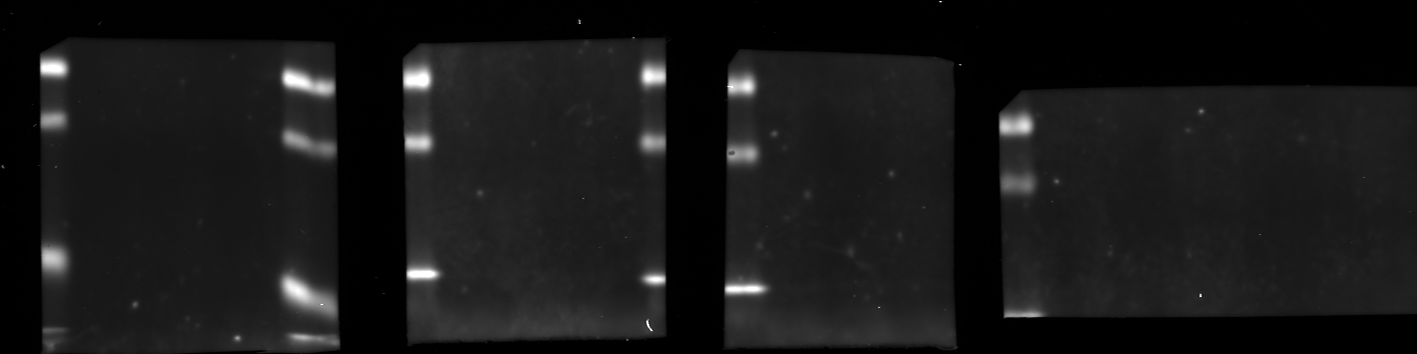

Supplement: Figure 3—source data 1. [file elife-68958-fig3-data1.zip › Figure 3ΓÇôsource data 1/Figure 3 full raw unedited blots files/original_files for A/2021-05-31-171228/700.TIF]

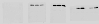

Supplement: Figure 3—source data 1. [file elife-68958-fig3-data1.zip › Figure 3ΓÇôsource data 1/Figure 3 full raw unedited blots files/original_files for A/2021-05-31-171228/2021-05-31-171228_RT3NCKD 0200400_TH.jpg]

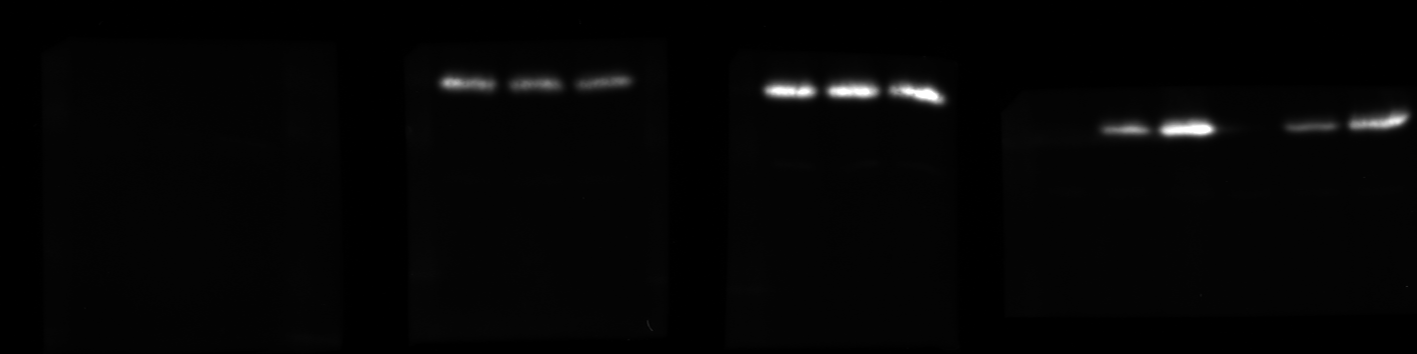

Supplement: Figure 3—source data 1. [file elife-68958-fig3-data1.zip › Figure 3ΓÇôsource data 1/Figure 3 full raw unedited blots files/original_files for A/2021-05-31-171228/800.TIF]

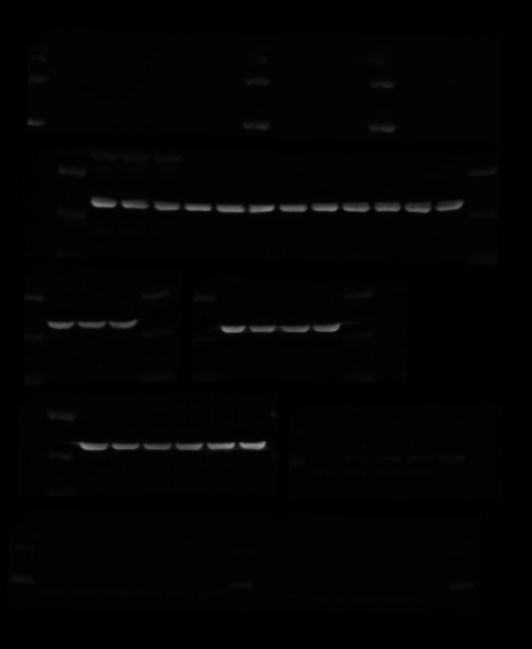

Supplement: Figure 4—source data 1. [file elife-68958-fig4-data1.zip › Figure 4ΓÇôsource data 1/Figure 4 full raw unedited blots files/original_files for C/2020-08-20-222444/700.TIF]

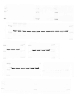

Supplement: Figure 4—source data 1. [file elife-68958-fig4-data1.zip › Figure 4ΓÇôsource data 1/Figure 4 full raw unedited blots files/original_files for C/2020-08-20-222444/2020-08-20-222444_a-T25 WTand KD actin GST RI_TH.jpg]

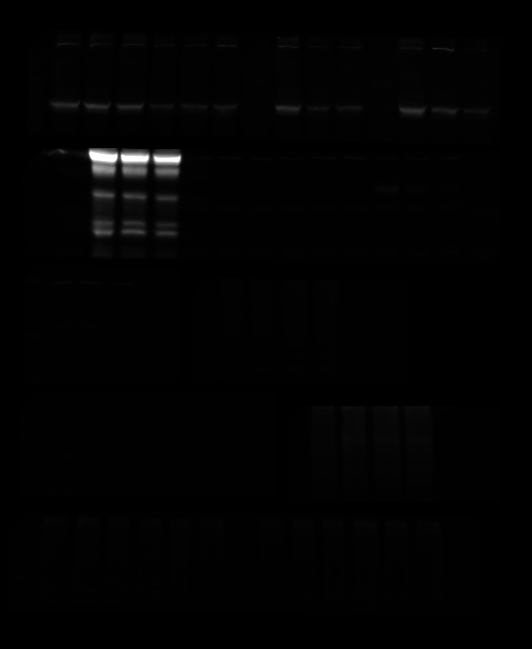

Supplement: Figure 4—source data 1. [file elife-68958-fig4-data1.zip › Figure 4ΓÇôsource data 1/Figure 4 full raw unedited blots files/original_files for C/2020-08-20-222444/800.TIF]

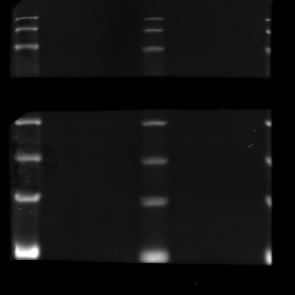

Supplement: Figure 4—source data 1. [file elife-68958-fig4-data1.zip › Figure 4ΓÇôsource data 1/Figure 4 full raw unedited blots files/original_files for C/2020-07-05-224341/700.TIF]

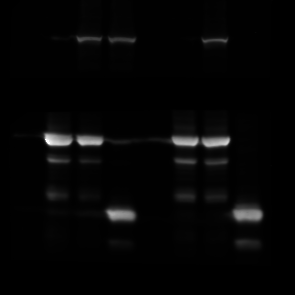

Supplement: Figure 4—source data 1. [file elife-68958-fig4-data1.zip › Figure 4ΓÇôsource data 1/Figure 4 full raw unedited blots files/original_files for C/2020-07-05-224341/800.TIF]

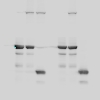

Supplement: Figure 4—source data 1. [file elife-68958-fig4-data1.zip › Figure 4ΓÇôsource data 1/Figure 4 full raw unedited blots files/original_files for C/2020-07-05-224341/2020-07-05-224341_a-F-RIGI GFP-RT3 input IP_TH.jpg]

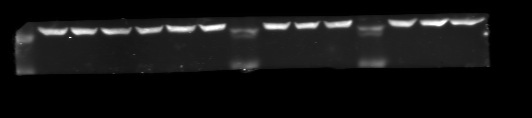

Supplement: Figure 4—source data 1. [file elife-68958-fig4-data1.zip › Figure 4ΓÇôsource data 1/Figure 4 full raw unedited blots files/original_files for B/2020-08-22-190755/700.TIF]

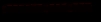

Supplement: Figure 4—source data 1. [file elife-68958-fig4-data1.zip › Figure 4ΓÇôsource data 1/Figure 4 full raw unedited blots files/original_files for B/2020-08-22-190755/2020-08-22-190755_actin_TH.jpg]

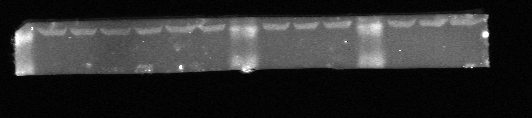

Supplement: Figure 4—source data 1. [file elife-68958-fig4-data1.zip › Figure 4ΓÇôsource data 1/Figure 4 full raw unedited blots files/original_files for B/2020-08-22-190755/800.TIF]

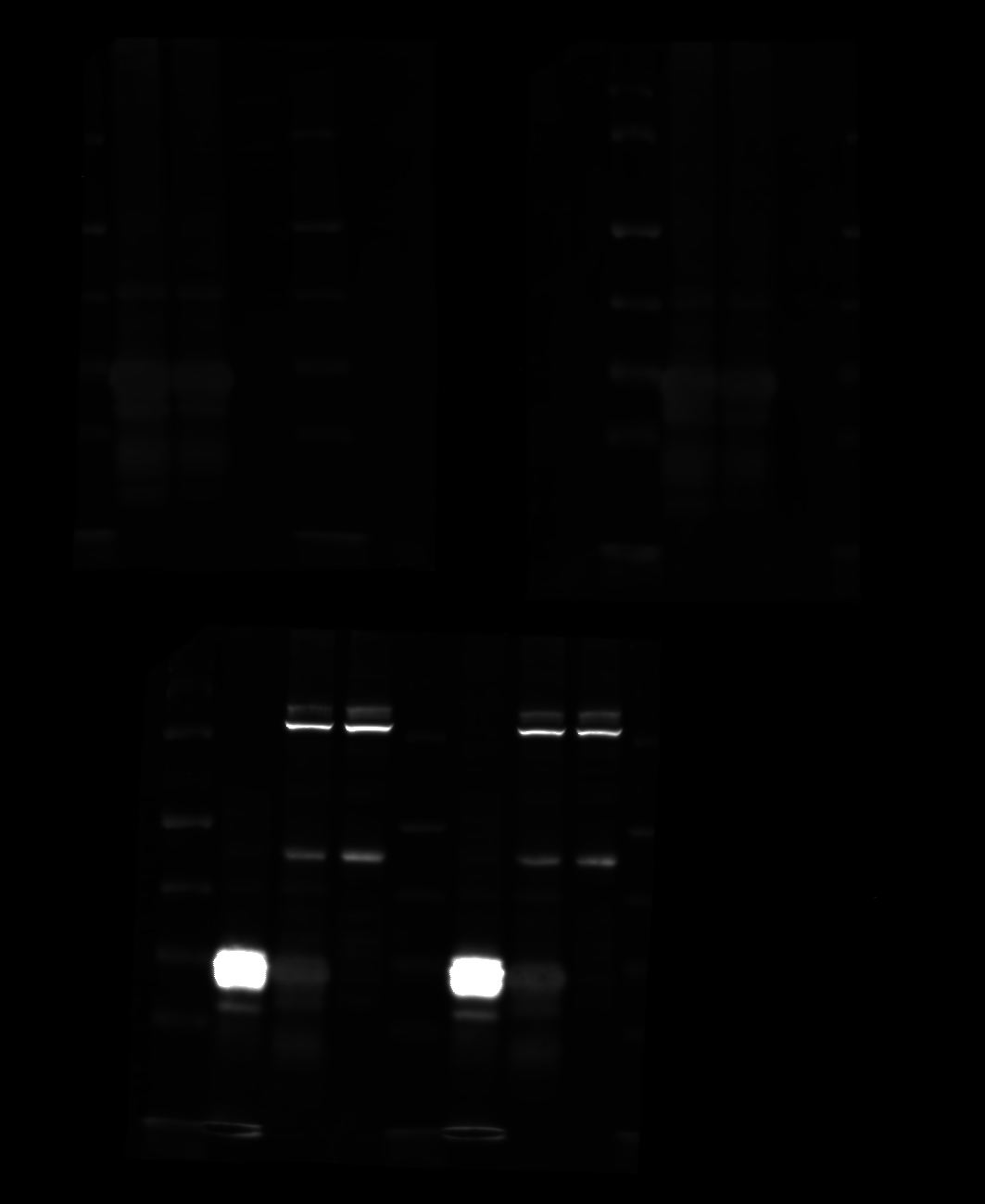

Supplement: Figure 4—source data 1. [file elife-68958-fig4-data1.zip › Figure 4ΓÇôsource data 1/Figure 4 full raw unedited blots files/original_files for B/2021-03-04-213536/700.TIF]

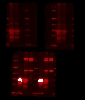

Supplement: Figure 4—source data 1. [file elife-68958-fig4-data1.zip › Figure 4ΓÇôsource data 1/Figure 4 full raw unedited blots files/original_files for B/2021-03-04-213536/2021-03-04-213536_a-GFP-RT3_TH.jpg]

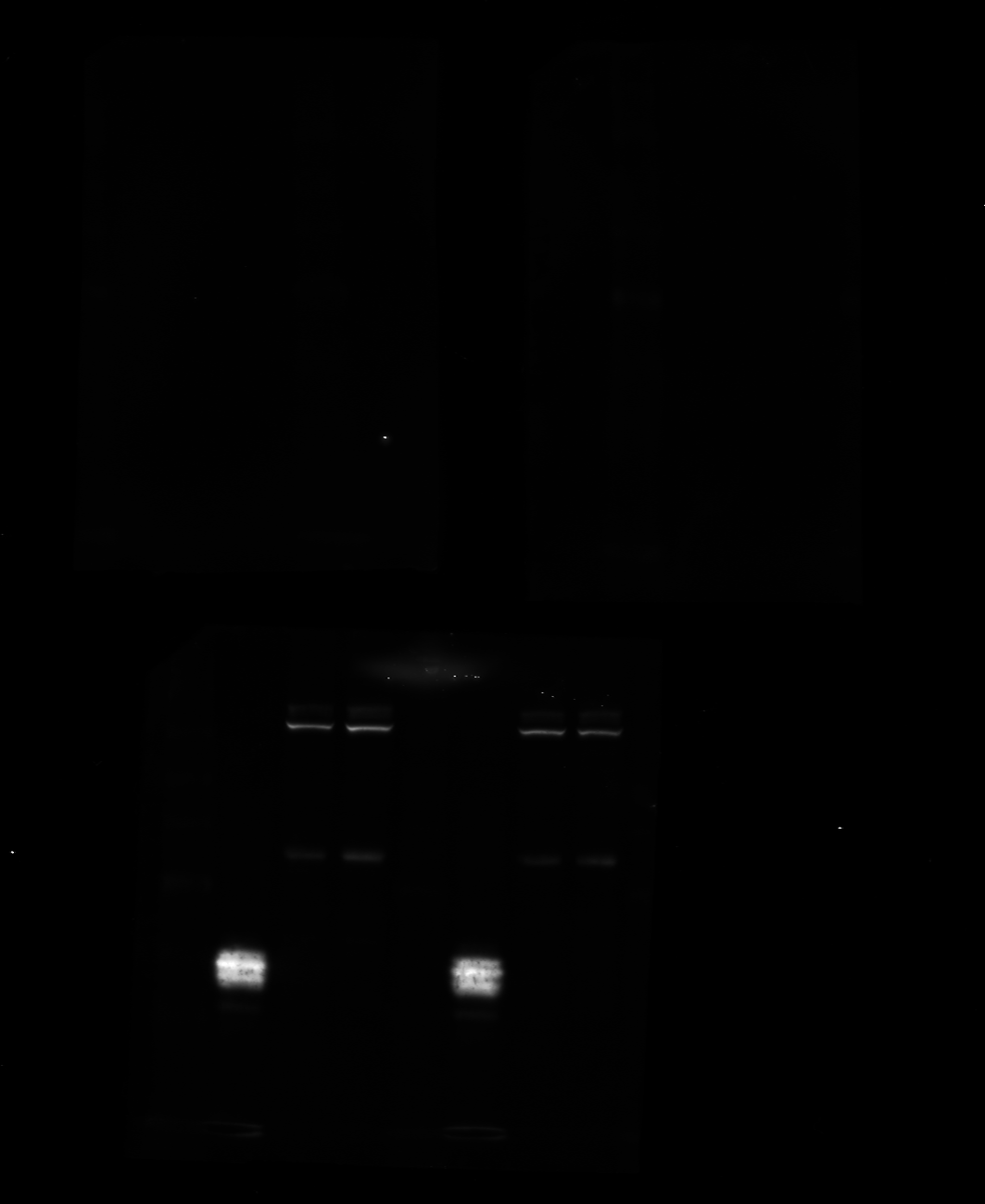

Supplement: Figure 4—source data 1. [file elife-68958-fig4-data1.zip › Figure 4ΓÇôsource data 1/Figure 4 full raw unedited blots files/original_files for B/2021-03-04-213536/800.TIF]

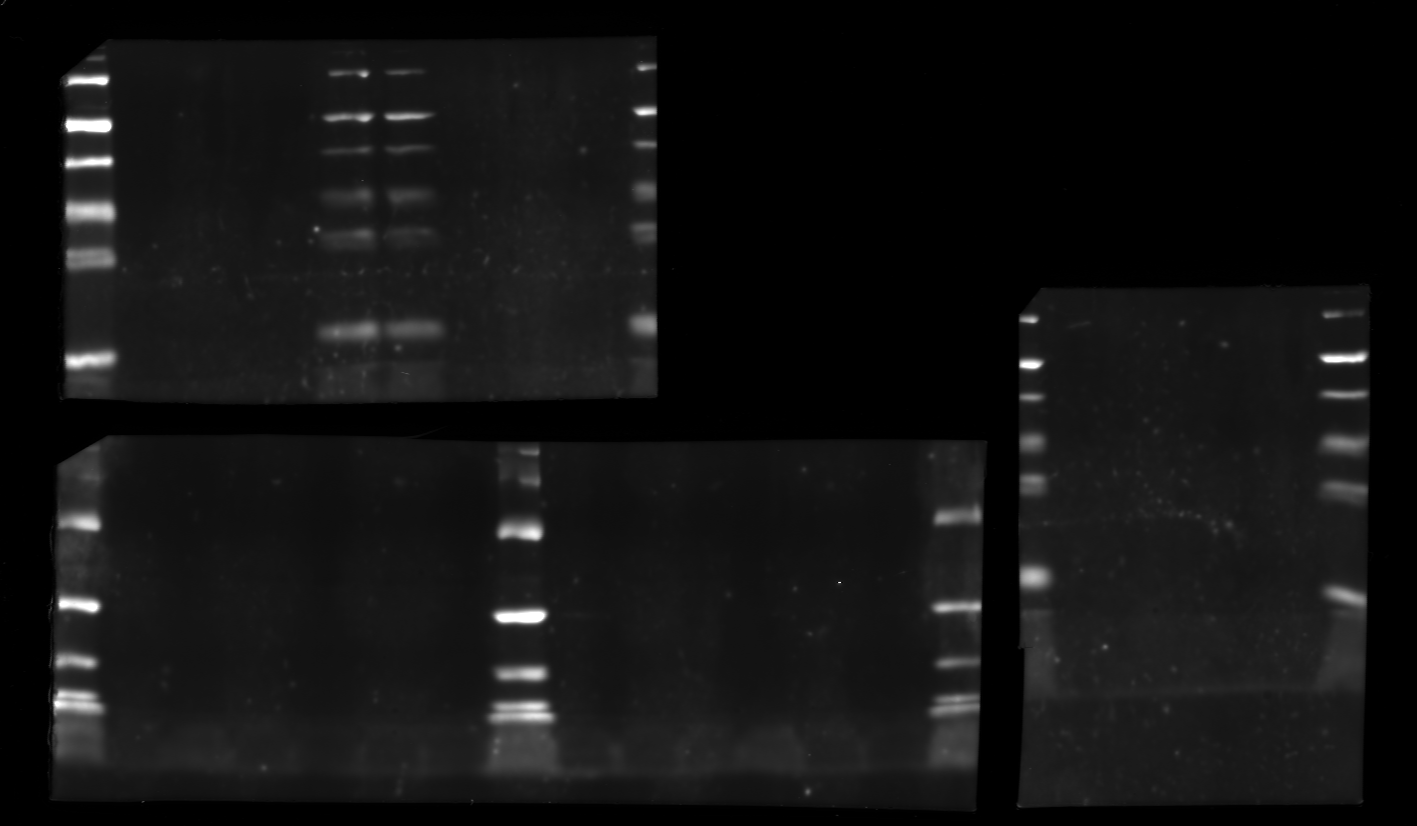

Supplement: Figure 4—source data 1. [file elife-68958-fig4-data1.zip › Figure 4ΓÇôsource data 1/Figure 4 full raw unedited blots files/original_files for B/2021-03-09-170016/700.TIF]

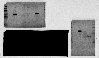

Supplement: Figure 4—source data 1. [file elife-68958-fig4-data1.zip › Figure 4ΓÇôsource data 1/Figure 4 full raw unedited blots files/original_files for B/2021-03-09-170016/2021-03-09-170016_IP a-HA RT3 a-Flag-MDA5 CARDs_TH.jpg]

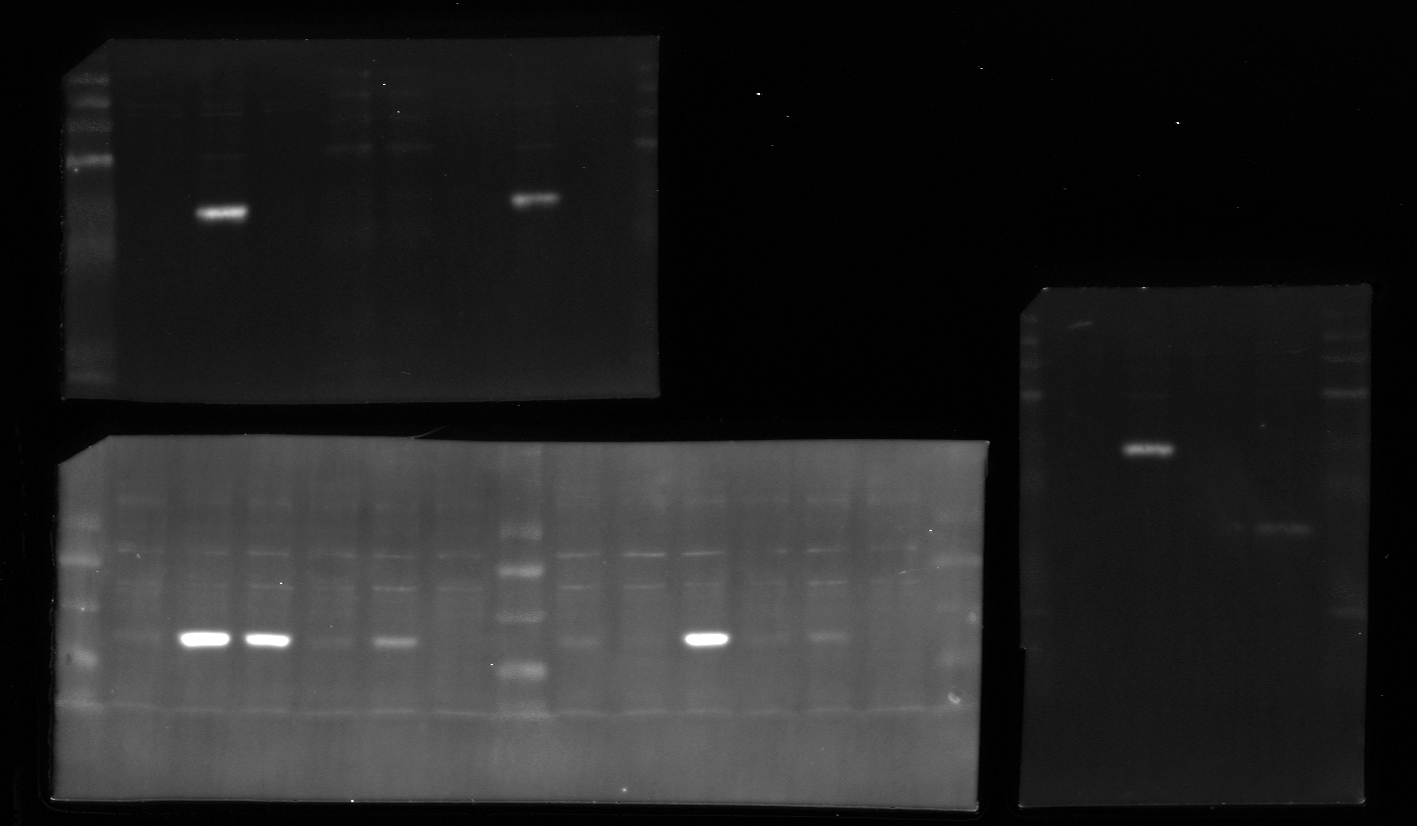

Supplement: Figure 4—source data 1. [file elife-68958-fig4-data1.zip › Figure 4ΓÇôsource data 1/Figure 4 full raw unedited blots files/original_files for B/2021-03-09-170016/800.TIF]

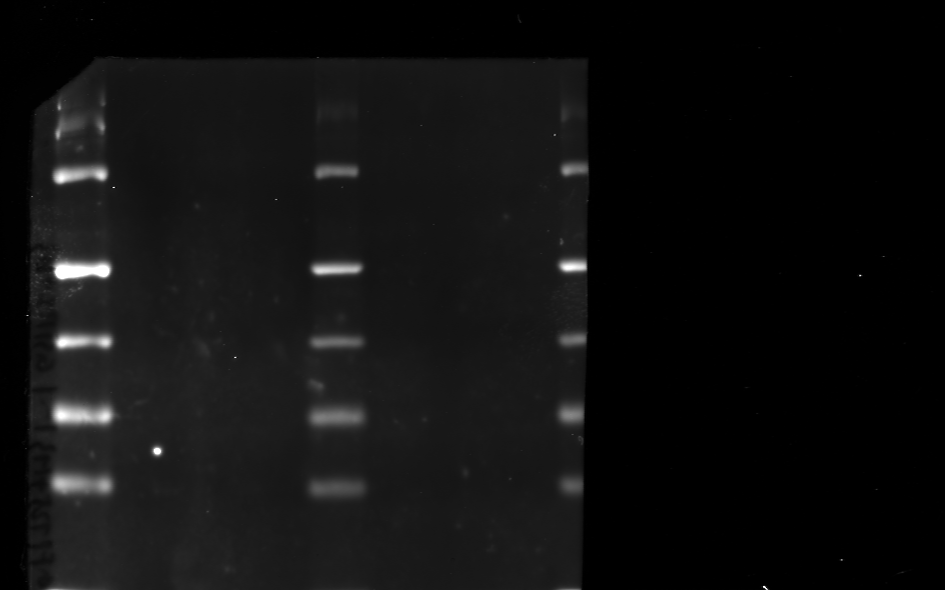

Supplement: Figure 4—source data 1. [file elife-68958-fig4-data1.zip › Figure 4ΓÇôsource data 1/Figure 4 full raw unedited blots files/original_files for B/2021-03-02-165804/700.TIF]

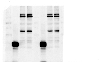

Supplement: Figure 4—source data 1. [file elife-68958-fig4-data1.zip › Figure 4ΓÇôsource data 1/Figure 4 full raw unedited blots files/original_files for B/2021-03-02-165804/2021-03-02-165804_a-GFP-TRIM25 GFP-RIG-I_TH.jpg]

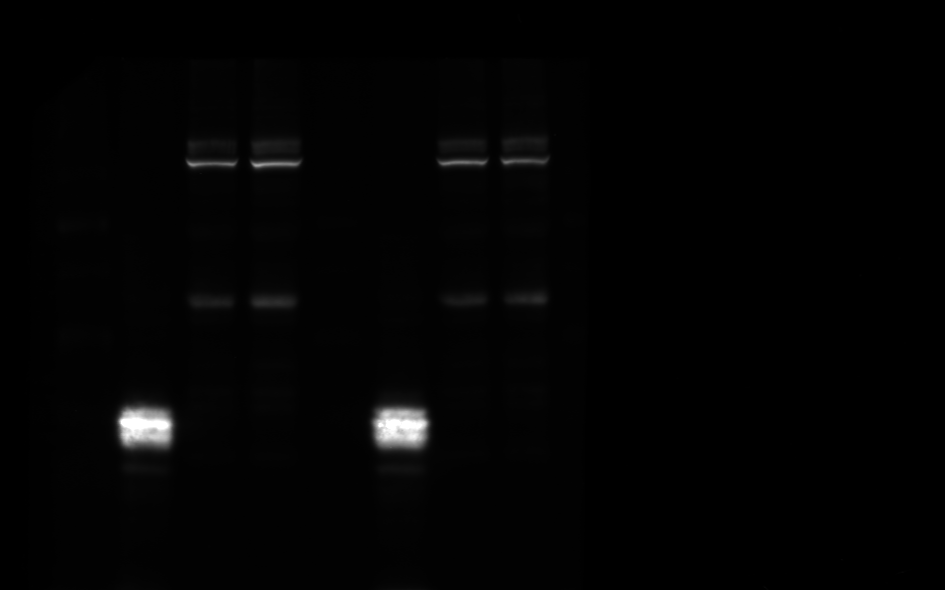

Supplement: Figure 4—source data 1. [file elife-68958-fig4-data1.zip › Figure 4ΓÇôsource data 1/Figure 4 full raw unedited blots files/original_files for B/2021-03-02-165804/800.TIF]
