# Supplementary figures and images for: RTN3 inhibits RIG-I-mediated antiviral responses by impairing TRIM25-mediated K63-linked polyubiquitination (part 2 of 3)
Source: eLife. 2021 Jul 27;10:e68958. doi: 10.7554/eLife.68958 (PMC8315805; doi:10.7554/eLife.68958)

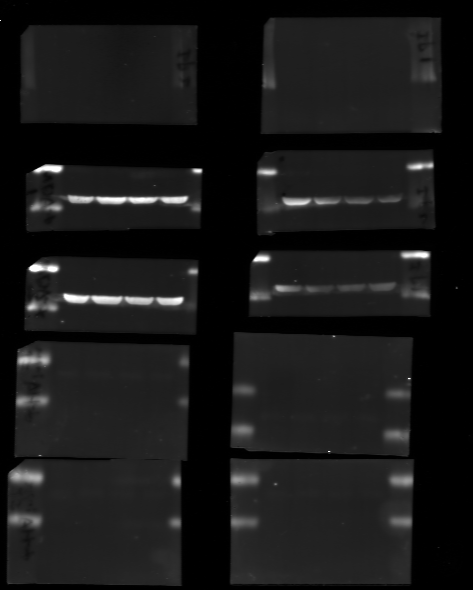

Supplement: Figure 4—source data 1. [file elife-68958-fig4-data1.zip › Figure 4ΓÇôsource data 1/Figure 4 full raw unedited blots files/original_files for G/2020-08-27-112705/700.TIF]

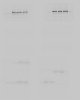

Supplement: Figure 4—source data 1. [file elife-68958-fig4-data1.zip › Figure 4ΓÇôsource data 1/Figure 4 full raw unedited blots files/original_files for G/2020-08-27-112705/2020-08-27-112705_a-IP RI input Actin IP HAR3 input R3_TH.jpg]

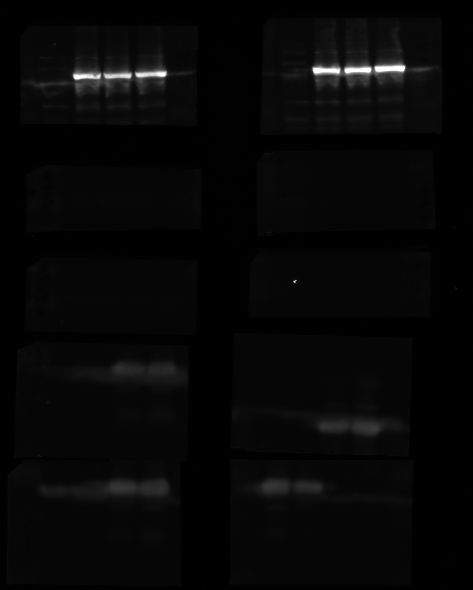

Supplement: Figure 4—source data 1. [file elife-68958-fig4-data1.zip › Figure 4ΓÇôsource data 1/Figure 4 full raw unedited blots files/original_files for G/2020-08-27-112705/800.TIF]

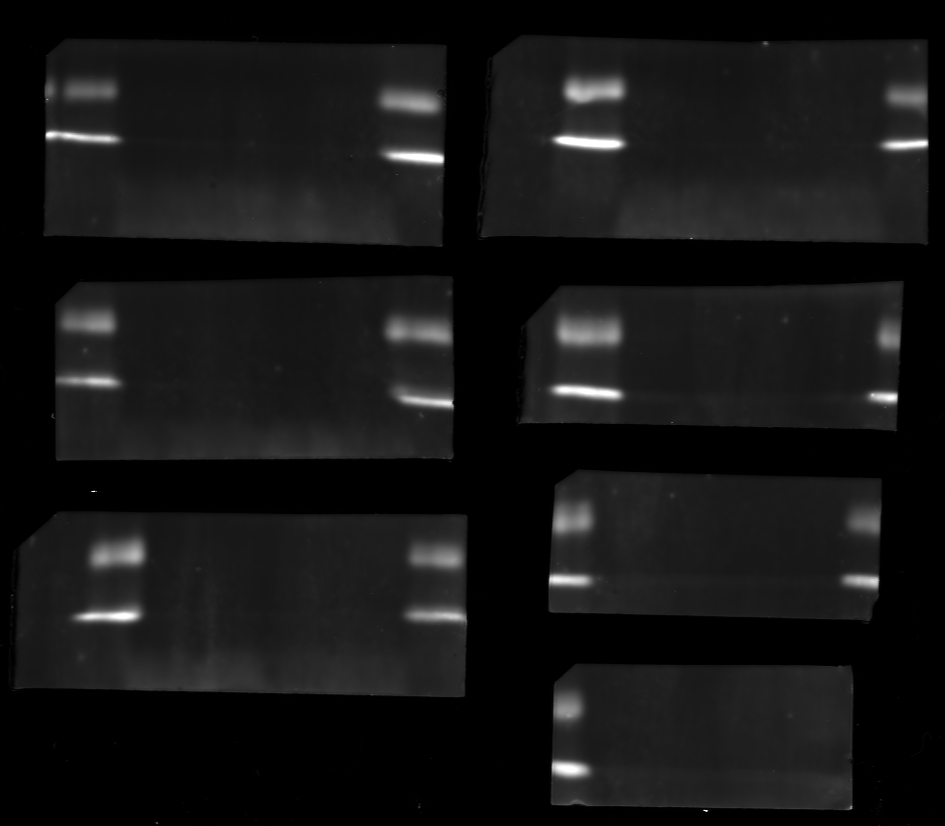

Supplement: Figure 4—source data 1. [file elife-68958-fig4-data1.zip › Figure 4ΓÇôsource data 1/Figure 4 full raw unedited blots files/original_files for G/2021-05-28-205853/700.TIF]

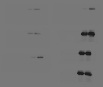

Supplement: Figure 4—source data 1. [file elife-68958-fig4-data1.zip › Figure 4ΓÇôsource data 1/Figure 4 full raw unedited blots files/original_files for G/2021-05-28-205853/2021-05-28-205853_RT3_TH.jpg]

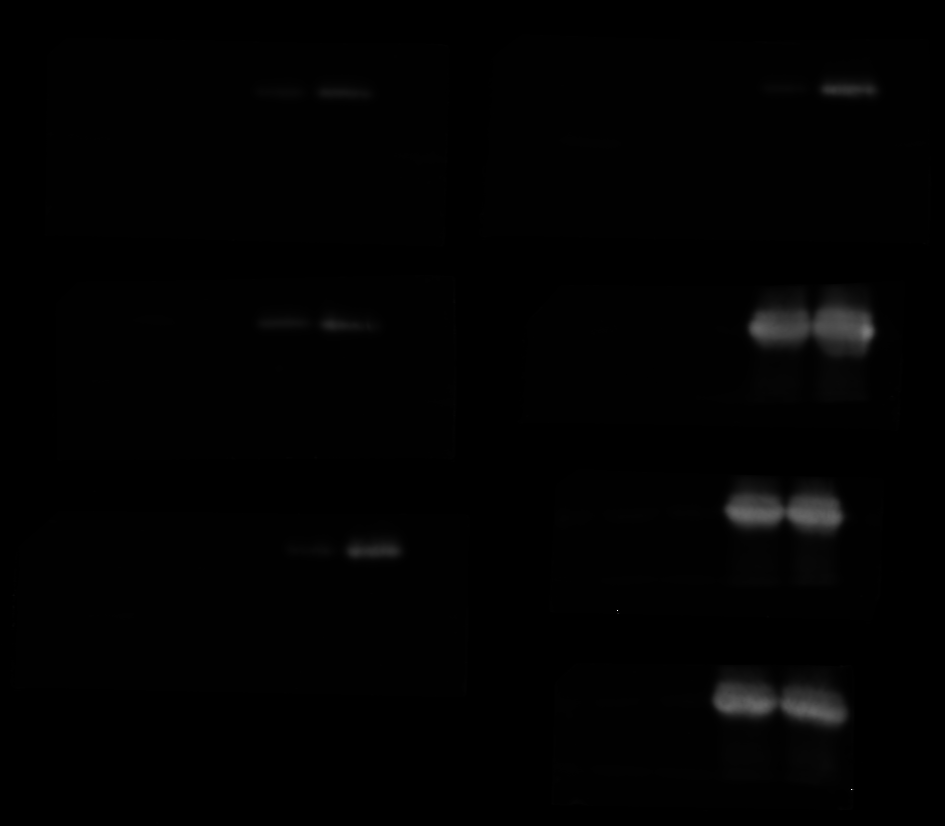

Supplement: Figure 4—source data 1. [file elife-68958-fig4-data1.zip › Figure 4ΓÇôsource data 1/Figure 4 full raw unedited blots files/original_files for G/2021-05-28-205853/800.TIF]

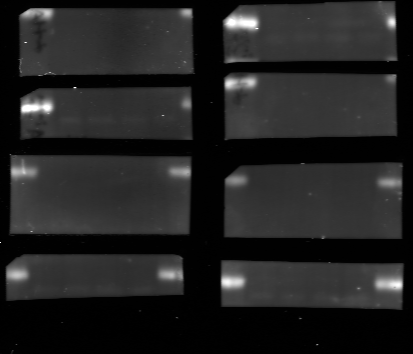

Supplement: Figure 4—source data 1. [file elife-68958-fig4-data1.zip › Figure 4ΓÇôsource data 1/Figure 4 full raw unedited blots files/original_files for G/2020-08-27-114303/700.TIF]

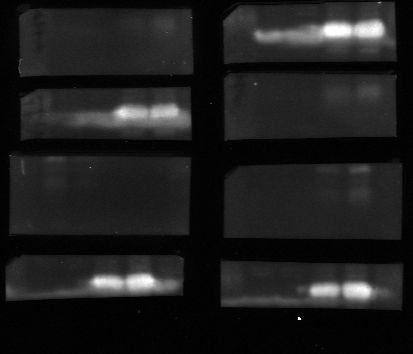

Supplement: Figure 4—source data 1. [file elife-68958-fig4-data1.zip › Figure 4ΓÇôsource data 1/Figure 4 full raw unedited blots files/original_files for G/2020-08-27-114303/800.TIF]

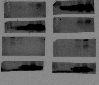

Supplement: Figure 4—source data 1. [file elife-68958-fig4-data1.zip › Figure 4ΓÇôsource data 1/Figure 4 full raw unedited blots files/original_files for G/2020-08-27-114303/2020-08-27-114303_a-HAR3_TH.jpg]

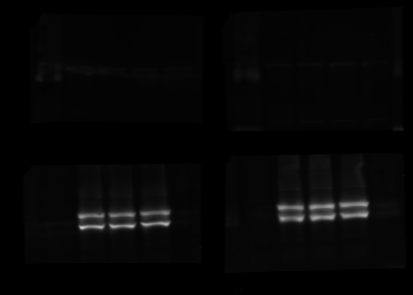

Supplement: Figure 4—source data 1. [file elife-68958-fig4-data1.zip › Figure 4ΓÇôsource data 1/Figure 4 full raw unedited blots files/original_files for G/2020-08-28-200329/700.TIF]

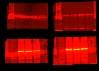

Supplement: Figure 4—source data 1. [file elife-68958-fig4-data1.zip › Figure 4ΓÇôsource data 1/Figure 4 full raw unedited blots files/original_files for G/2020-08-28-200329/2020-08-28-200329_a-RIGI IP GFP-T25_TH.jpg]

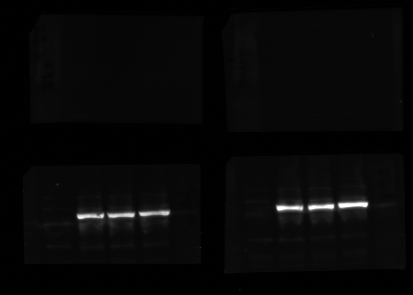

Supplement: Figure 4—source data 1. [file elife-68958-fig4-data1.zip › Figure 4ΓÇôsource data 1/Figure 4 full raw unedited blots files/original_files for G/2020-08-28-200329/800.TIF]

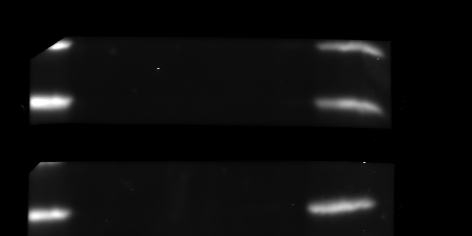

Supplement: Figure 4—source data 1. [file elife-68958-fig4-data1.zip › Figure 4ΓÇôsource data 1/Figure 4 full raw unedited blots files/original_files for H/2021-06-08-133148/700.TIF]

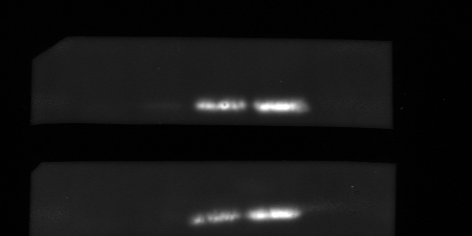

Supplement: Figure 4—source data 1. [file elife-68958-fig4-data1.zip › Figure 4ΓÇôsource data 1/Figure 4 full raw unedited blots files/original_files for H/2021-06-08-133148/800.TIF]

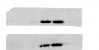

Supplement: Figure 4—source data 1. [file elife-68958-fig4-data1.zip › Figure 4ΓÇôsource data 1/Figure 4 full raw unedited blots files/original_files for H/2021-06-08-133148/2021-06-08-133148_Flag RT3_TH.jpg]

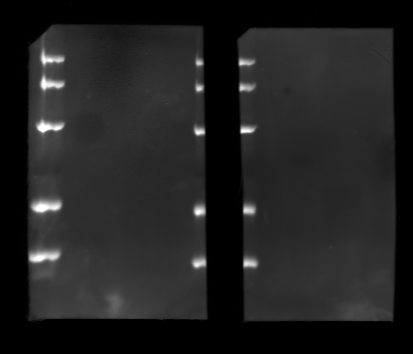

Supplement: Figure 4—source data 1. [file elife-68958-fig4-data1.zip › Figure 4ΓÇôsource data 1/Figure 4 full raw unedited blots files/original_files for H/2021-06-11-132354/700.TIF]

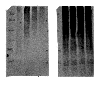

Supplement: Figure 4—source data 1. [file elife-68958-fig4-data1.zip › Figure 4ΓÇôsource data 1/Figure 4 full raw unedited blots files/original_files for H/2021-06-11-132354/2021-06-11-132354_UB-K63 WT 293T_TH.jpg]

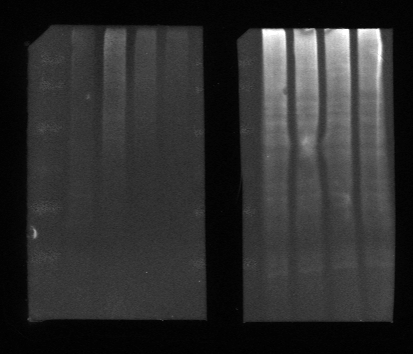

Supplement: Figure 4—source data 1. [file elife-68958-fig4-data1.zip › Figure 4ΓÇôsource data 1/Figure 4 full raw unedited blots files/original_files for H/2021-06-11-132354/800.TIF]

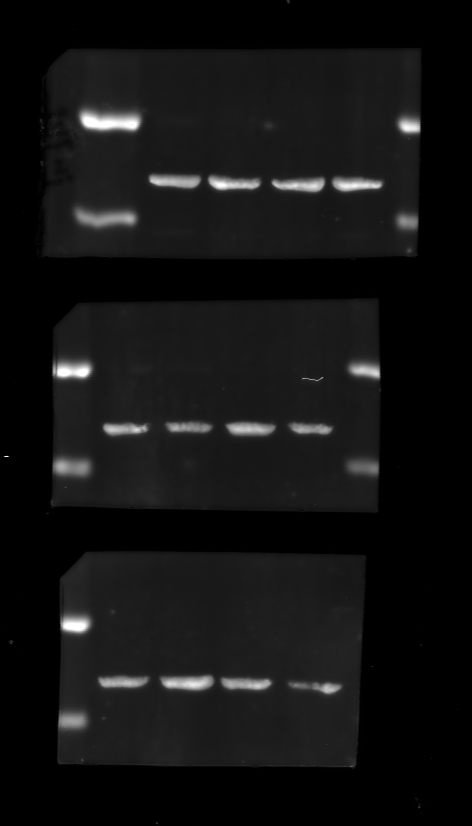

Supplement: Figure 4—source data 1. [file elife-68958-fig4-data1.zip › Figure 4ΓÇôsource data 1/Figure 4 full raw unedited blots files/original_files for H/2021-05-28-205034/700.TIF]

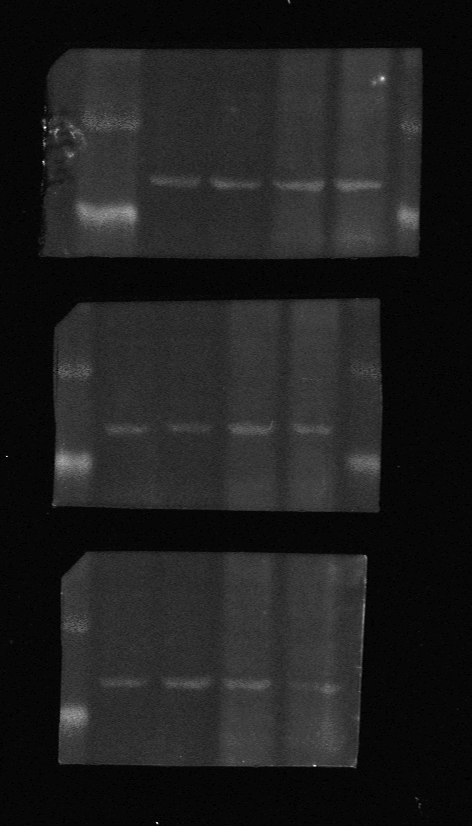

Supplement: Figure 4—source data 1. [file elife-68958-fig4-data1.zip › Figure 4ΓÇôsource data 1/Figure 4 full raw unedited blots files/original_files for H/2021-05-28-205034/800.TIF]

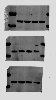

Supplement: Figure 4—source data 1. [file elife-68958-fig4-data1.zip › Figure 4ΓÇôsource data 1/Figure 4 full raw unedited blots files/original_files for H/2021-05-28-205034/2021-05-28-205034_actin_TH.jpg]

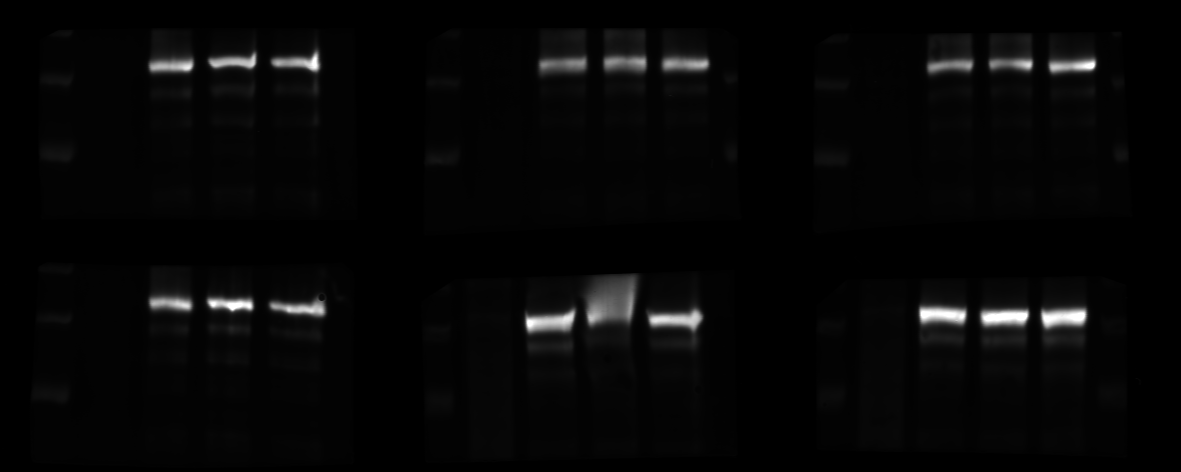

Supplement: Figure 4—source data 1. [file elife-68958-fig4-data1.zip › Figure 4ΓÇôsource data 1/Figure 4 full raw unedited blots files/original_files for H/2021-05-21-214618/700.TIF]

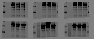

Supplement: Figure 4—source data 1. [file elife-68958-fig4-data1.zip › Figure 4ΓÇôsource data 1/Figure 4 full raw unedited blots files/original_files for H/2021-05-21-214618/2021-05-21-214618_GFP RIG-I_TH.jpg]

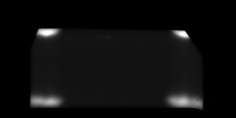

Supplement: Figure 4—source data 1. [file elife-68958-fig4-data1.zip › Figure 4ΓÇôsource data 1/Figure 4 full raw unedited blots files/original_files for F/2021-05-26-210259/700.TIF]

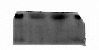

Supplement: Figure 4—source data 1. [file elife-68958-fig4-data1.zip › Figure 4ΓÇôsource data 1/Figure 4 full raw unedited blots files/original_files for F/2021-05-26-210259/2021-05-26-210259_a-RT3_TH.jpg]

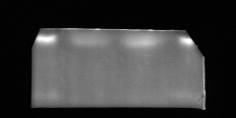

Supplement: Figure 4—source data 1. [file elife-68958-fig4-data1.zip › Figure 4ΓÇôsource data 1/Figure 4 full raw unedited blots files/original_files for F/2021-05-26-210259/800.TIF]

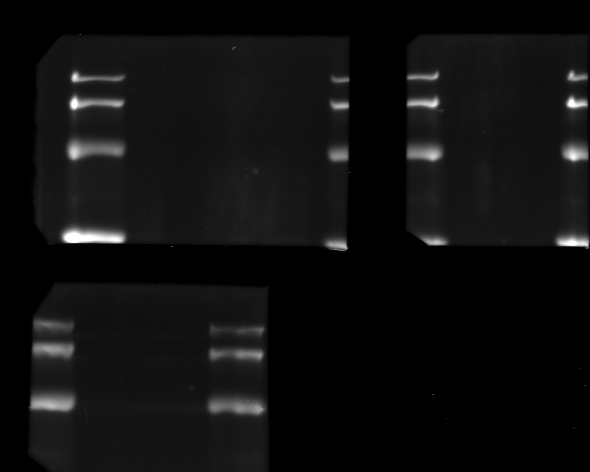

Supplement: Figure 4—source data 1. [file elife-68958-fig4-data1.zip › Figure 4ΓÇôsource data 1/Figure 4 full raw unedited blots files/original_files for F/2021-05-26-212123/700.TIF]

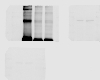

Supplement: Figure 4—source data 1. [file elife-68958-fig4-data1.zip › Figure 4ΓÇôsource data 1/Figure 4 full raw unedited blots files/original_files for F/2021-05-26-212123/2021-05-26-212123_a-RIG-I IP3 INPUT2_TH.jpg]

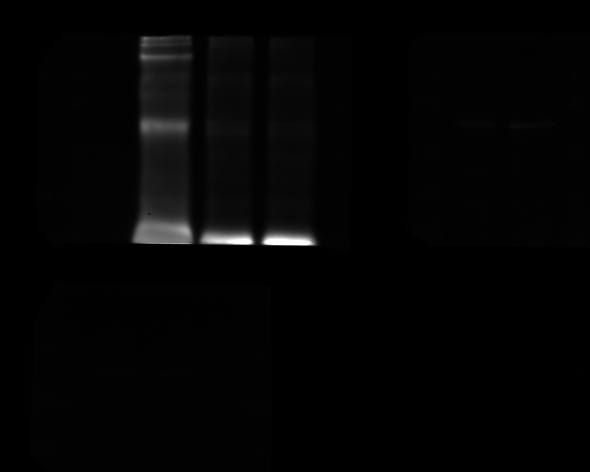

Supplement: Figure 4—source data 1. [file elife-68958-fig4-data1.zip › Figure 4ΓÇôsource data 1/Figure 4 full raw unedited blots files/original_files for F/2021-05-26-212123/800.TIF]

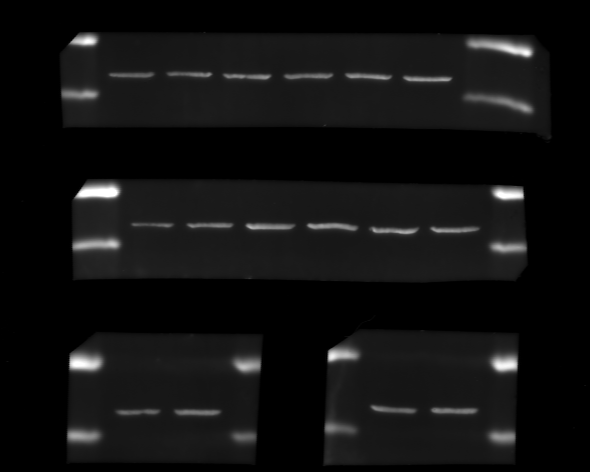

Supplement: Figure 4—source data 1. [file elife-68958-fig4-data1.zip › Figure 4ΓÇôsource data 1/Figure 4 full raw unedited blots files/original_files for F/2021-05-26-203425/700.TIF]

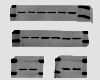

Supplement: Figure 4—source data 1. [file elife-68958-fig4-data1.zip › Figure 4ΓÇôsource data 1/Figure 4 full raw unedited blots files/original_files for F/2021-05-26-203425/2021-05-26-203425_a-Actin_TH.jpg]

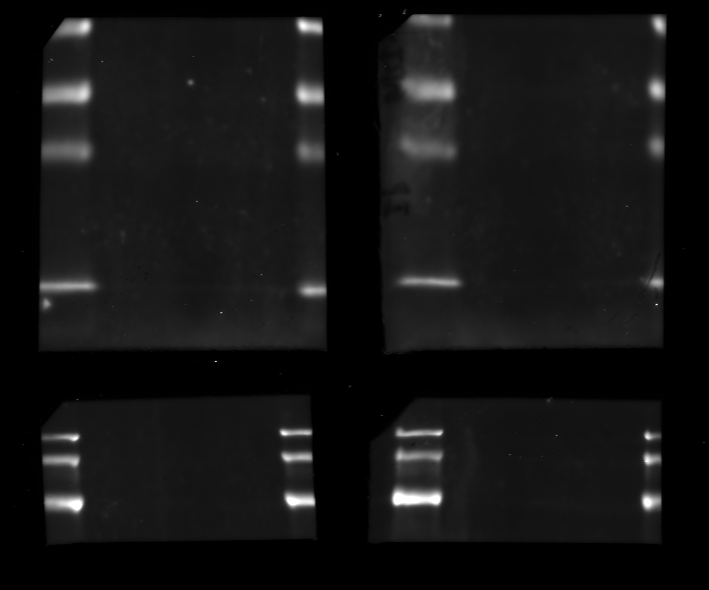

Supplement: Figure 4—source data 1. [file elife-68958-fig4-data1.zip › Figure 4ΓÇôsource data 1/Figure 4 full raw unedited blots files/original_files for F/2021-05-31-165733/700.TIF]

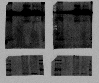

Supplement: Figure 4—source data 1. [file elife-68958-fig4-data1.zip › Figure 4ΓÇôsource data 1/Figure 4 full raw unedited blots files/original_files for F/2021-05-31-165733/2021-05-31-165733_endougenous IP RT3 RIG-I RT3_TH.jpg]

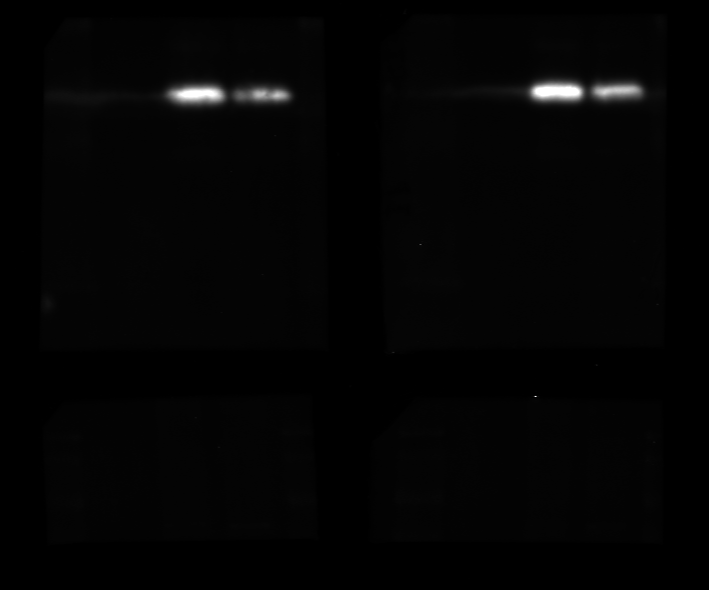

Supplement: Figure 4—source data 1. [file elife-68958-fig4-data1.zip › Figure 4ΓÇôsource data 1/Figure 4 full raw unedited blots files/original_files for F/2021-05-31-165733/800.TIF]

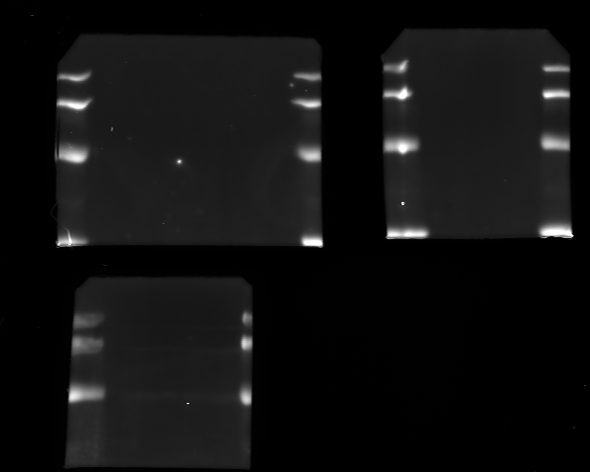

Supplement: Figure 4—source data 1. [file elife-68958-fig4-data1.zip › Figure 4ΓÇôsource data 1/Figure 4 full raw unedited blots files/original_files for F/2021-05-26-204815/700.TIF]

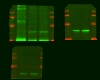

Supplement: Figure 4—source data 1. [file elife-68958-fig4-data1.zip › Figure 4ΓÇôsource data 1/Figure 4 full raw unedited blots files/original_files for F/2021-05-26-204815/2021-05-26-204815_a-T25 IP3 INPUT2_TH.jpg]

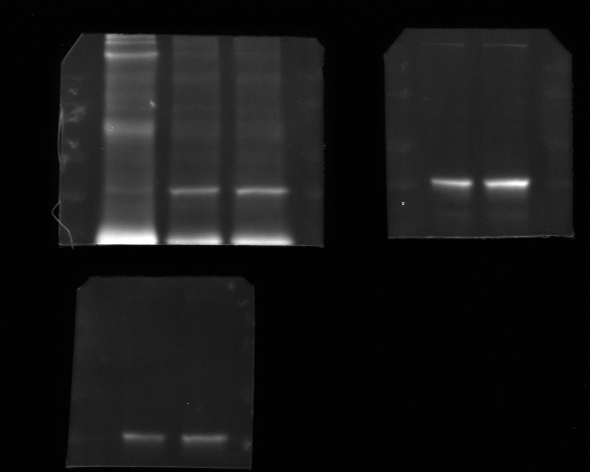

Supplement: Figure 4—source data 1. [file elife-68958-fig4-data1.zip › Figure 4ΓÇôsource data 1/Figure 4 full raw unedited blots files/original_files for F/2021-05-26-204815/800.TIF]

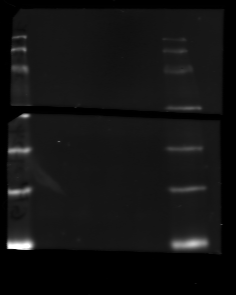

Supplement: Figure 4—source data 1. [file elife-68958-fig4-data1.zip › Figure 4ΓÇôsource data 1/Figure 4 full raw unedited blots files/original_files for A/2020-07-05-223459/700.TIF]

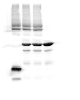

Supplement: Figure 4—source data 1. [file elife-68958-fig4-data1.zip › Figure 4ΓÇôsource data 1/Figure 4 full raw unedited blots files/original_files for A/2020-07-05-223459/2020-07-05-223459_a-Flag-T25 GFP-RT3 input_TH.jpg]

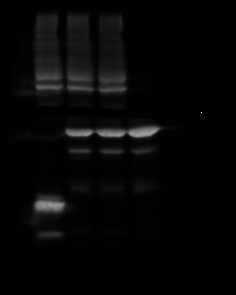

Supplement: Figure 4—source data 1. [file elife-68958-fig4-data1.zip › Figure 4ΓÇôsource data 1/Figure 4 full raw unedited blots files/original_files for A/2020-07-05-223459/800.TIF]

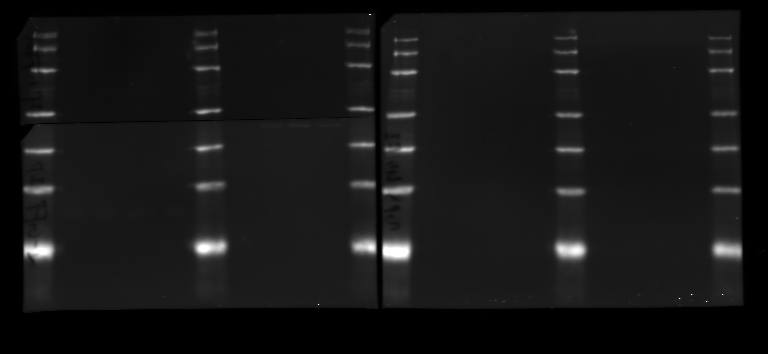

Supplement: Figure 4—source data 1. [file elife-68958-fig4-data1.zip › Figure 4ΓÇôsource data 1/Figure 4 full raw unedited blots files/original_files for A/2020-06-16-140826/700.TIF]

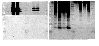

Supplement: Figure 4—source data 1. [file elife-68958-fig4-data1.zip › Figure 4ΓÇôsource data 1/Figure 4 full raw unedited blots files/original_files for A/2020-06-16-140826/2020-06-16-140826_a-Flag-T25 GFP-RT3 input IP a-HA-Ub input IP RIGI_TH.jpg]

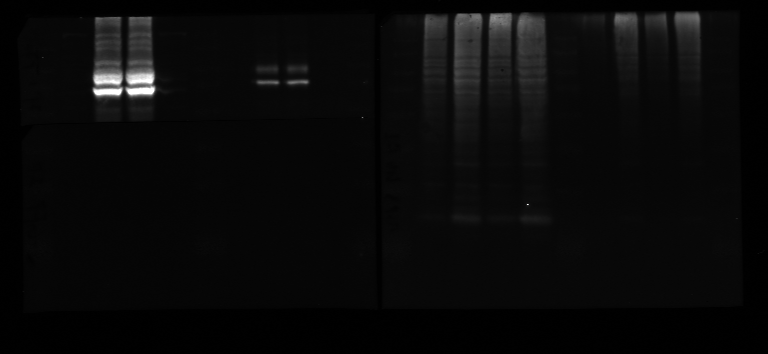

Supplement: Figure 4—source data 1. [file elife-68958-fig4-data1.zip › Figure 4ΓÇôsource data 1/Figure 4 full raw unedited blots files/original_files for A/2020-06-16-140826/800.TIF]

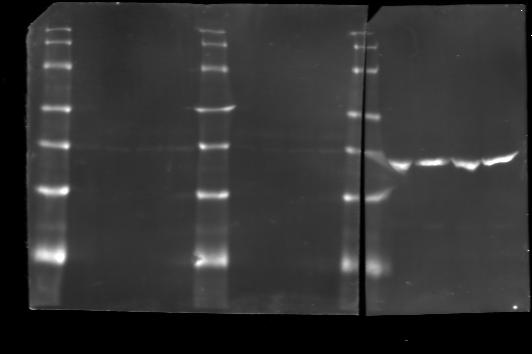

Supplement: Figure 4—source data 1. [file elife-68958-fig4-data1.zip › Figure 4ΓÇôsource data 1/Figure 4 full raw unedited blots files/original_files for A/2020-01-11-165202/700.TIF]

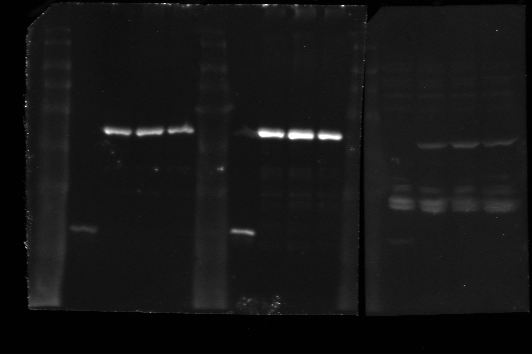

Supplement: Figure 4—source data 1. [file elife-68958-fig4-data1.zip › Figure 4ΓÇôsource data 1/Figure 4 full raw unedited blots files/original_files for A/2020-01-11-165202/800.TIF]

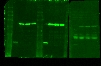

Supplement: Figure 4—source data 1. [file elife-68958-fig4-data1.zip › Figure 4ΓÇôsource data 1/Figure 4 full raw unedited blots files/original_files for A/2020-01-11-165202/2020-01-11-165202_a-GST Actin beads elution_TH.jpg]

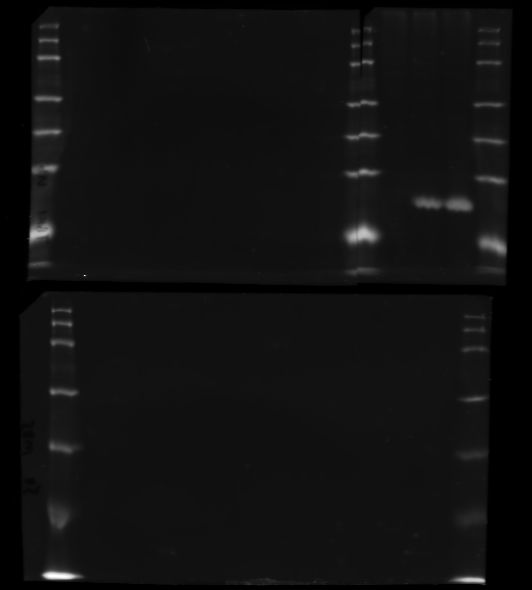

Supplement: Figure 4—figure supplement 1—source data 1. [file elife-68958-fig4-figsupp1-data1.zip › Figure 4-figure supplement 1ΓÇôsource data 1/Figure 4-figure supplement 1 full raw unedited blots files/original_files for D/2020-05-23-132131/700.TIF]

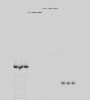

Supplement: Figure 4—figure supplement 1—source data 1. [file elife-68958-fig4-figsupp1-data1.zip › Figure 4-figure supplement 1ΓÇôsource data 1/Figure 4-figure supplement 1 full raw unedited blots files/original_files for D/2020-05-23-132131/2020-05-23-132131_a-Flag_TH.jpg]

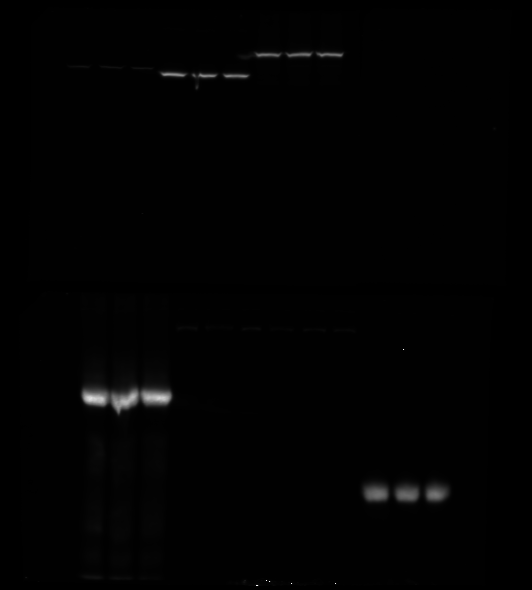

Supplement: Figure 4—figure supplement 1—source data 1. [file elife-68958-fig4-figsupp1-data1.zip › Figure 4-figure supplement 1ΓÇôsource data 1/Figure 4-figure supplement 1 full raw unedited blots files/original_files for D/2020-05-23-132131/800.TIF]

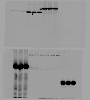

Supplement: Figure 4—figure supplement 1—source data 1. [file elife-68958-fig4-figsupp1-data1.zip › Figure 4-figure supplement 1ΓÇôsource data 1/Figure 4-figure supplement 1 full raw unedited blots files/original_files for D/2020-05-23-132131/2020-05-23-132131_a-Flag TBK1 IKKI RIGI IRF35D 1 2 MDA5 MDA5-CARD_TH.jpg]

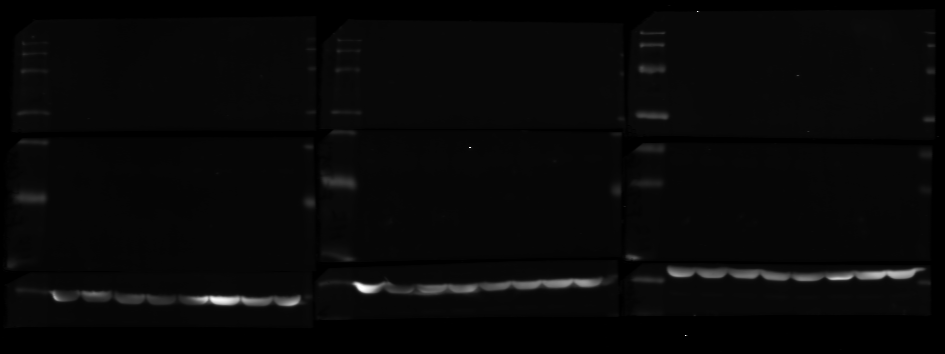

Supplement: Figure 4—figure supplement 1—source data 1. [file elife-68958-fig4-figsupp1-data1.zip › Figure 4-figure supplement 1ΓÇôsource data 1/Figure 4-figure supplement 1 full raw unedited blots files/original_files for D/2020-07-14-203916/700.TIF]

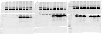

Supplement: Figure 4—figure supplement 1—source data 1. [file elife-68958-fig4-figsupp1-data1.zip › Figure 4-figure supplement 1ΓÇôsource data 1/Figure 4-figure supplement 1 full raw unedited blots files/original_files for D/2020-07-14-203916/2020-07-14-203916_a-F-T25 HA-RT3_TH.jpg]

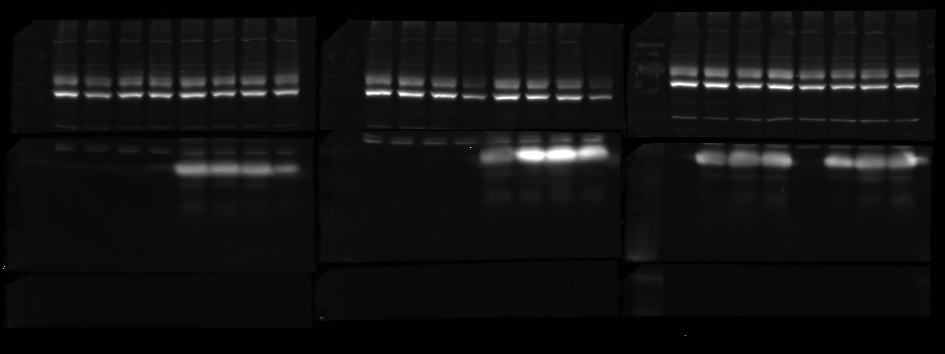

Supplement: Figure 4—figure supplement 1—source data 1. [file elife-68958-fig4-figsupp1-data1.zip › Figure 4-figure supplement 1ΓÇôsource data 1/Figure 4-figure supplement 1 full raw unedited blots files/original_files for D/2020-07-14-203916/800.TIF]

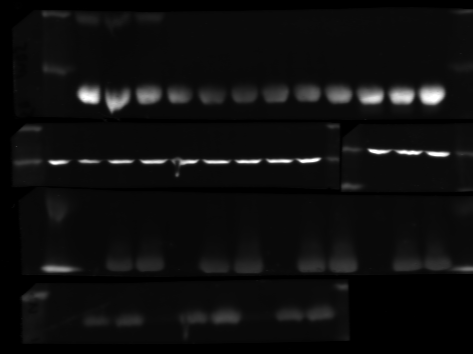

Supplement: Figure 4—figure supplement 1—source data 1. [file elife-68958-fig4-figsupp1-data1.zip › Figure 4-figure supplement 1ΓÇôsource data 1/Figure 4-figure supplement 1 full raw unedited blots files/original_files for D/2020-05-25-110537/700.TIF]

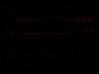

Supplement: Figure 4—figure supplement 1—source data 1. [file elife-68958-fig4-figsupp1-data1.zip › Figure 4-figure supplement 1ΓÇôsource data 1/Figure 4-figure supplement 1 full raw unedited blots files/original_files for D/2020-05-25-110537/2020-05-25-110537_a-Atin HA-RT3_TH.jpg]

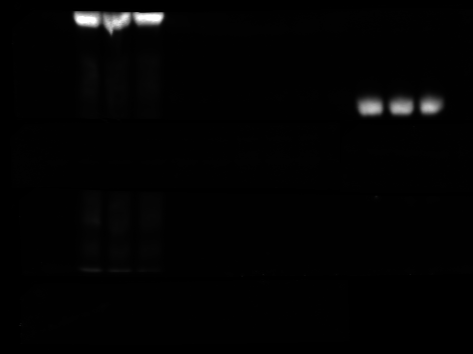

Supplement: Figure 4—figure supplement 1—source data 1. [file elife-68958-fig4-figsupp1-data1.zip › Figure 4-figure supplement 1ΓÇôsource data 1/Figure 4-figure supplement 1 full raw unedited blots files/original_files for D/2020-05-25-110537/800.TIF]

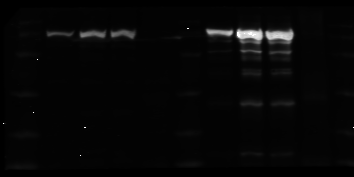

Supplement: Figure 4—figure supplement 1—source data 1. [file elife-68958-fig4-figsupp1-data1.zip › Figure 4-figure supplement 1ΓÇôsource data 1/Figure 4-figure supplement 1 full raw unedited blots files/original_files for C/2020-06-29-114319/700.TIF]

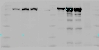

Supplement: Figure 4—figure supplement 1—source data 1. [file elife-68958-fig4-figsupp1-data1.zip › Figure 4-figure supplement 1ΓÇôsource data 1/Figure 4-figure supplement 1 full raw unedited blots files/original_files for C/2020-06-29-114319/2020-06-29-114319_a-GFP RIGI_TH.jpg]

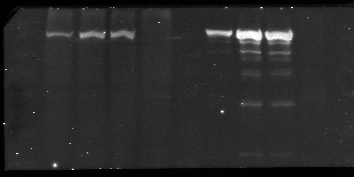

Supplement: Figure 4—figure supplement 1—source data 1. [file elife-68958-fig4-figsupp1-data1.zip › Figure 4-figure supplement 1ΓÇôsource data 1/Figure 4-figure supplement 1 full raw unedited blots files/original_files for C/2020-06-29-114319/800.TIF]

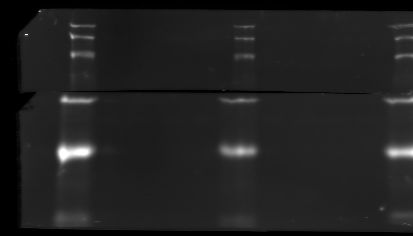

Supplement: Figure 4—figure supplement 1—source data 1. [file elife-68958-fig4-figsupp1-data1.zip › Figure 4-figure supplement 1ΓÇôsource data 1/Figure 4-figure supplement 1 full raw unedited blots files/original_files for C/2020-06-30-225436/700.TIF]

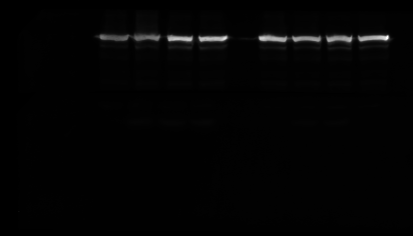

Supplement: Figure 4—figure supplement 1—source data 1. [file elife-68958-fig4-figsupp1-data1.zip › Figure 4-figure supplement 1ΓÇôsource data 1/Figure 4-figure supplement 1 full raw unedited blots files/original_files for C/2020-06-30-225436/800.TIF]

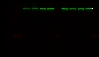

Supplement: Figure 4—figure supplement 1—source data 1. [file elife-68958-fig4-figsupp1-data1.zip › Figure 4-figure supplement 1ΓÇôsource data 1/Figure 4-figure supplement 1 full raw unedited blots files/original_files for C/2020-06-30-225436/2020-06-30-225436_a-HA RT3 input IP_TH.jpg]

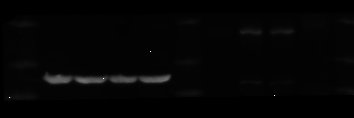

Supplement: Figure 4—figure supplement 1—source data 1. [file elife-68958-fig4-figsupp1-data1.zip › Figure 4-figure supplement 1ΓÇôsource data 1/Figure 4-figure supplement 1 full raw unedited blots files/original_files for C/2020-06-30-230025/700.TIF]

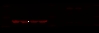

Supplement: Figure 4—figure supplement 1—source data 1. [file elife-68958-fig4-figsupp1-data1.zip › Figure 4-figure supplement 1ΓÇôsource data 1/Figure 4-figure supplement 1 full raw unedited blots files/original_files for C/2020-06-30-230025/2020-06-30-230025_a-Actin_TH.jpg]

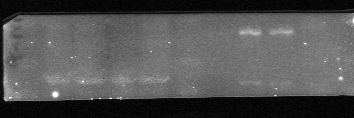

Supplement: Figure 4—figure supplement 1—source data 1. [file elife-68958-fig4-figsupp1-data1.zip › Figure 4-figure supplement 1ΓÇôsource data 1/Figure 4-figure supplement 1 full raw unedited blots files/original_files for C/2020-06-30-230025/800.TIF]

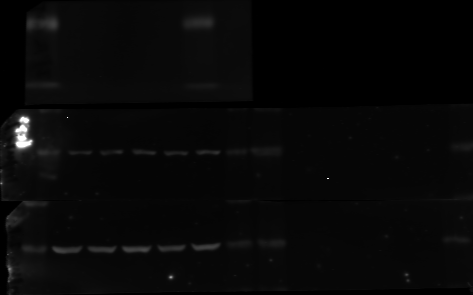

Supplement: Figure 4—figure supplement 1—source data 1. [file elife-68958-fig4-figsupp1-data1.zip › Figure 4-figure supplement 1ΓÇôsource data 1/Figure 4-figure supplement 1 full raw unedited blots files/original_files for B/2020-07-11-231001/700.TIF]

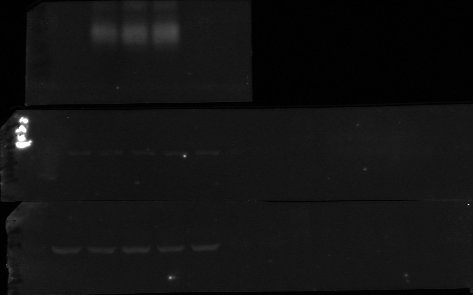

Supplement: Figure 4—figure supplement 1—source data 1. [file elife-68958-fig4-figsupp1-data1.zip › Figure 4-figure supplement 1ΓÇôsource data 1/Figure 4-figure supplement 1 full raw unedited blots files/original_files for B/2020-07-11-231001/800.TIF]

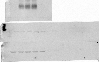

Supplement: Figure 4—figure supplement 1—source data 1. [file elife-68958-fig4-figsupp1-data1.zip › Figure 4-figure supplement 1ΓÇôsource data 1/Figure 4-figure supplement 1 full raw unedited blots files/original_files for B/2020-07-11-231001/2020-07-11-231001_a-RT3 Actin_TH.jpg]

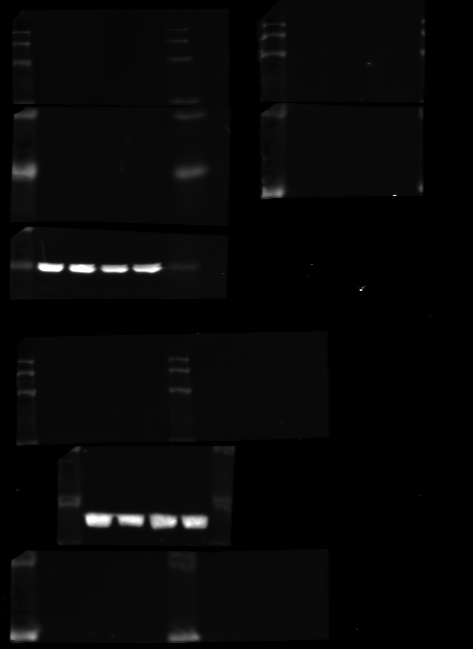

Supplement: Figure 4—figure supplement 1—source data 1. [file elife-68958-fig4-figsupp1-data1.zip › Figure 4-figure supplement 1ΓÇôsource data 1/Figure 4-figure supplement 1 full raw unedited blots files/original_files for B/2020-07-05-230812/700.TIF]

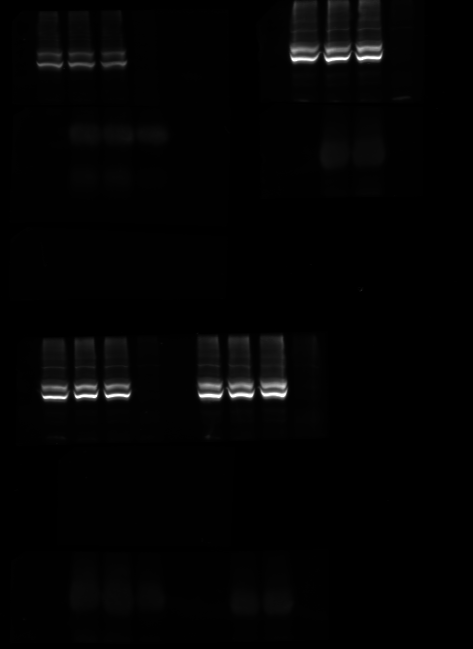

Supplement: Figure 4—figure supplement 1—source data 1. [file elife-68958-fig4-figsupp1-data1.zip › Figure 4-figure supplement 1ΓÇôsource data 1/Figure 4-figure supplement 1 full raw unedited blots files/original_files for B/2020-07-05-230812/800.TIF]

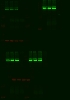

Supplement: Figure 4—figure supplement 1—source data 1. [file elife-68958-fig4-figsupp1-data1.zip › Figure 4-figure supplement 1ΓÇôsource data 1/Figure 4-figure supplement 1 full raw unedited blots files/original_files for B/2020-07-05-230812/2020-07-05-230812_a-GFP-T25 HA-RT3 input IP_TH.jpg]

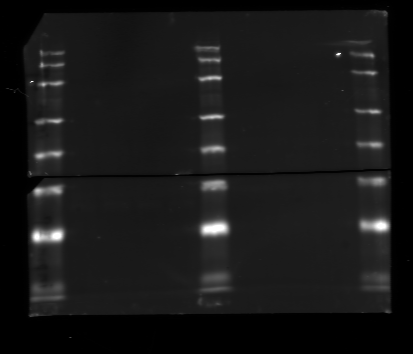

Supplement: Figure 4—figure supplement 1—source data 1. [file elife-68958-fig4-figsupp1-data1.zip › Figure 4-figure supplement 1ΓÇôsource data 1/Figure 4-figure supplement 1 full raw unedited blots files/original_files for B/2020-06-11-223226/700.TIF]

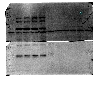

Supplement: Figure 4—figure supplement 1—source data 1. [file elife-68958-fig4-figsupp1-data1.zip › Figure 4-figure supplement 1ΓÇôsource data 1/Figure 4-figure supplement 1 full raw unedited blots files/original_files for B/2020-06-11-223226/2020-06-11-223226_a-RIGI RT3 input IP_TH.jpg]

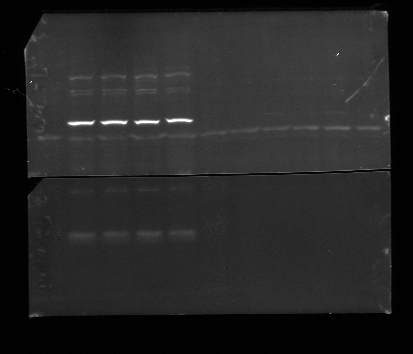

Supplement: Figure 4—figure supplement 1—source data 1. [file elife-68958-fig4-figsupp1-data1.zip › Figure 4-figure supplement 1ΓÇôsource data 1/Figure 4-figure supplement 1 full raw unedited blots files/original_files for B/2020-06-11-223226/800.TIF]

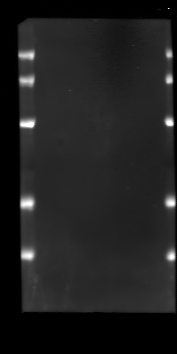

Supplement: Figure 4—figure supplement 1—source data 1. [file elife-68958-fig4-figsupp1-data1.zip › Figure 4-figure supplement 1ΓÇôsource data 1/Figure 4-figure supplement 1 full raw unedited blots files/original_files for E/2021-06-11-133008/700.TIF]

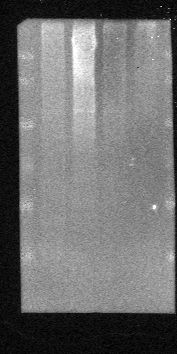

Supplement: Figure 4—figure supplement 1—source data 1. [file elife-68958-fig4-figsupp1-data1.zip › Figure 4-figure supplement 1ΓÇôsource data 1/Figure 4-figure supplement 1 full raw unedited blots files/original_files for E/2021-06-11-133008/800.TIF]

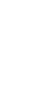

Supplement: Figure 4—figure supplement 1—source data 1. [file elife-68958-fig4-figsupp1-data1.zip › Figure 4-figure supplement 1ΓÇôsource data 1/Figure 4-figure supplement 1 full raw unedited blots files/original_files for E/2021-06-11-133008/2021-06-11-133008_UB-K63 WT 293T 2_TH.jpg]

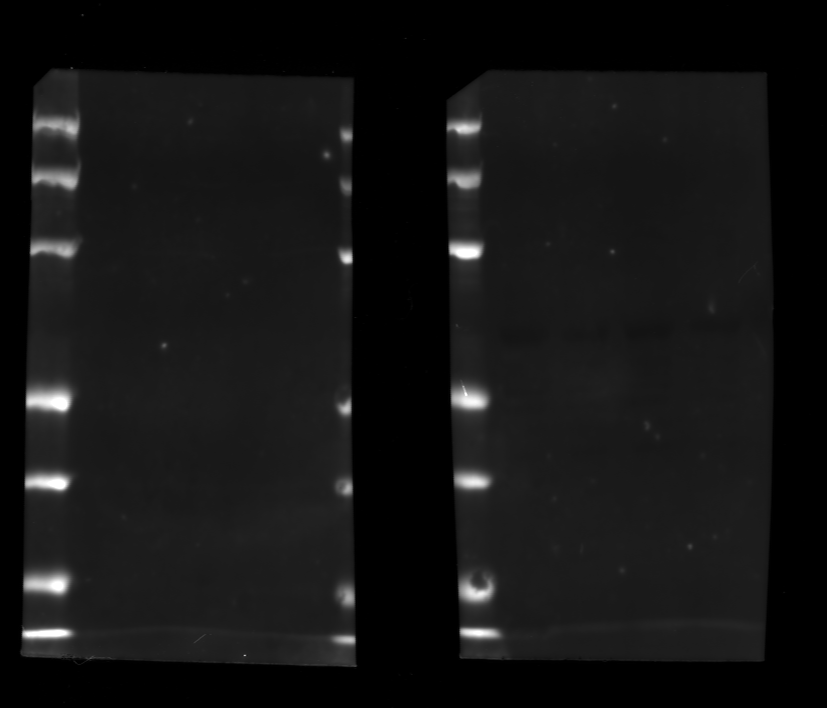

Supplement: Figure 4—figure supplement 1—source data 1. [file elife-68958-fig4-figsupp1-data1.zip › Figure 4-figure supplement 1ΓÇôsource data 1/Figure 4-figure supplement 1 full raw unedited blots files/original_files for E/2021-06-13-190514/700.TIF]

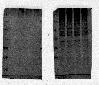

Supplement: Figure 4—figure supplement 1—source data 1. [file elife-68958-fig4-figsupp1-data1.zip › Figure 4-figure supplement 1ΓÇôsource data 1/Figure 4-figure supplement 1 full raw unedited blots files/original_files for E/2021-06-13-190514/2021-06-13-190514_a-Ub-K63_TH.jpg]

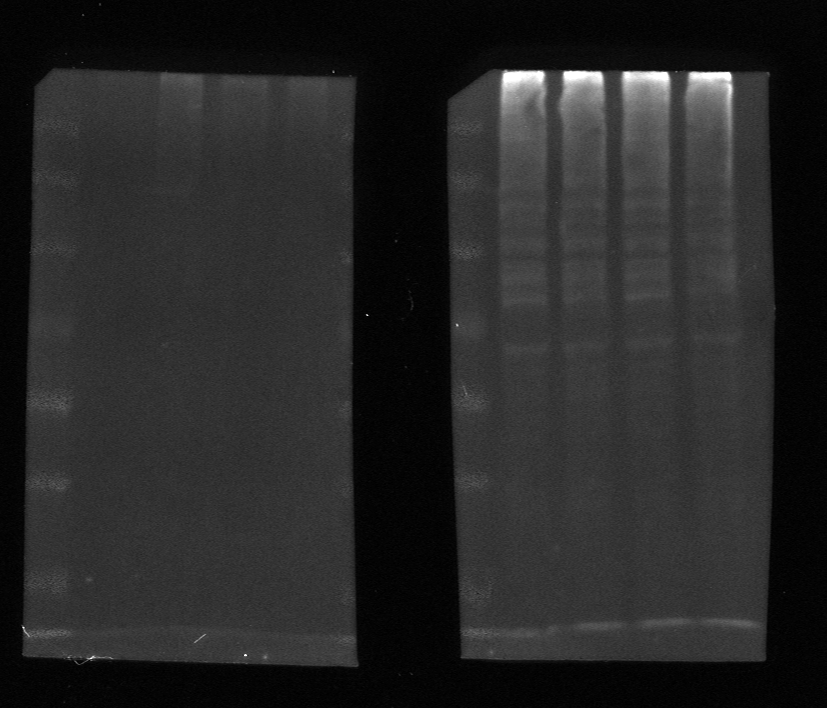

Supplement: Figure 4—figure supplement 1—source data 1. [file elife-68958-fig4-figsupp1-data1.zip › Figure 4-figure supplement 1ΓÇôsource data 1/Figure 4-figure supplement 1 full raw unedited blots files/original_files for E/2021-06-13-190514/800.TIF]

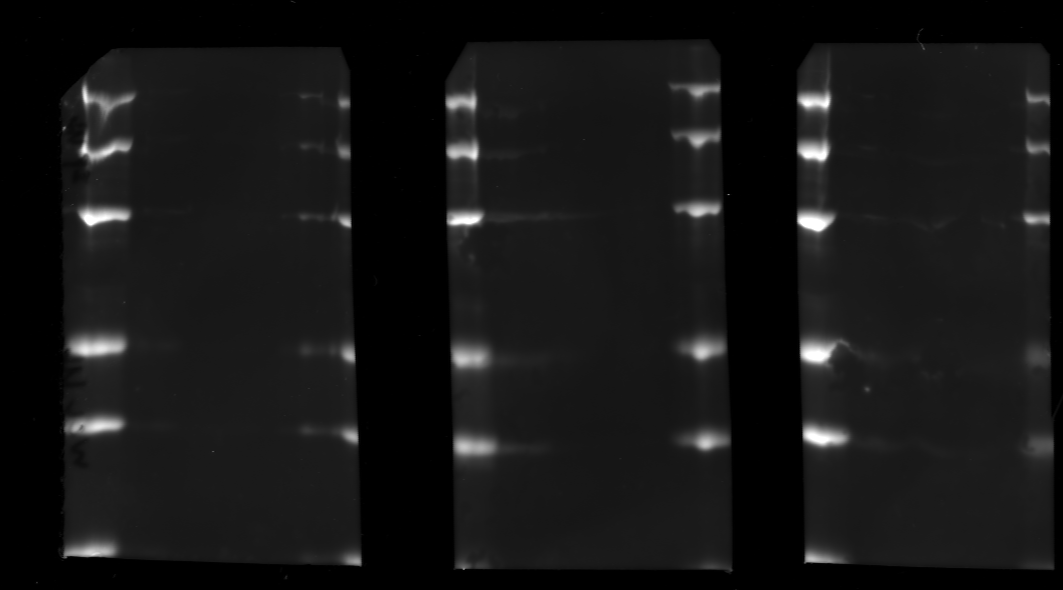

Supplement: Figure 4—figure supplement 1—source data 1. [file elife-68958-fig4-figsupp1-data1.zip › Figure 4-figure supplement 1ΓÇôsource data 1/Figure 4-figure supplement 1 full raw unedited blots files/original_files for G/2021-06-24-104007/700.TIF]

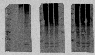

Supplement: Figure 4—figure supplement 1—source data 1. [file elife-68958-fig4-figsupp1-data1.zip › Figure 4-figure supplement 1ΓÇôsource data 1/Figure 4-figure supplement 1 full raw unedited blots files/original_files for G/2021-06-24-104007/2021-06-24-104007_4_TH.jpg]

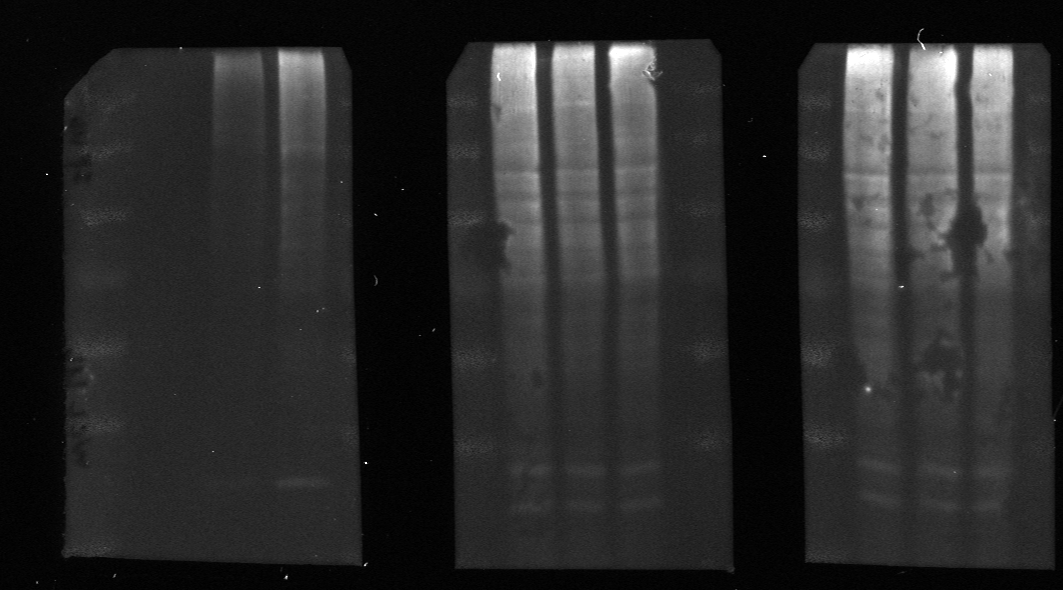

Supplement: Figure 4—figure supplement 1—source data 1. [file elife-68958-fig4-figsupp1-data1.zip › Figure 4-figure supplement 1ΓÇôsource data 1/Figure 4-figure supplement 1 full raw unedited blots files/original_files for G/2021-06-24-104007/800.TIF]

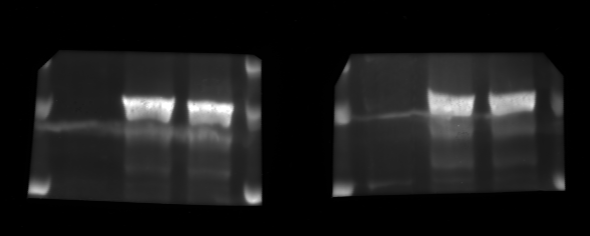

Supplement: Figure 4—figure supplement 1—source data 1. [file elife-68958-fig4-figsupp1-data1.zip › Figure 4-figure supplement 1ΓÇôsource data 1/Figure 4-figure supplement 1 full raw unedited blots files/original_files for G/2021-06-24-223452/700.TIF]

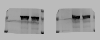

Supplement: Figure 4—figure supplement 1—source data 1. [file elife-68958-fig4-figsupp1-data1.zip › Figure 4-figure supplement 1ΓÇôsource data 1/Figure 4-figure supplement 1 full raw unedited blots files/original_files for G/2021-06-24-223452/2021-06-24-223452_GFP-RIG-I_TH.jpg]

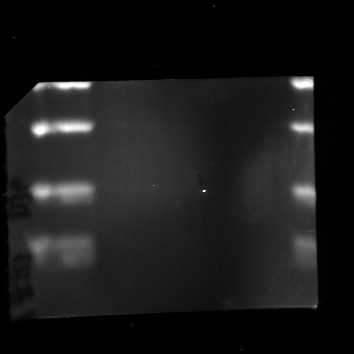

Supplement: Figure 4—figure supplement 1—source data 1. [file elife-68958-fig4-figsupp1-data1.zip › Figure 4-figure supplement 1ΓÇôsource data 1/Figure 4-figure supplement 1 full raw unedited blots files/original_files for G/2021-06-24-110159/700.TIF]

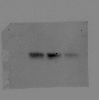

Supplement: Figure 4—figure supplement 1—source data 1. [file elife-68958-fig4-figsupp1-data1.zip › Figure 4-figure supplement 1ΓÇôsource data 1/Figure 4-figure supplement 1 full raw unedited blots files/original_files for G/2021-06-24-110159/2021-06-24-110159_RTN3_TH.jpg]

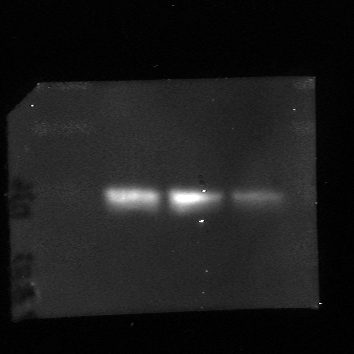

Supplement: Figure 4—figure supplement 1—source data 1. [file elife-68958-fig4-figsupp1-data1.zip › Figure 4-figure supplement 1ΓÇôsource data 1/Figure 4-figure supplement 1 full raw unedited blots files/original_files for G/2021-06-24-110159/800.TIF]

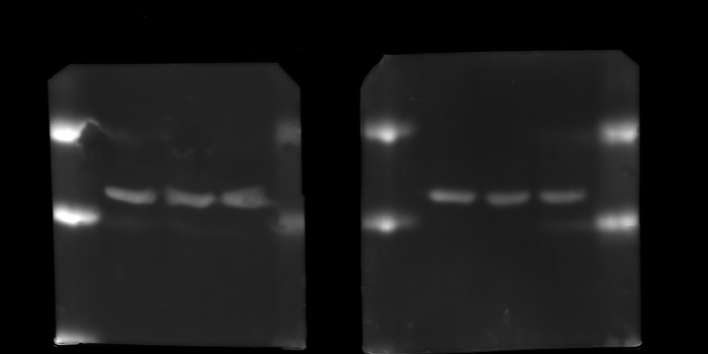

Supplement: Figure 4—figure supplement 1—source data 1. [file elife-68958-fig4-figsupp1-data1.zip › Figure 4-figure supplement 1ΓÇôsource data 1/Figure 4-figure supplement 1 full raw unedited blots files/original_files for G/2021-06-24-222200/700.TIF]

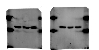

Supplement: Figure 4—figure supplement 1—source data 1. [file elife-68958-fig4-figsupp1-data1.zip › Figure 4-figure supplement 1ΓÇôsource data 1/Figure 4-figure supplement 1 full raw unedited blots files/original_files for G/2021-06-24-222200/2021-06-24-222200_actin_TH.jpg]

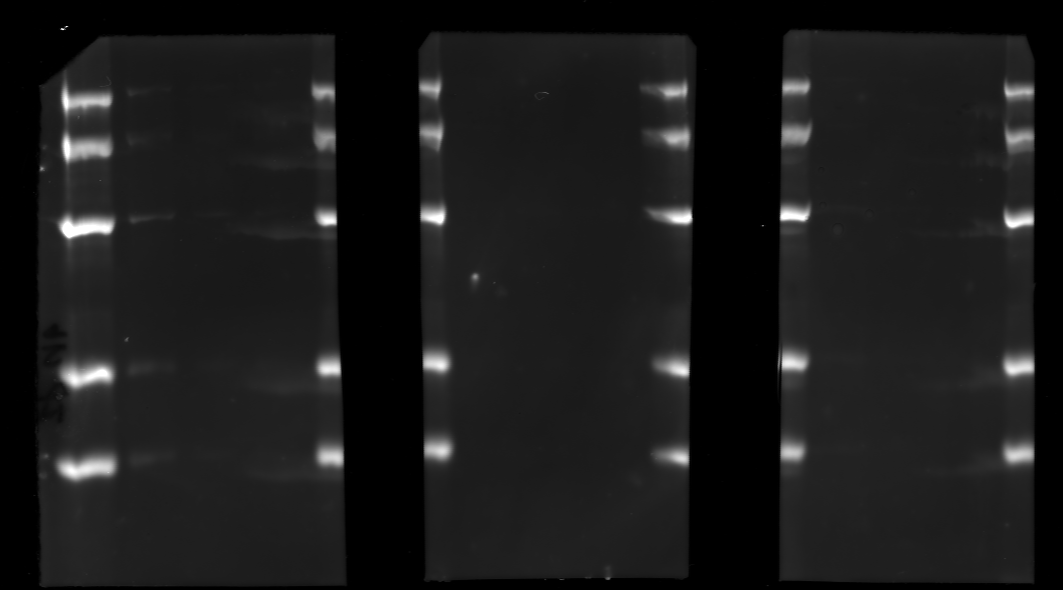

Supplement: Figure 4—figure supplement 1—source data 1. [file elife-68958-fig4-figsupp1-data1.zip › Figure 4-figure supplement 1ΓÇôsource data 1/Figure 4-figure supplement 1 full raw unedited blots files/original_files for G/2021-06-24-103151/700.TIF]

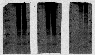

Supplement: Figure 4—figure supplement 1—source data 1. [file elife-68958-fig4-figsupp1-data1.zip › Figure 4-figure supplement 1ΓÇôsource data 1/Figure 4-figure supplement 1 full raw unedited blots files/original_files for G/2021-06-24-103151/2021-06-24-103151_3_TH.jpg]

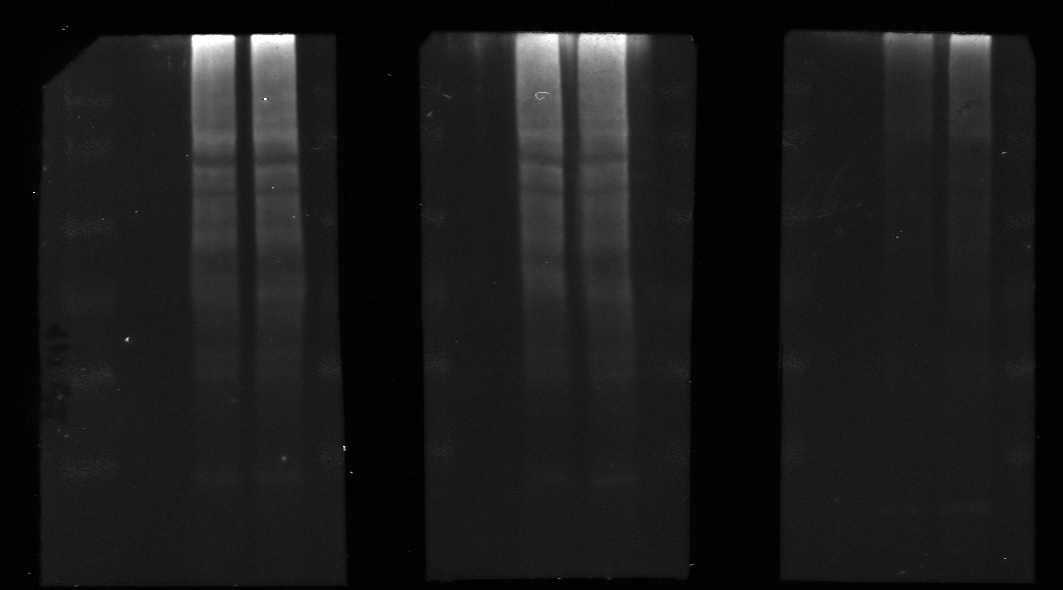

Supplement: Figure 4—figure supplement 1—source data 1. [file elife-68958-fig4-figsupp1-data1.zip › Figure 4-figure supplement 1ΓÇôsource data 1/Figure 4-figure supplement 1 full raw unedited blots files/original_files for G/2021-06-24-103151/800.TIF]

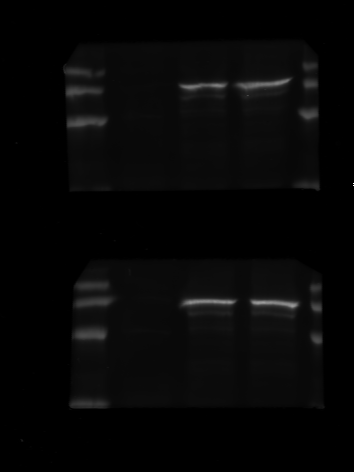

Supplement: Figure 4—figure supplement 1—source data 1. [file elife-68958-fig4-figsupp1-data1.zip › Figure 4-figure supplement 1ΓÇôsource data 1/Figure 4-figure supplement 1 full raw unedited blots files/original_files for G/2021-06-24-111622/700.TIF]

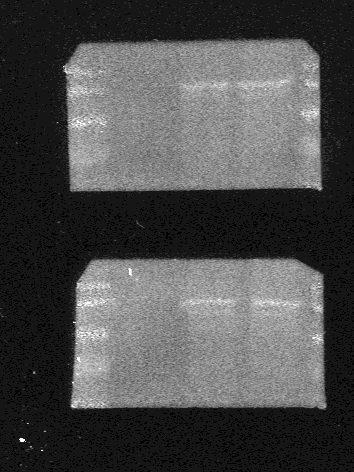

Supplement: Figure 4—figure supplement 1—source data 1. [file elife-68958-fig4-figsupp1-data1.zip › Figure 4-figure supplement 1ΓÇôsource data 1/Figure 4-figure supplement 1 full raw unedited blots files/original_files for G/2021-06-24-111622/800.TIF]

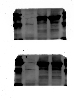

Supplement: Figure 4—figure supplement 1—source data 1. [file elife-68958-fig4-figsupp1-data1.zip › Figure 4-figure supplement 1ΓÇôsource data 1/Figure 4-figure supplement 1 full raw unedited blots files/original_files for G/2021-06-24-111622/2021-06-24-111622_GFP-RIG-I_TH.jpg]

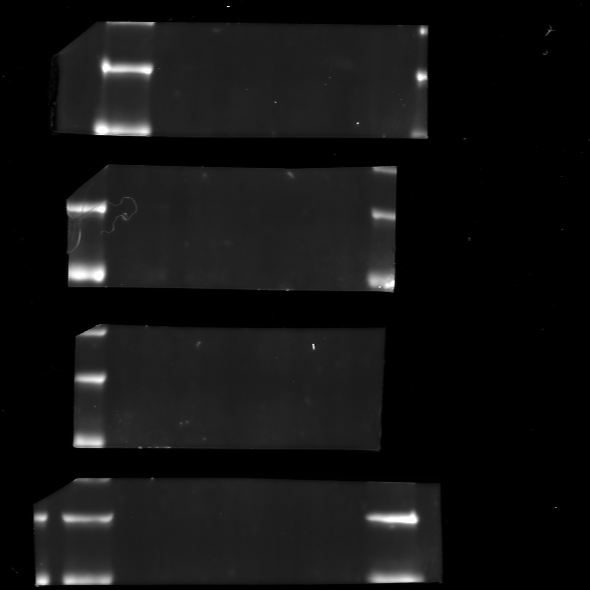

Supplement: Figure 4—figure supplement 1—source data 1. [file elife-68958-fig4-figsupp1-data1.zip › Figure 4-figure supplement 1ΓÇôsource data 1/Figure 4-figure supplement 1 full raw unedited blots files/original_files for I/2021-05-28-212549/700.TIF]

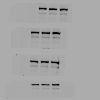

Supplement: Figure 4—figure supplement 1—source data 1. [file elife-68958-fig4-figsupp1-data1.zip › Figure 4-figure supplement 1ΓÇôsource data 1/Figure 4-figure supplement 1 full raw unedited blots files/original_files for I/2021-05-28-212549/2021-05-28-212549_GST T25_TH.jpg]

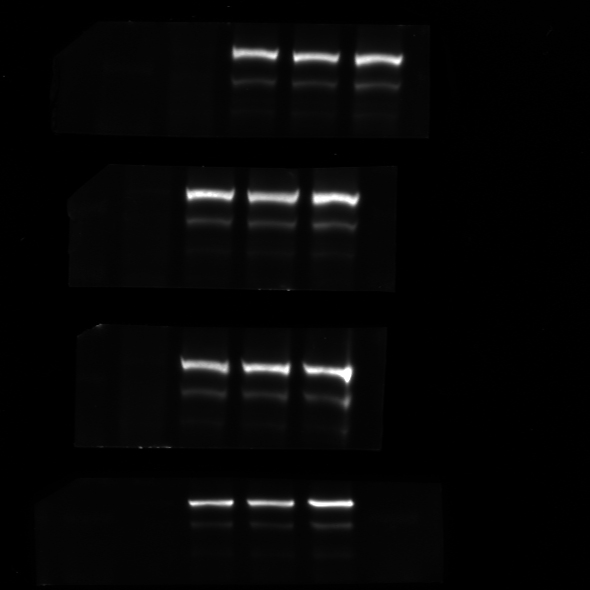

Supplement: Figure 4—figure supplement 1—source data 1. [file elife-68958-fig4-figsupp1-data1.zip › Figure 4-figure supplement 1ΓÇôsource data 1/Figure 4-figure supplement 1 full raw unedited blots files/original_files for I/2021-05-28-212549/800.TIF]

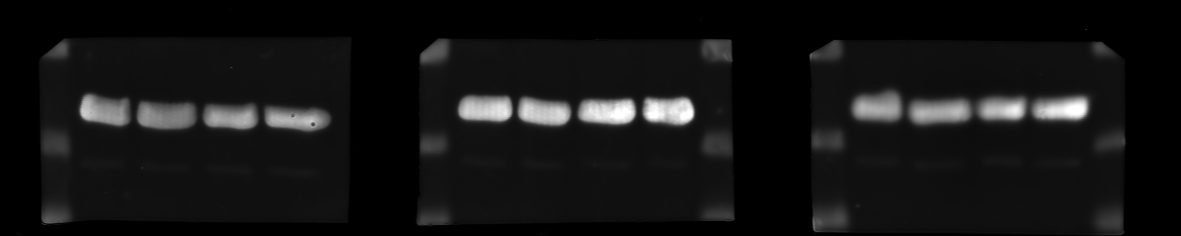

Supplement: Figure 4—figure supplement 1—source data 1. [file elife-68958-fig4-figsupp1-data1.zip › Figure 4-figure supplement 1ΓÇôsource data 1/Figure 4-figure supplement 1 full raw unedited blots files/original_files for I/2021-05-21-213336/700.TIF]
