# Supplementary figures and images for: RTN3 inhibits RIG-I-mediated antiviral responses by impairing TRIM25-mediated K63-linked polyubiquitination (part 3 of 3)
Source: eLife. 2021 Jul 27;10:e68958. doi: 10.7554/eLife.68958 (PMC8315805; doi:10.7554/eLife.68958)

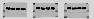

Supplement: Figure 4—figure supplement 1—source data 1. [file elife-68958-fig4-figsupp1-data1.zip › Figure 4-figure supplement 1ΓÇôsource data 1/Figure 4-figure supplement 1 full raw unedited blots files/original_files for I/2021-05-21-213336/2021-05-21-213336_actin_TH.jpg]

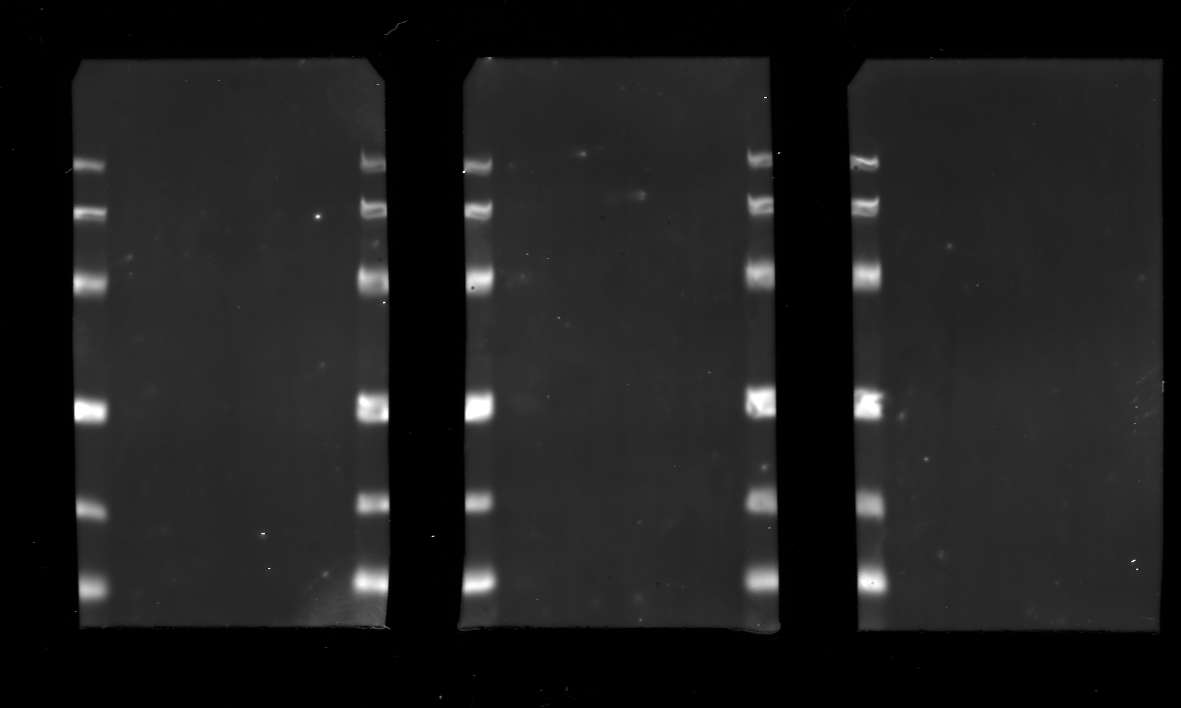

Supplement: Figure 4—figure supplement 1—source data 1. [file elife-68958-fig4-figsupp1-data1.zip › Figure 4-figure supplement 1ΓÇôsource data 1/Figure 4-figure supplement 1 full raw unedited blots files/original_files for I/2021-05-19-170459/700.TIF]

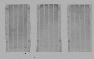

Supplement: Figure 4—figure supplement 1—source data 1. [file elife-68958-fig4-figsupp1-data1.zip › Figure 4-figure supplement 1ΓÇôsource data 1/Figure 4-figure supplement 1 full raw unedited blots files/original_files for I/2021-05-19-170459/2021-05-19-170459_4_TH.jpg]

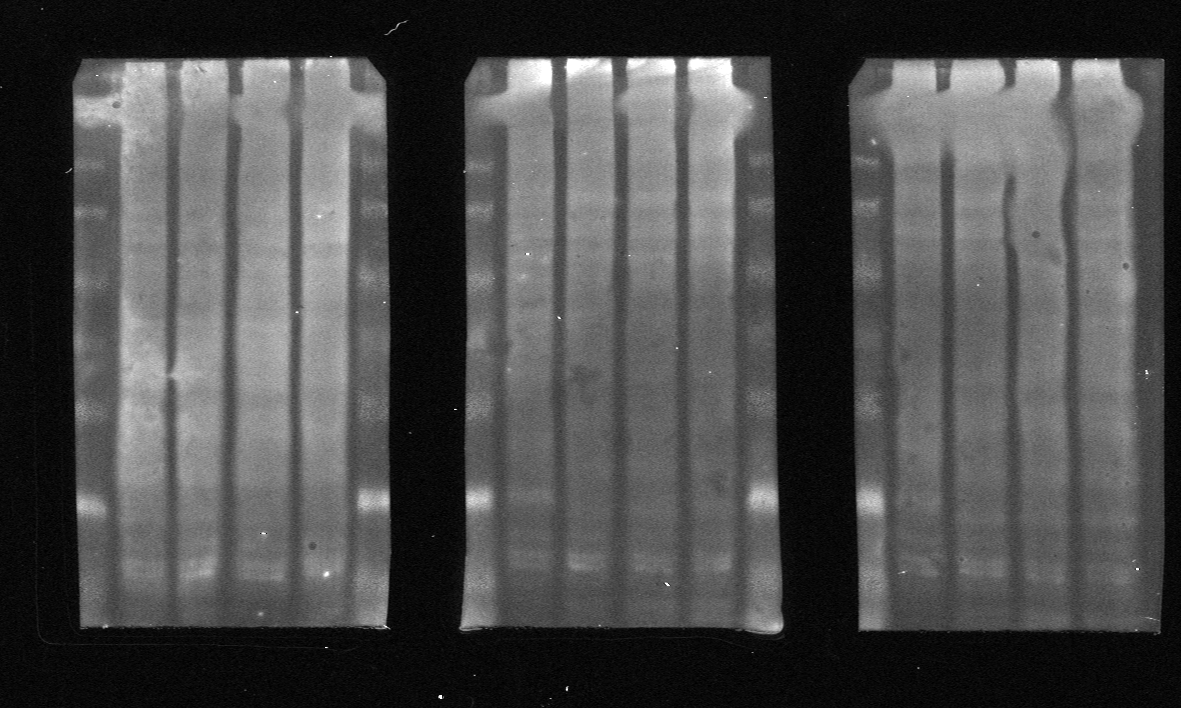

Supplement: Figure 4—figure supplement 1—source data 1. [file elife-68958-fig4-figsupp1-data1.zip › Figure 4-figure supplement 1ΓÇôsource data 1/Figure 4-figure supplement 1 full raw unedited blots files/original_files for I/2021-05-19-170459/800.TIF]

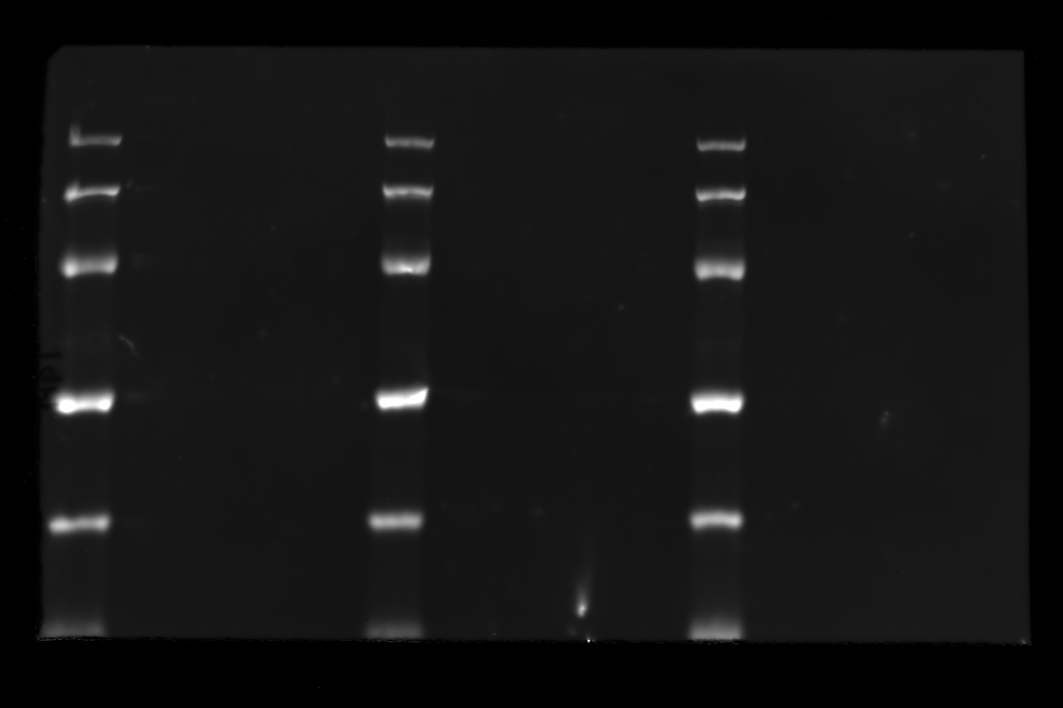

Supplement: Figure 4—figure supplement 1—source data 1. [file elife-68958-fig4-figsupp1-data1.zip › Figure 4-figure supplement 1ΓÇôsource data 1/Figure 4-figure supplement 1 full raw unedited blots files/original_files for I/2021-05-19-162659/700.TIF]

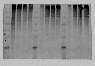

Supplement: Figure 4—figure supplement 1—source data 1. [file elife-68958-fig4-figsupp1-data1.zip › Figure 4-figure supplement 1ΓÇôsource data 1/Figure 4-figure supplement 1 full raw unedited blots files/original_files for I/2021-05-19-162659/2021-05-19-162659_1_TH.jpg]

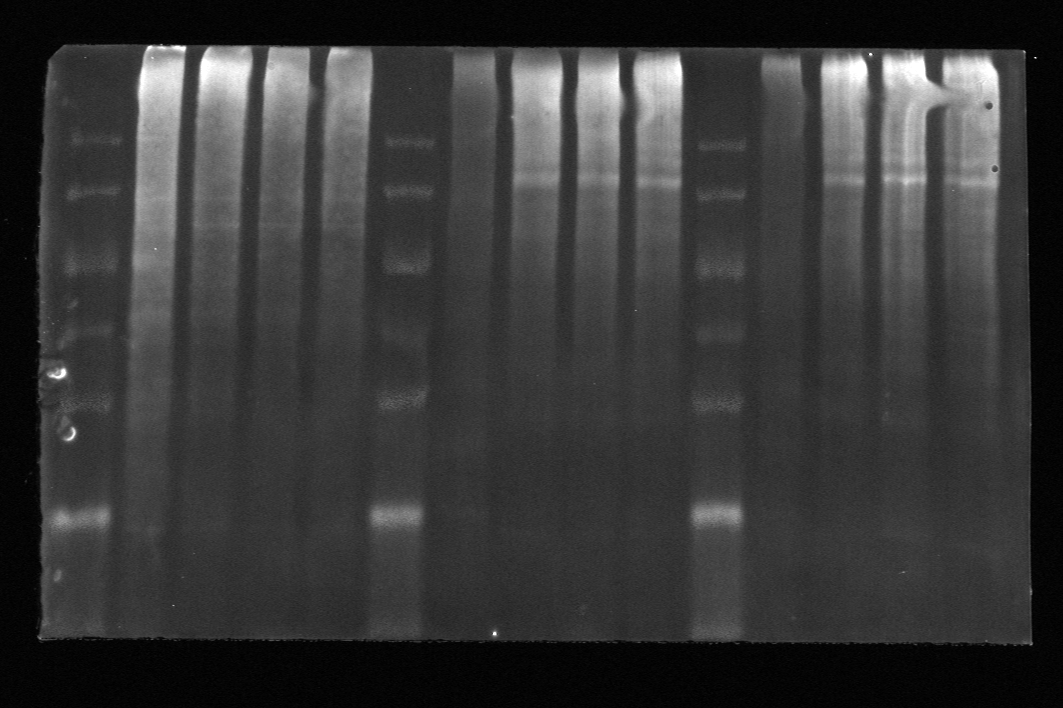

Supplement: Figure 4—figure supplement 1—source data 1. [file elife-68958-fig4-figsupp1-data1.zip › Figure 4-figure supplement 1ΓÇôsource data 1/Figure 4-figure supplement 1 full raw unedited blots files/original_files for I/2021-05-19-162659/800.TIF]

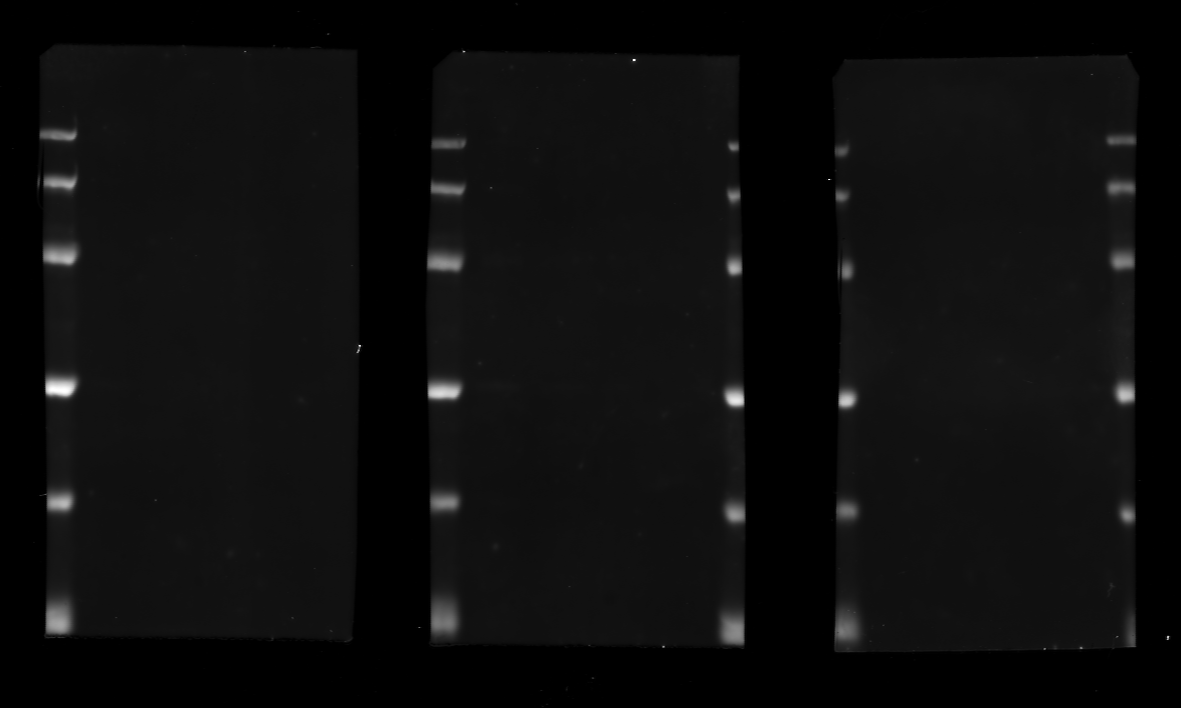

Supplement: Figure 4—figure supplement 1—source data 1. [file elife-68958-fig4-figsupp1-data1.zip › Figure 4-figure supplement 1ΓÇôsource data 1/Figure 4-figure supplement 1 full raw unedited blots files/original_files for H/2021-05-19-165539/700.TIF]

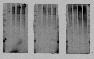

Supplement: Figure 4—figure supplement 1—source data 1. [file elife-68958-fig4-figsupp1-data1.zip › Figure 4-figure supplement 1ΓÇôsource data 1/Figure 4-figure supplement 1 full raw unedited blots files/original_files for H/2021-05-19-165539/2021-05-19-165539_3_TH.jpg]

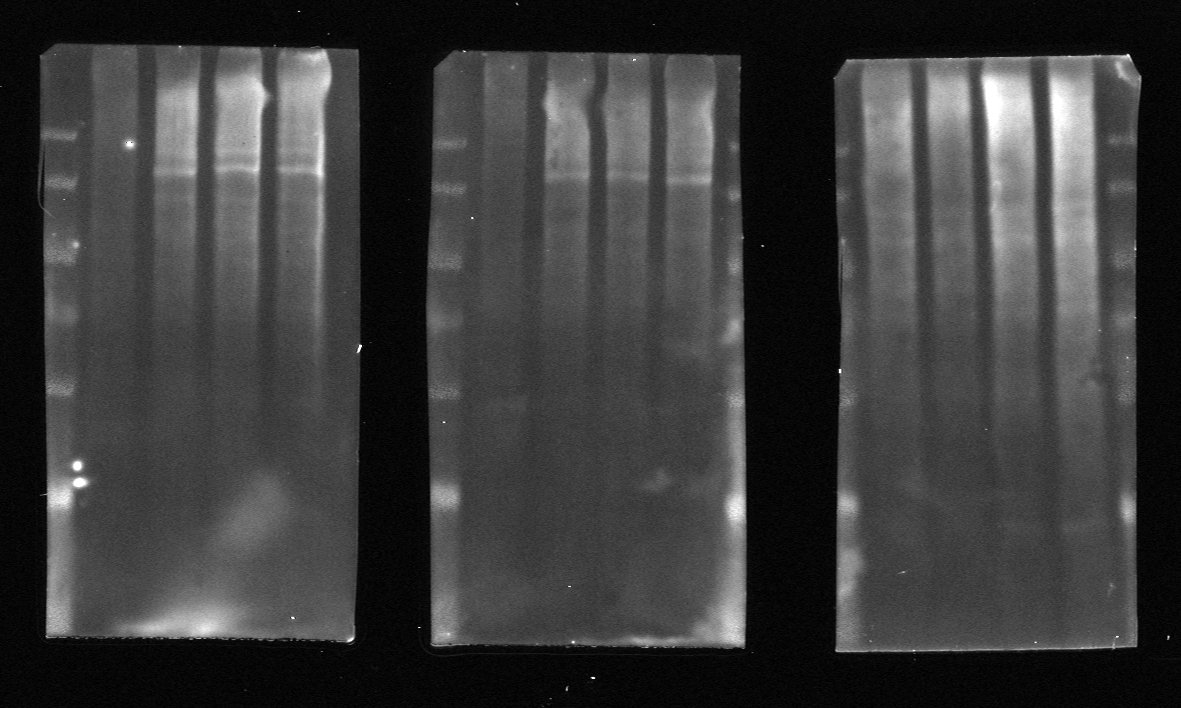

Supplement: Figure 4—figure supplement 1—source data 1. [file elife-68958-fig4-figsupp1-data1.zip › Figure 4-figure supplement 1ΓÇôsource data 1/Figure 4-figure supplement 1 full raw unedited blots files/original_files for H/2021-05-19-165539/800.TIF]

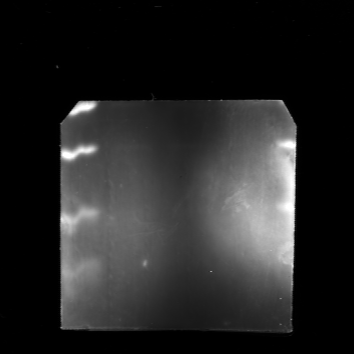

Supplement: Figure 4—figure supplement 1—source data 1. [file elife-68958-fig4-figsupp1-data1.zip › Figure 4-figure supplement 1ΓÇôsource data 1/Figure 4-figure supplement 1 full raw unedited blots files/original_files for F/2021-06-24-110608/700.TIF]

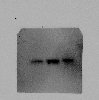

Supplement: Figure 4—figure supplement 1—source data 1. [file elife-68958-fig4-figsupp1-data1.zip › Figure 4-figure supplement 1ΓÇôsource data 1/Figure 4-figure supplement 1 full raw unedited blots files/original_files for F/2021-06-24-110608/2021-06-24-110608_RTN3 2 INVERT_TH.jpg]

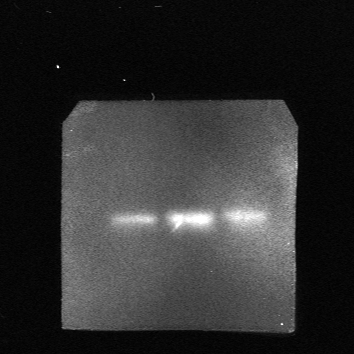

Supplement: Figure 4—figure supplement 1—source data 1. [file elife-68958-fig4-figsupp1-data1.zip › Figure 4-figure supplement 1ΓÇôsource data 1/Figure 4-figure supplement 1 full raw unedited blots files/original_files for F/2021-06-24-110608/800.TIF]

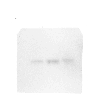

Supplement: Figure 4—figure supplement 1—source data 1. [file elife-68958-fig4-figsupp1-data1.zip › Figure 4-figure supplement 1ΓÇôsource data 1/Figure 4-figure supplement 1 full raw unedited blots files/original_files for F/2021-06-24-110608/2021-06-24-110608_6_TH.jpg]

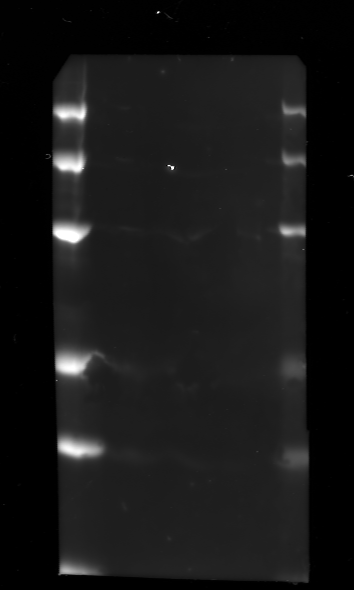

Supplement: Figure 4—figure supplement 1—source data 1. [file elife-68958-fig4-figsupp1-data1.zip › Figure 4-figure supplement 1ΓÇôsource data 1/Figure 4-figure supplement 1 full raw unedited blots files/original_files for F/2021-06-24-115231/700.TIF]

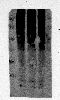

Supplement: Figure 4—figure supplement 1—source data 1. [file elife-68958-fig4-figsupp1-data1.zip › Figure 4-figure supplement 1ΓÇôsource data 1/Figure 4-figure supplement 1 full raw unedited blots files/original_files for F/2021-06-24-115231/2021-06-24-115231_input a-HA-ub-K63_TH.jpg]

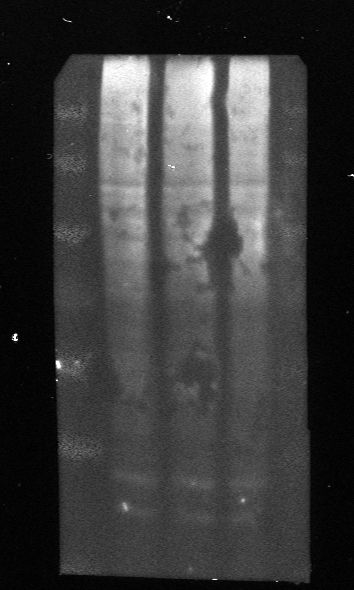

Supplement: Figure 4—figure supplement 1—source data 1. [file elife-68958-fig4-figsupp1-data1.zip › Figure 4-figure supplement 1ΓÇôsource data 1/Figure 4-figure supplement 1 full raw unedited blots files/original_files for F/2021-06-24-115231/800.TIF]

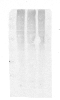

Supplement: Figure 4—figure supplement 1—source data 1. [file elife-68958-fig4-figsupp1-data1.zip › Figure 4-figure supplement 1ΓÇôsource data 1/Figure 4-figure supplement 1 full raw unedited blots files/original_files for F/2021-06-24-115231/2021-06-24-115231_input ub_TH.jpg]

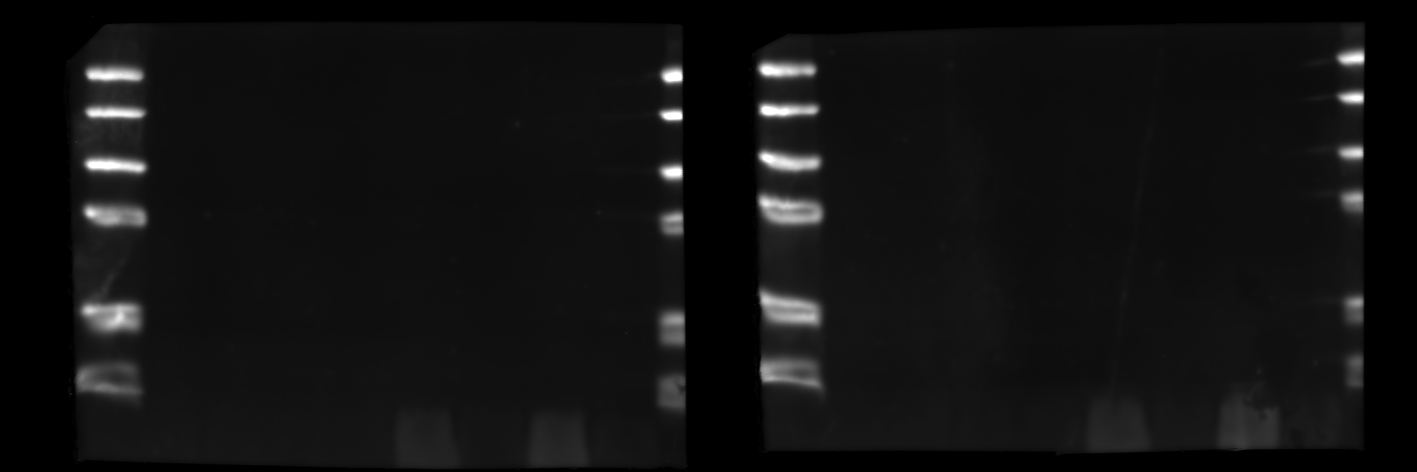

Supplement: Figure 5—source data 1. [file elife-68958-fig5-data1.zip › Figure 5ΓÇôsource data 1/Figure 5 full raw unedited blots files/original_files for C/2021-06-08-131512/700.TIF]

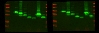

Supplement: Figure 5—source data 1. [file elife-68958-fig5-data1.zip › Figure 5ΓÇôsource data 1/Figure 5 full raw unedited blots files/original_files for C/2021-06-08-131512/2021-06-08-131512_RT3 TRUNCATION CONSTRUCTS_TH.jpg]

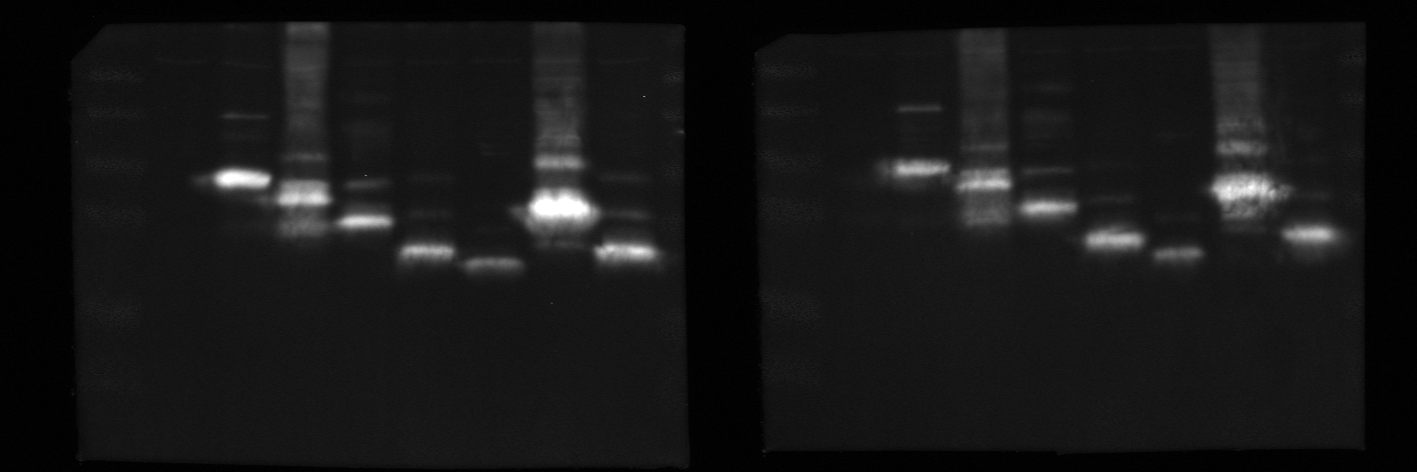

Supplement: Figure 5—source data 1. [file elife-68958-fig5-data1.zip › Figure 5ΓÇôsource data 1/Figure 5 full raw unedited blots files/original_files for C/2021-06-08-131512/800.TIF]

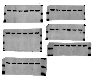

Supplement: Figure 5—source data 1. [file elife-68958-fig5-data1.zip › Figure 5ΓÇôsource data 1/Figure 5 full raw unedited blots files/original_files for B/2021-06-04-152757/2021-06-04-152757_actin_TH.jpg]

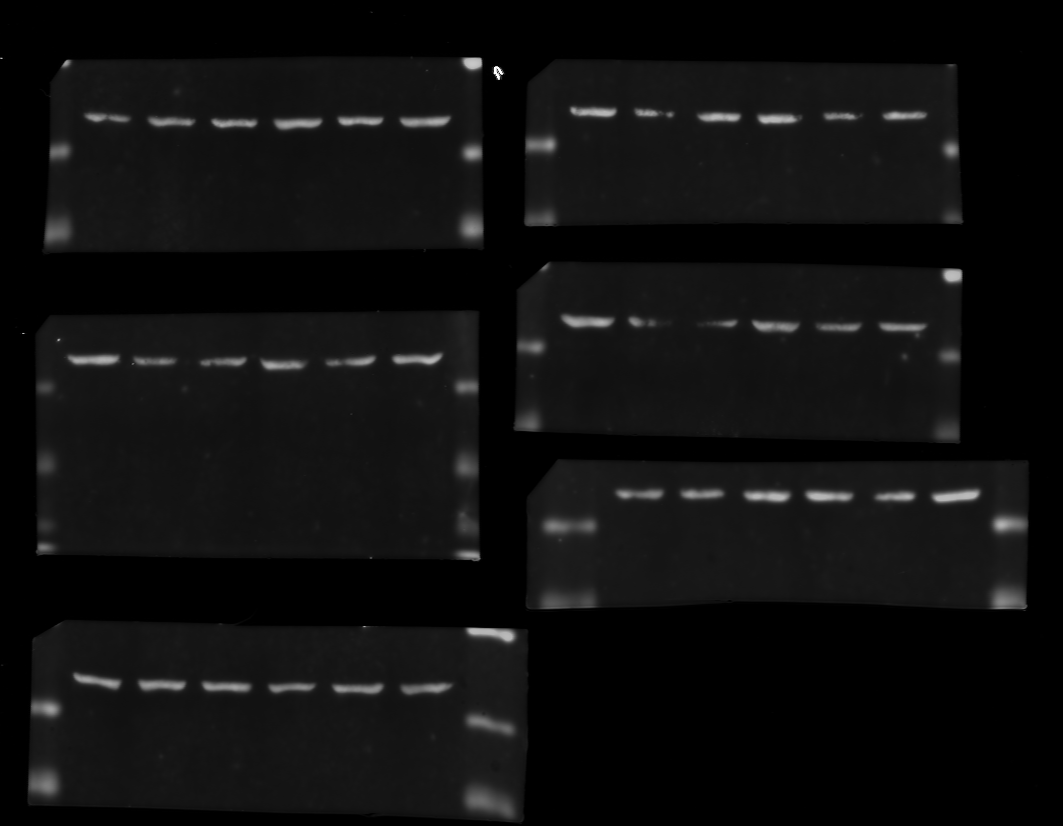

Supplement: Figure 5—source data 1. [file elife-68958-fig5-data1.zip › Figure 5ΓÇôsource data 1/Figure 5 full raw unedited blots files/original_files for B/2021-06-04-152757/700.TIF]

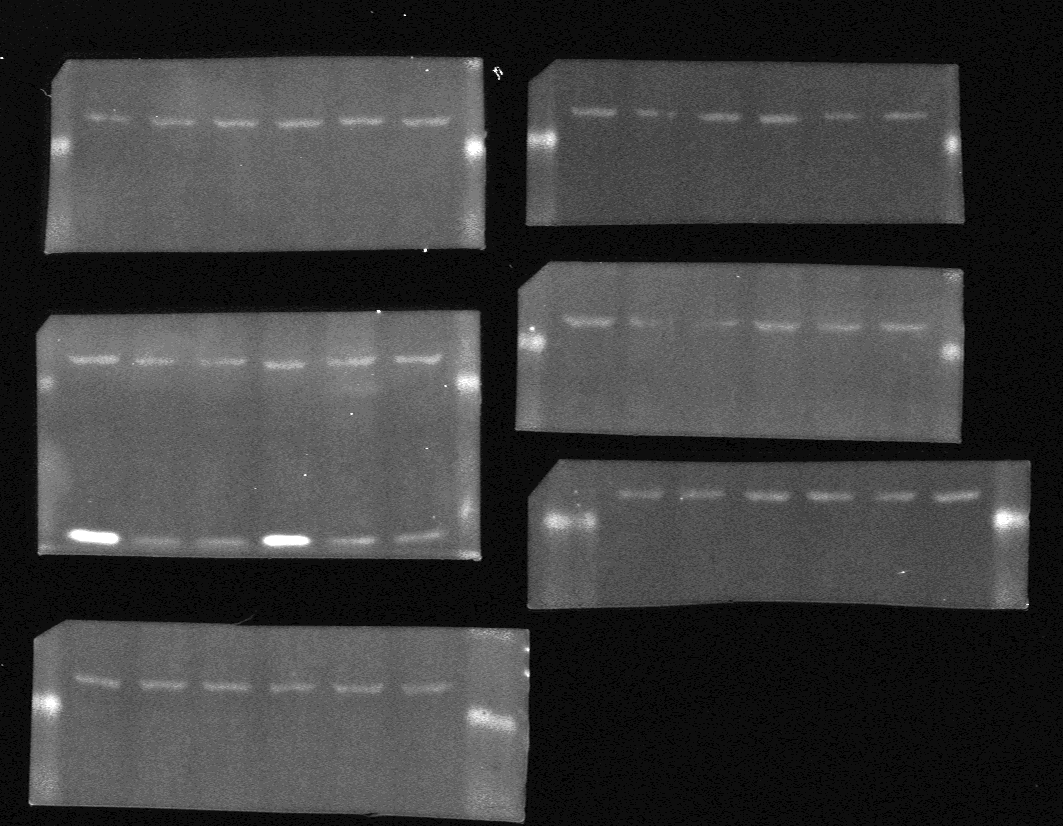

Supplement: Figure 5—source data 1. [file elife-68958-fig5-data1.zip › Figure 5ΓÇôsource data 1/Figure 5 full raw unedited blots files/original_files for B/2021-06-04-152757/800.TIF]

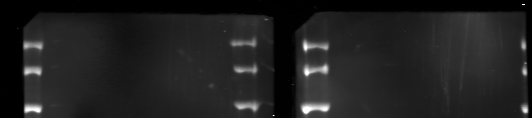

Supplement: Figure 5—source data 1. [file elife-68958-fig5-data1.zip › Figure 5ΓÇôsource data 1/Figure 5 full raw unedited blots files/original_files for B/2021-06-11-134018/700.TIF]

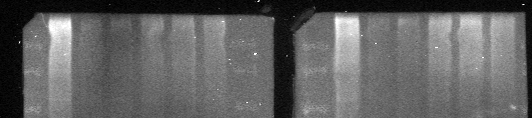

Supplement: Figure 5—source data 1. [file elife-68958-fig5-data1.zip › Figure 5ΓÇôsource data 1/Figure 5 full raw unedited blots files/original_files for B/2021-06-11-134018/800.TIF]

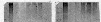

Supplement: Figure 5—source data 1. [file elife-68958-fig5-data1.zip › Figure 5ΓÇôsource data 1/Figure 5 full raw unedited blots files/original_files for B/2021-06-11-134018/2021-06-11-134018_3_TH.jpg]

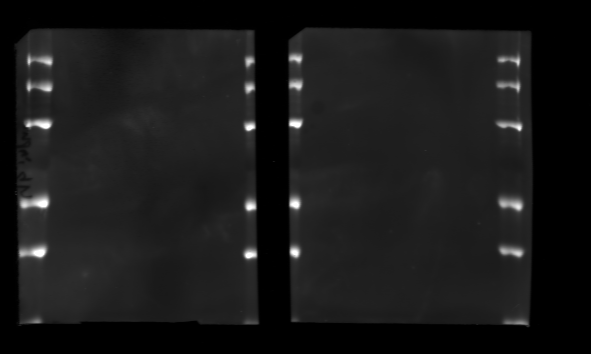

Supplement: Figure 5—source data 1. [file elife-68958-fig5-data1.zip › Figure 5ΓÇôsource data 1/Figure 5 full raw unedited blots files/original_files for B/2021-06-11-131627/700.TIF]

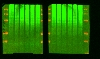

Supplement: Figure 5—source data 1. [file elife-68958-fig5-data1.zip › Figure 5ΓÇôsource data 1/Figure 5 full raw unedited blots files/original_files for B/2021-06-11-131627/2021-06-11-131627_UB-K63 input_TH.jpg]

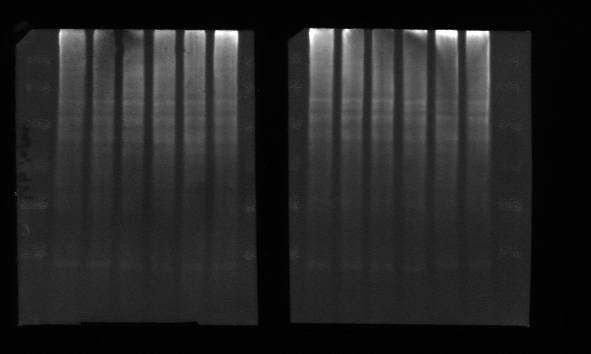

Supplement: Figure 5—source data 1. [file elife-68958-fig5-data1.zip › Figure 5ΓÇôsource data 1/Figure 5 full raw unedited blots files/original_files for B/2021-06-11-131627/800.TIF]

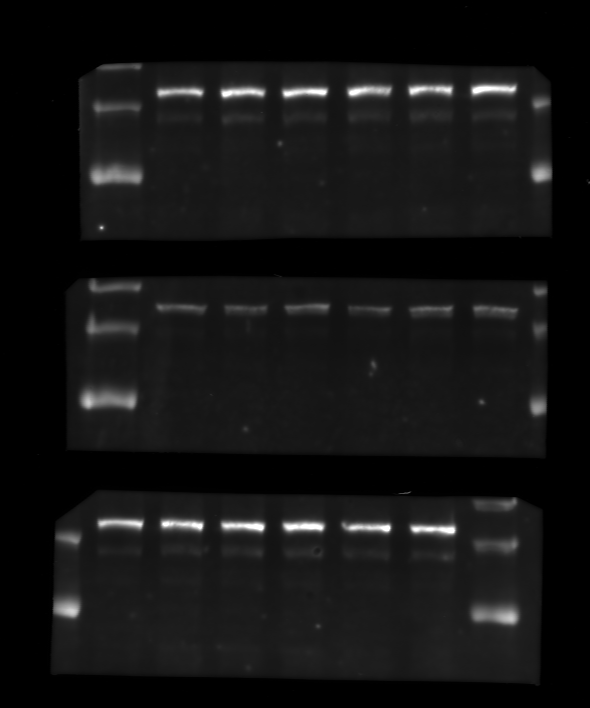

Supplement: Figure 5—source data 1. [file elife-68958-fig5-data1.zip › Figure 5ΓÇôsource data 1/Figure 5 full raw unedited blots files/original_files for B/2021-06-05-163933/700.TIF]

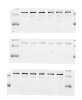

Supplement: Figure 5—source data 1. [file elife-68958-fig5-data1.zip › Figure 5ΓÇôsource data 1/Figure 5 full raw unedited blots files/original_files for B/2021-06-05-163933/2021-06-05-163933_GFP RIG-I IP INPUT_TH.jpg]

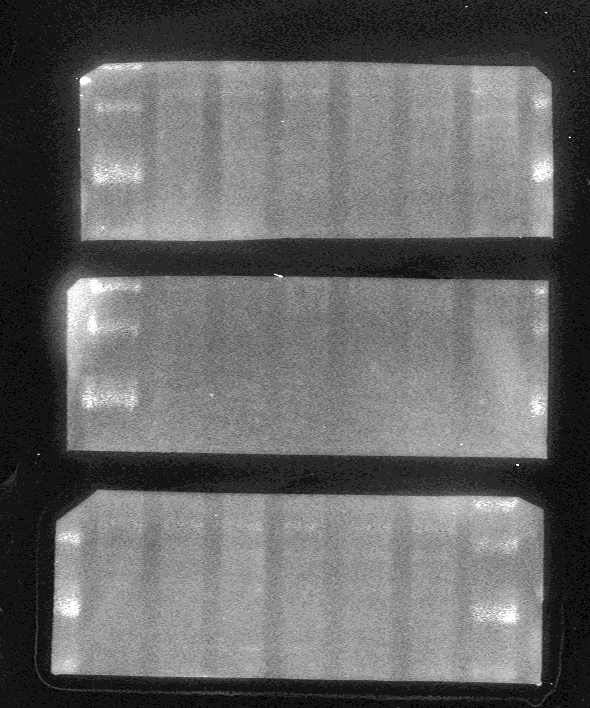

Supplement: Figure 5—source data 1. [file elife-68958-fig5-data1.zip › Figure 5ΓÇôsource data 1/Figure 5 full raw unedited blots files/original_files for B/2021-06-05-163933/800.TIF]

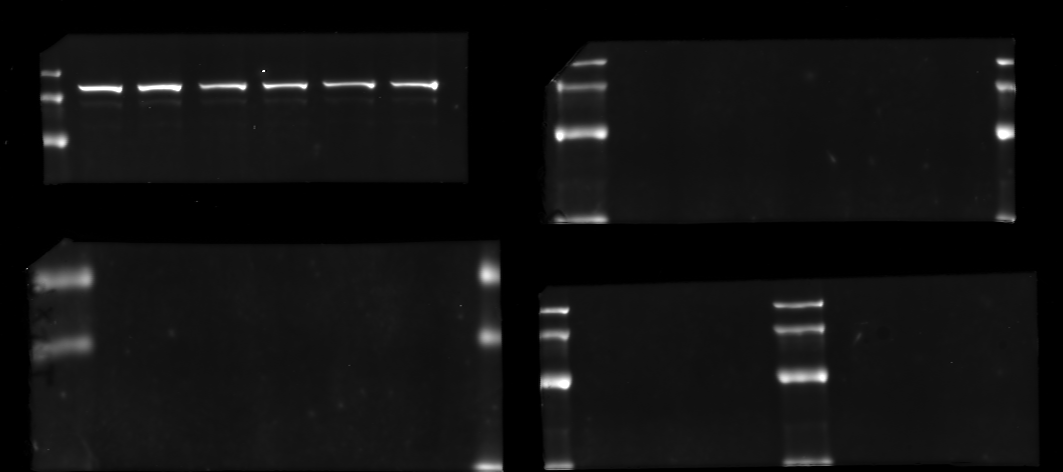

Supplement: Figure 5—source data 1. [file elife-68958-fig5-data1.zip › Figure 5ΓÇôsource data 1/Figure 5 full raw unedited blots files/original_files for B/2021-05-31-164108/700.TIF]

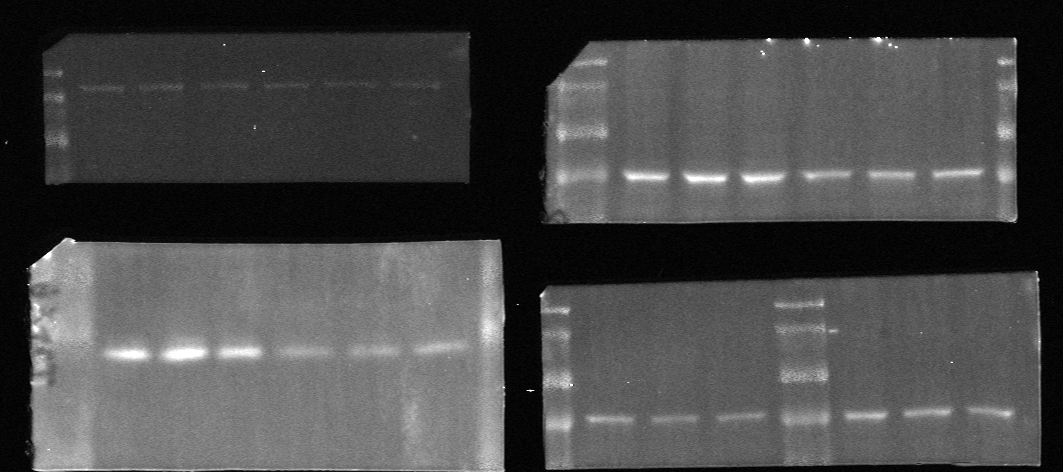

Supplement: Figure 5—source data 1. [file elife-68958-fig5-data1.zip › Figure 5ΓÇôsource data 1/Figure 5 full raw unedited blots files/original_files for B/2021-05-31-164108/800.TIF]

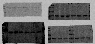

Supplement: Figure 5—source data 1. [file elife-68958-fig5-data1.zip › Figure 5ΓÇôsource data 1/Figure 5 full raw unedited blots files/original_files for B/2021-05-31-164108/2021-05-31-164108_GFP RIGI T25NCKD RT3NCKD_TH.jpg]

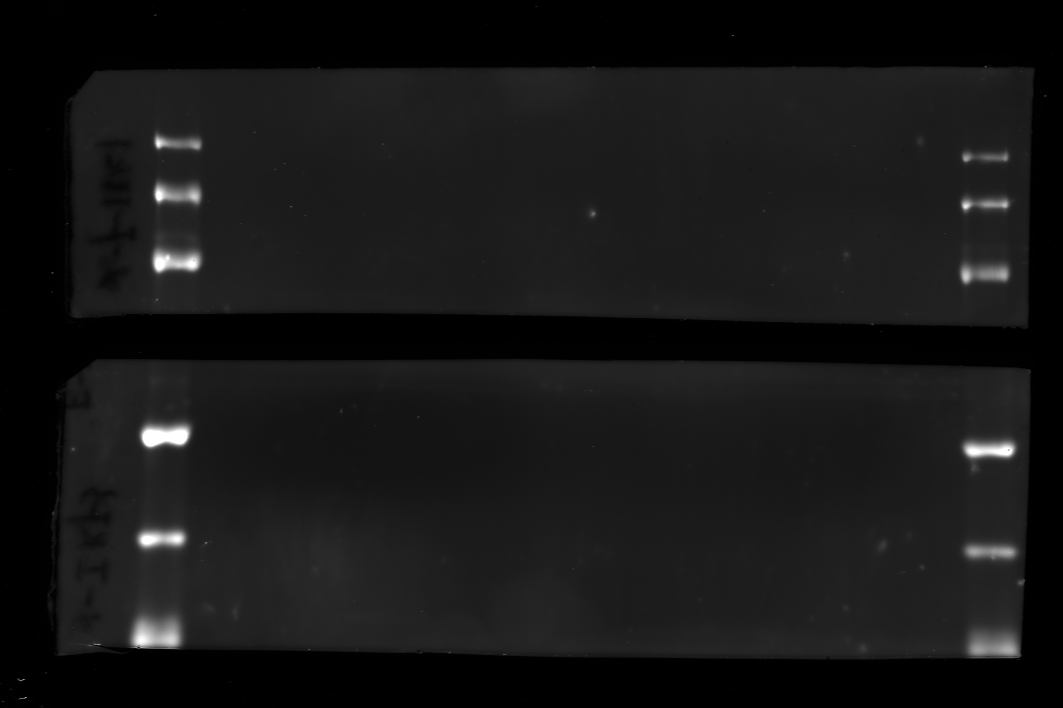

Supplement: Figure 5—source data 1. [file elife-68958-fig5-data1.zip › Figure 5ΓÇôsource data 1/Figure 5 full raw unedited blots files/original_files for A/2021-03-13-195729/700.TIF]

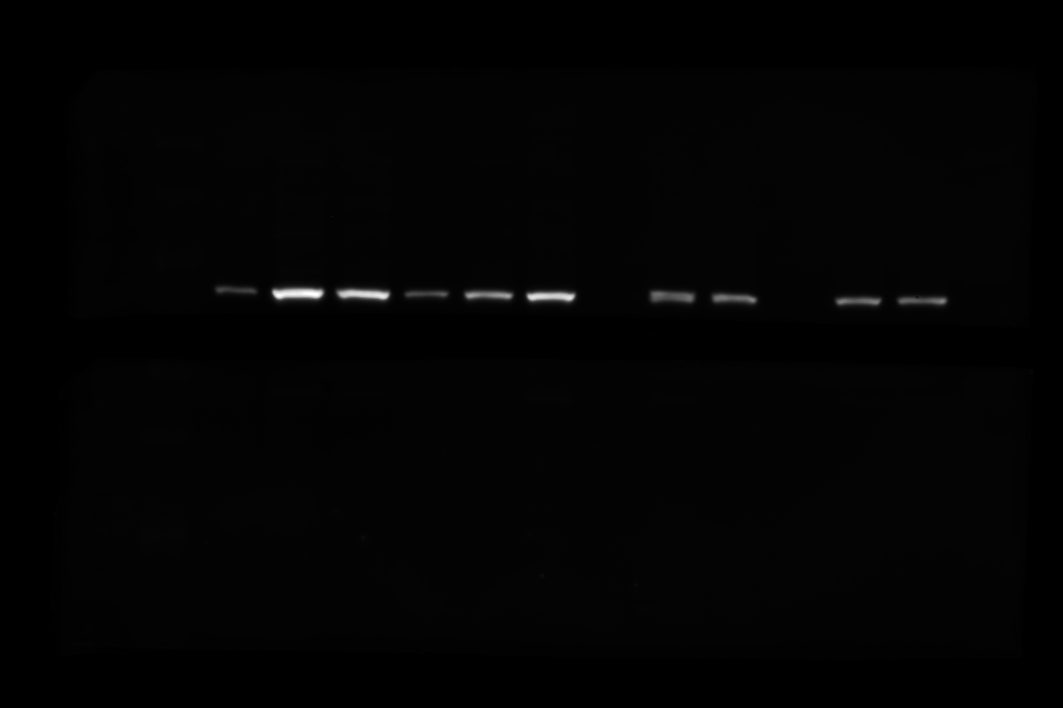

Supplement: Figure 5—source data 1. [file elife-68958-fig5-data1.zip › Figure 5ΓÇôsource data 1/Figure 5 full raw unedited blots files/original_files for A/2021-03-13-195729/800.TIF]

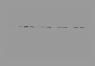

Supplement: Figure 5—source data 1. [file elife-68958-fig5-data1.zip › Figure 5ΓÇôsource data 1/Figure 5 full raw unedited blots files/original_files for A/2021-03-13-195729/2021-03-13-195729_a-pTBK1 pIRF3_TH.jpg]

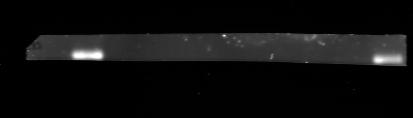

Supplement: Figure 5—source data 1. [file elife-68958-fig5-data1.zip › Figure 5ΓÇôsource data 1/Figure 5 full raw unedited blots files/original_files for A/2020-10-04-170321/700.TIF]

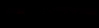

Supplement: Figure 5—source data 1. [file elife-68958-fig5-data1.zip › Figure 5ΓÇôsource data 1/Figure 5 full raw unedited blots files/original_files for A/2020-10-04-170321/2020-10-04-170321_8_TH.jpg]

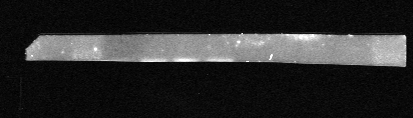

Supplement: Figure 5—source data 1. [file elife-68958-fig5-data1.zip › Figure 5ΓÇôsource data 1/Figure 5 full raw unedited blots files/original_files for A/2020-10-04-170321/800.TIF]

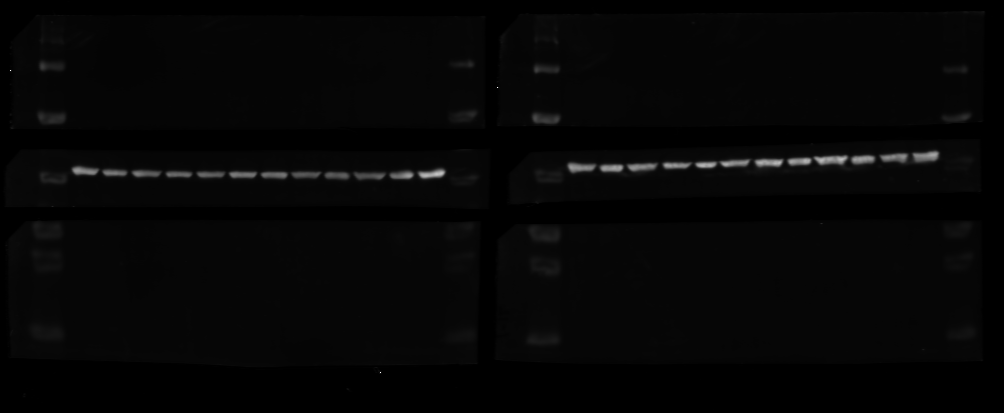

Supplement: Figure 5—source data 1. [file elife-68958-fig5-data1.zip › Figure 5ΓÇôsource data 1/Figure 5 full raw unedited blots files/original_files for A/2020-08-24-204931/700.TIF]

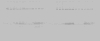

Supplement: Figure 5—source data 1. [file elife-68958-fig5-data1.zip › Figure 5ΓÇôsource data 1/Figure 5 full raw unedited blots files/original_files for A/2020-08-24-204931/2020-08-24-204931_a-T25 ACTIN HA-RT3_TH.jpg]

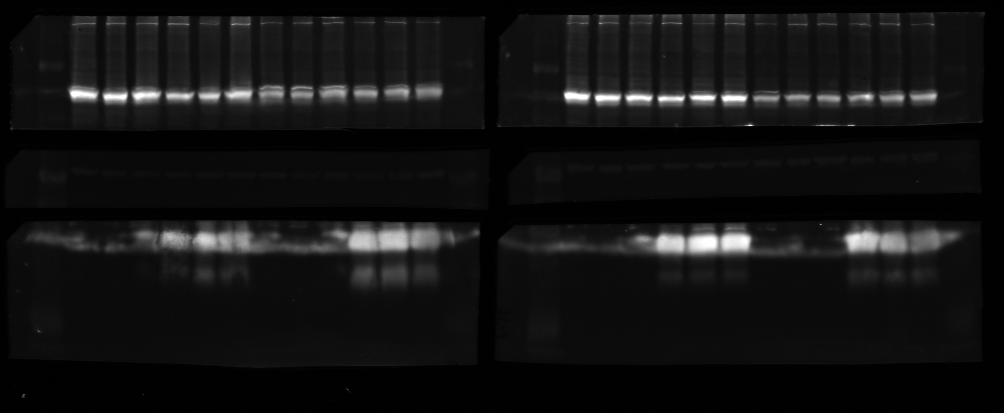

Supplement: Figure 5—source data 1. [file elife-68958-fig5-data1.zip › Figure 5ΓÇôsource data 1/Figure 5 full raw unedited blots files/original_files for A/2020-08-24-204931/800.TIF]

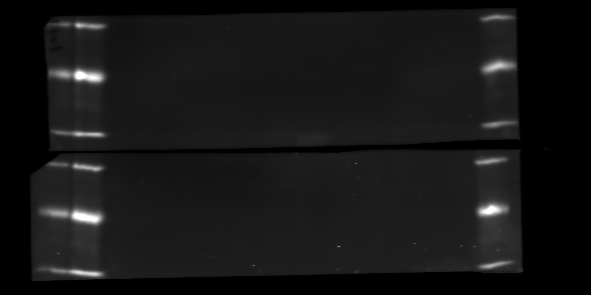

Supplement: Figure 5—source data 1. [file elife-68958-fig5-data1.zip › Figure 5ΓÇôsource data 1/Figure 5 full raw unedited blots files/original_files for A/2020-05-07-114737/700.TIF]

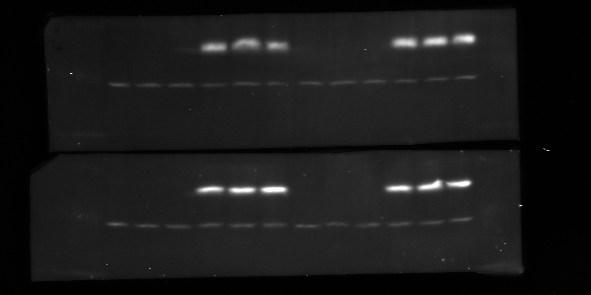

Supplement: Figure 5—source data 1. [file elife-68958-fig5-data1.zip › Figure 5ΓÇôsource data 1/Figure 5 full raw unedited blots files/original_files for A/2020-05-07-114737/800.TIF]

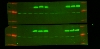

Supplement: Figure 5—source data 1. [file elife-68958-fig5-data1.zip › Figure 5ΓÇôsource data 1/Figure 5 full raw unedited blots files/original_files for A/2020-05-07-114737/2020-05-07-114737_a-Flag P8_TH.jpg]

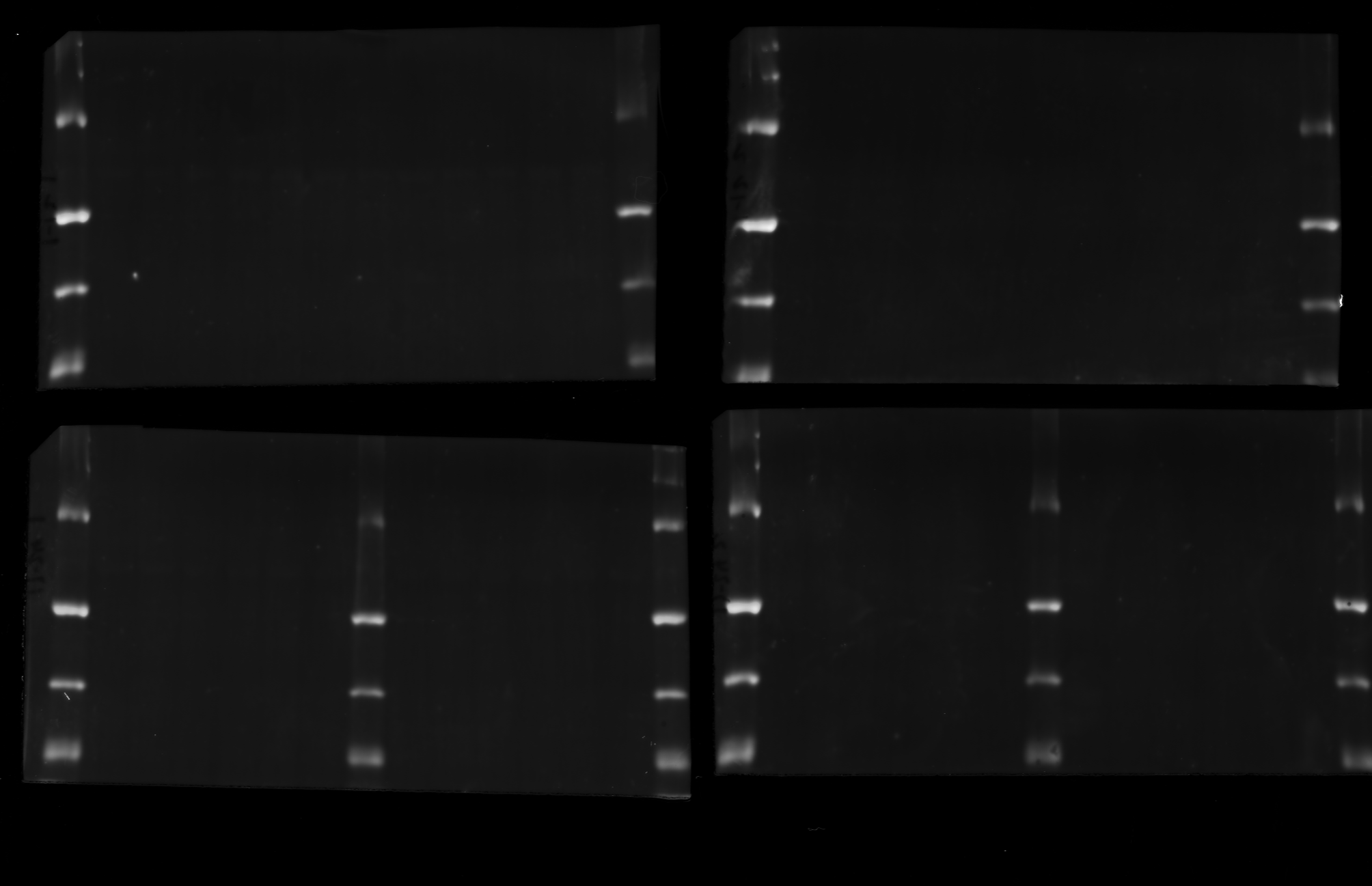

Supplement: Figure 5—source data 1. [file elife-68958-fig5-data1.zip › Figure 5ΓÇôsource data 1/Figure 5 full raw unedited blots files/original_files for A/2021-03-09-163511/700.TIF]

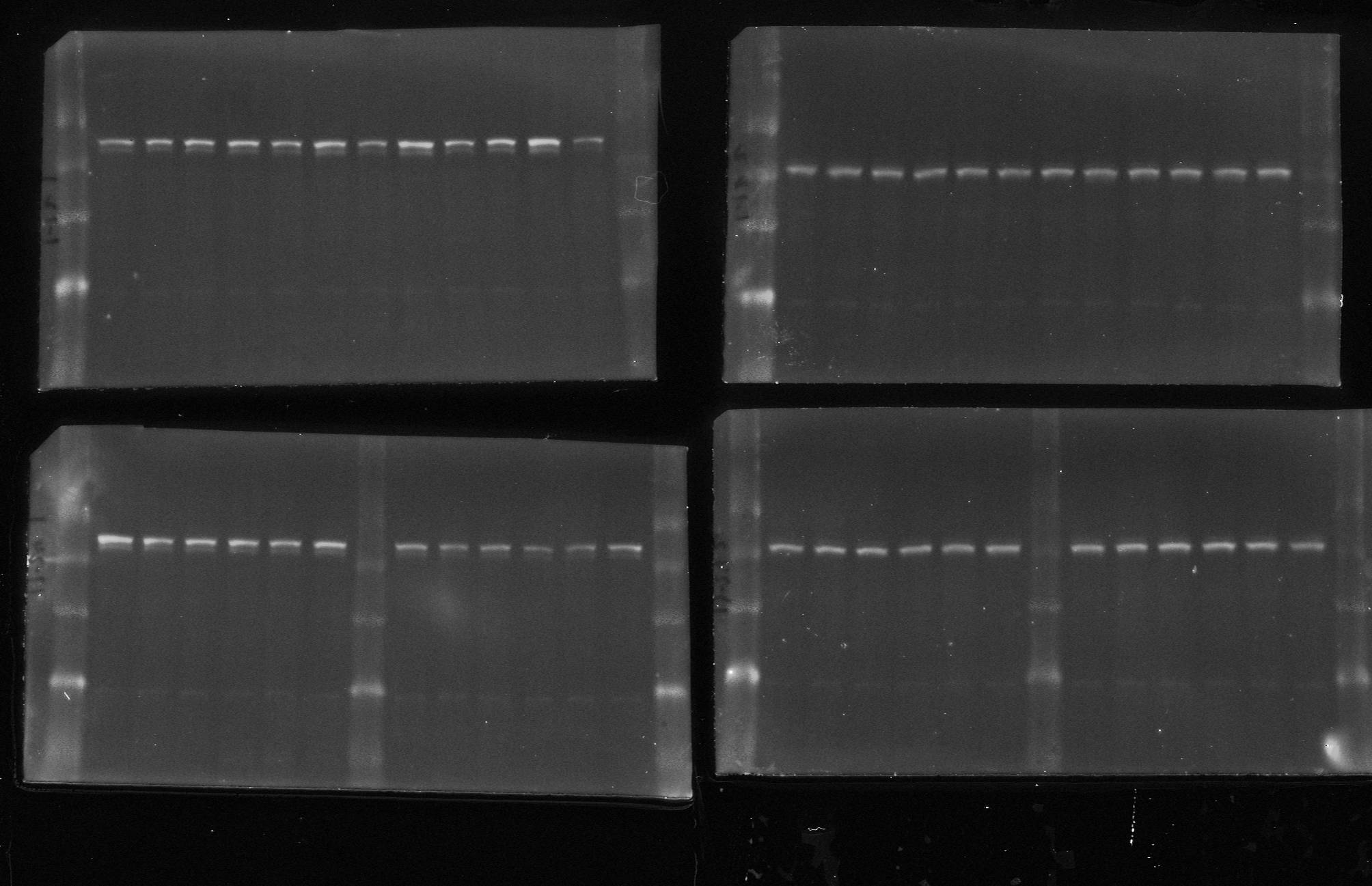

Supplement: Figure 5—source data 1. [file elife-68958-fig5-data1.zip › Figure 5ΓÇôsource data 1/Figure 5 full raw unedited blots files/original_files for A/2021-03-09-163511/800.TIF]

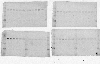

Supplement: Figure 5—source data 1. [file elife-68958-fig5-data1.zip › Figure 5ΓÇôsource data 1/Figure 5 full raw unedited blots files/original_files for A/2021-03-09-163511/2021-03-09-163511_a-pP65 pIRF3_TH.jpg]

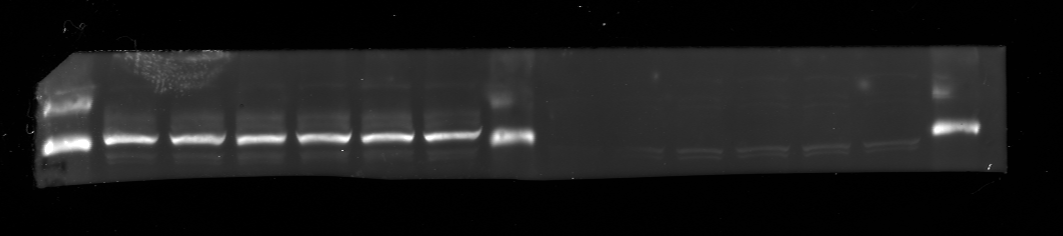

Supplement: Figure 5—figure supplement 1—source data 1. [file elife-68958-fig5-figsupp1-data1.zip › Figure 5-figure supplement 1ΓÇôsource data 1/Figure 5-figure supplement 1 full raw unedited blots files/original_files for C/2021-02-28-200737/700.TIF]

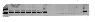

Supplement: Figure 5—figure supplement 1—source data 1. [file elife-68958-fig5-figsupp1-data1.zip › Figure 5-figure supplement 1ΓÇôsource data 1/Figure 5-figure supplement 1 full raw unedited blots files/original_files for C/2021-02-28-200737/2021-02-28-200737_a-GST-MDA5_TH.jpg]

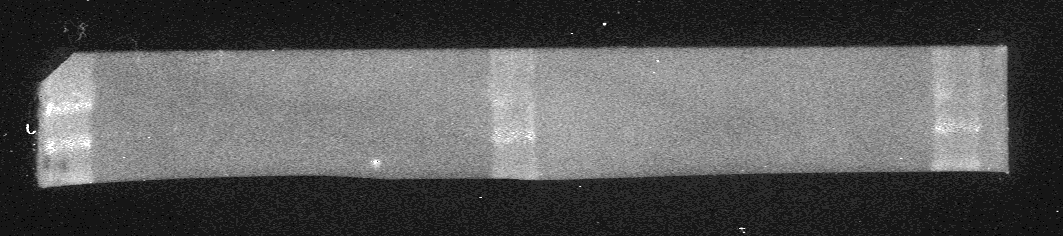

Supplement: Figure 5—figure supplement 1—source data 1. [file elife-68958-fig5-figsupp1-data1.zip › Figure 5-figure supplement 1ΓÇôsource data 1/Figure 5-figure supplement 1 full raw unedited blots files/original_files for C/2021-02-28-200737/800.TIF]

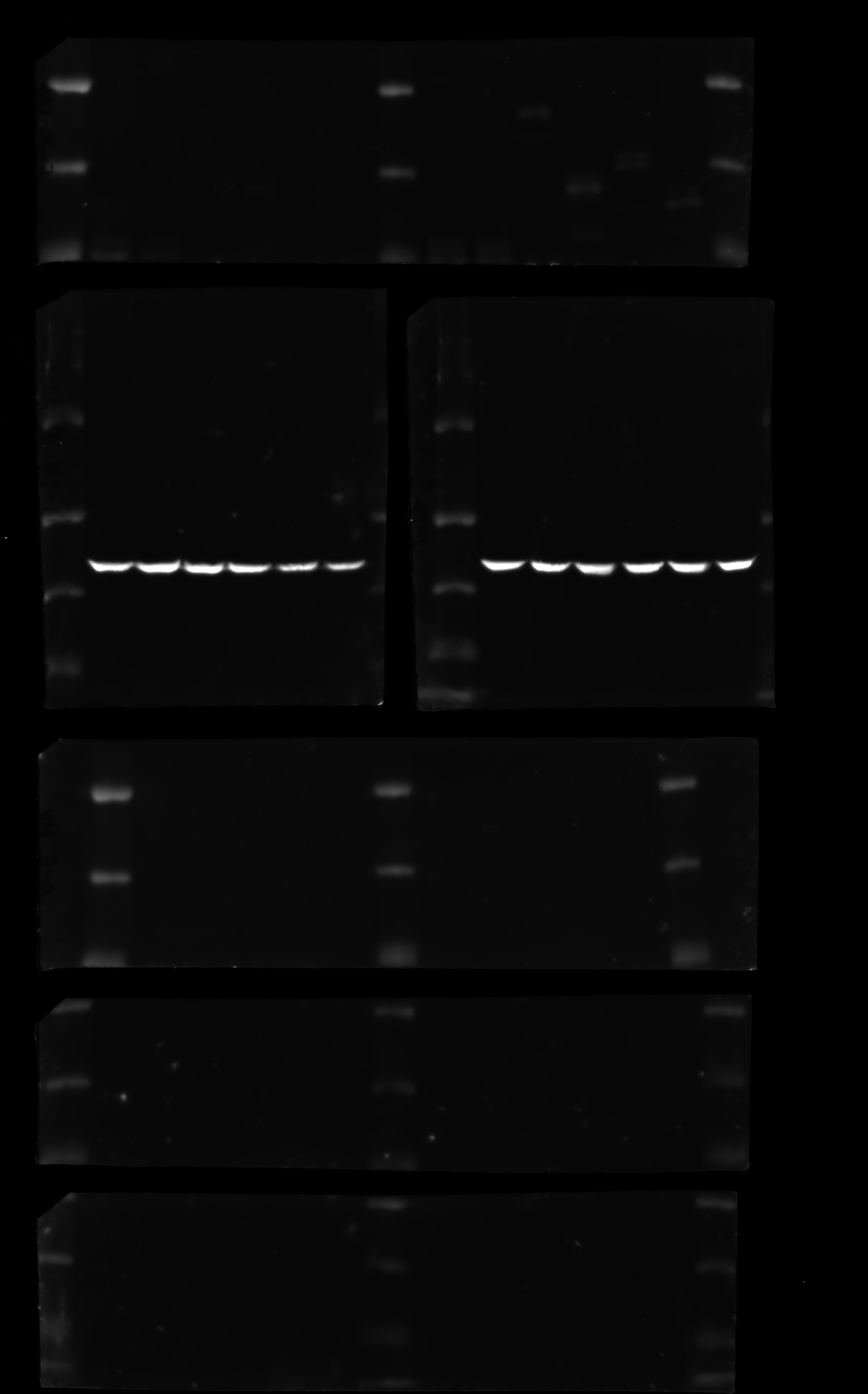

Supplement: Figure 5—figure supplement 1—source data 1. [file elife-68958-fig5-figsupp1-data1.zip › Figure 5-figure supplement 1ΓÇôsource data 1/Figure 5-figure supplement 1 full raw unedited blots files/original_files for C/2021-02-08-140745/700.TIF]

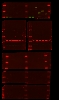

Supplement: Figure 5—figure supplement 1—source data 1. [file elife-68958-fig5-figsupp1-data1.zip › Figure 5-figure supplement 1ΓÇôsource data 1/Figure 5-figure supplement 1 full raw unedited blots files/original_files for C/2021-02-08-140745/2021-02-08-140745_a-ACTIN_TH.jpg]

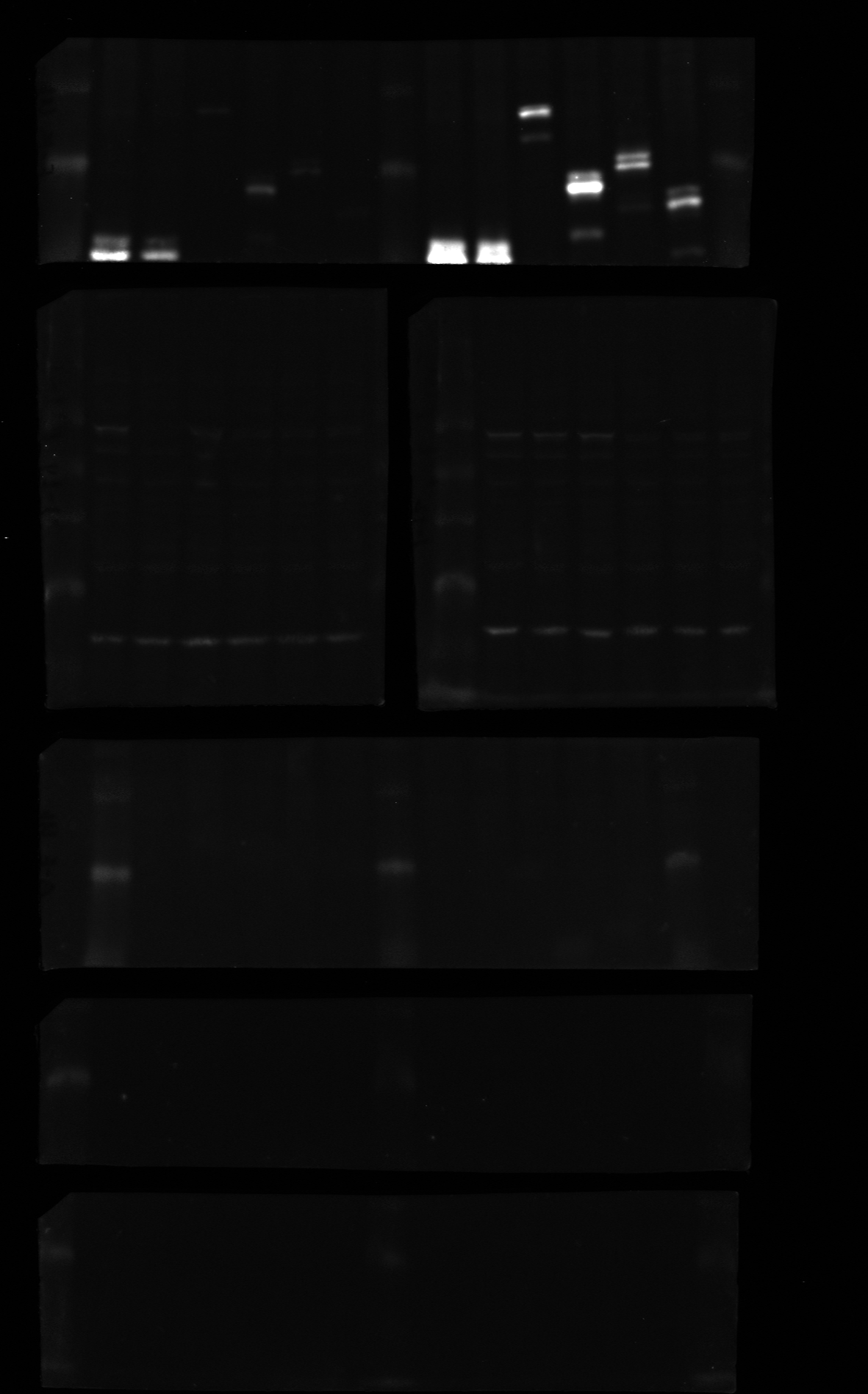

Supplement: Figure 5—figure supplement 1—source data 1. [file elife-68958-fig5-figsupp1-data1.zip › Figure 5-figure supplement 1ΓÇôsource data 1/Figure 5-figure supplement 1 full raw unedited blots files/original_files for C/2021-02-08-140745/800.TIF]

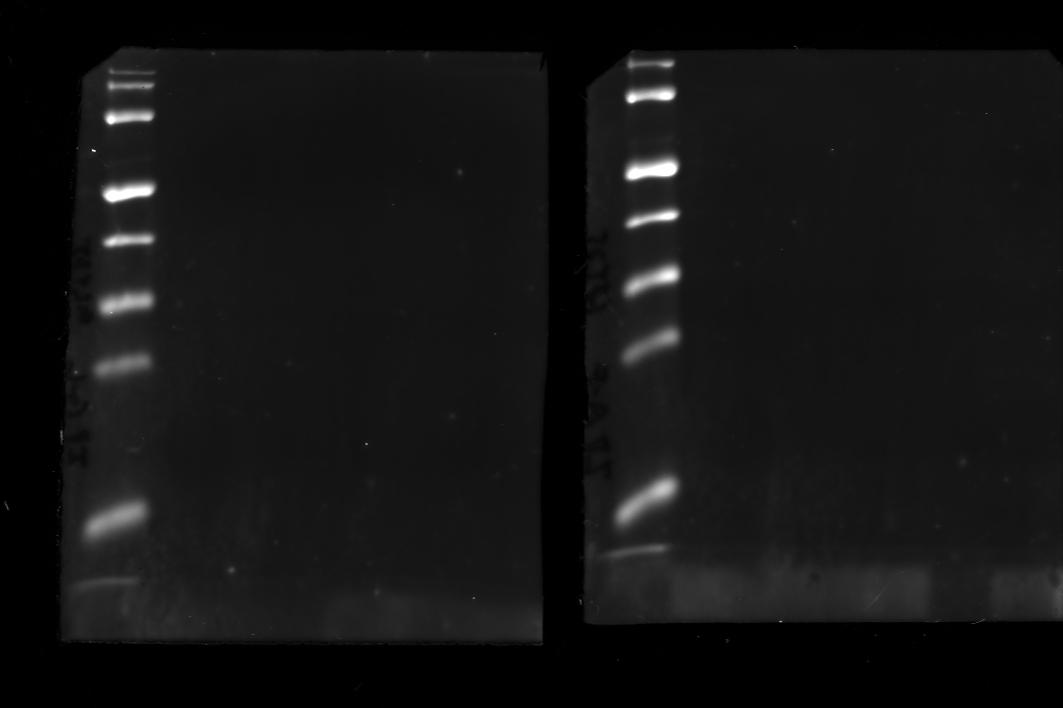

Supplement: Figure 5—figure supplement 1—source data 1. [file elife-68958-fig5-figsupp1-data1.zip › Figure 5-figure supplement 1ΓÇôsource data 1/Figure 5-figure supplement 1 full raw unedited blots files/original_files for C/2021-02-24-142408/700.TIF]

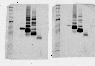

Supplement: Figure 5—figure supplement 1—source data 1. [file elife-68958-fig5-figsupp1-data1.zip › Figure 5-figure supplement 1ΓÇôsource data 1/Figure 5-figure supplement 1 full raw unedited blots files/original_files for C/2021-02-24-142408/2021-02-24-142408_a-GST MDA5 GST-TRIM25 3_TH.jpg]

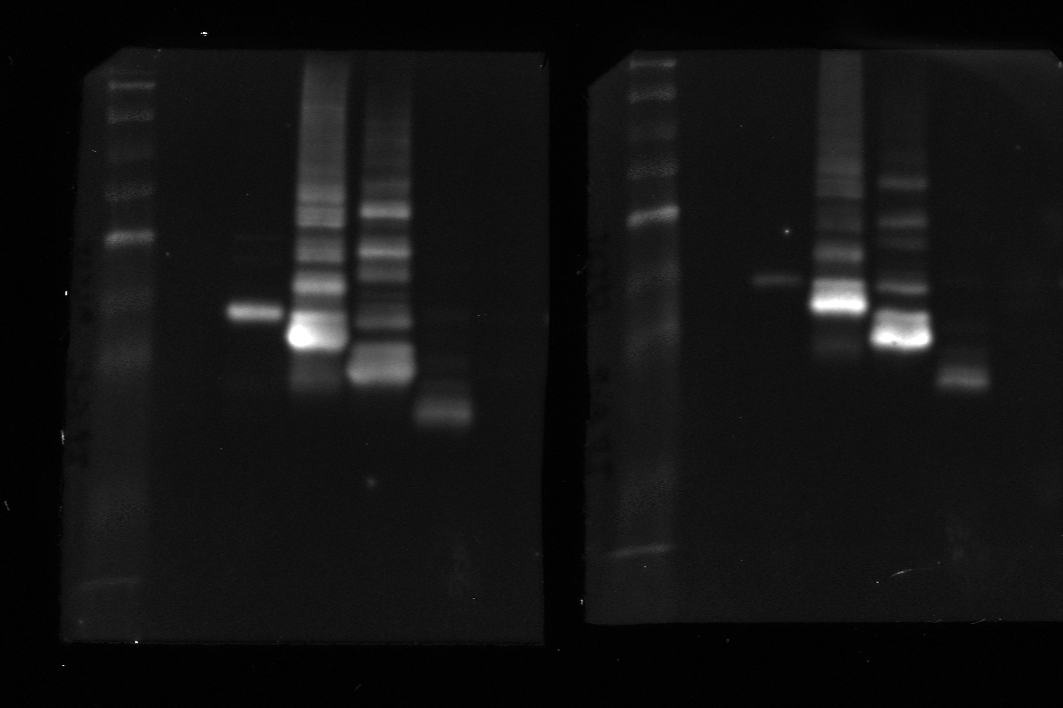

Supplement: Figure 5—figure supplement 1—source data 1. [file elife-68958-fig5-figsupp1-data1.zip › Figure 5-figure supplement 1ΓÇôsource data 1/Figure 5-figure supplement 1 full raw unedited blots files/original_files for C/2021-02-24-142408/800.TIF]

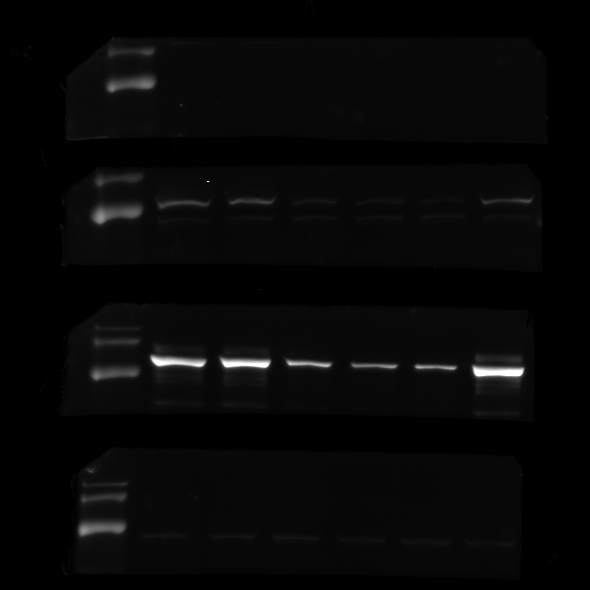

Supplement: Figure 5—figure supplement 1—source data 1. [file elife-68958-fig5-figsupp1-data1.zip › Figure 5-figure supplement 1ΓÇôsource data 1/Figure 5-figure supplement 1 full raw unedited blots files/original_files for C/2021-02-28-185948/700.TIF]

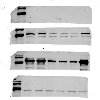

Supplement: Figure 5—figure supplement 1—source data 1. [file elife-68958-fig5-figsupp1-data1.zip › Figure 5-figure supplement 1ΓÇôsource data 1/Figure 5-figure supplement 1 full raw unedited blots files/original_files for C/2021-02-28-185948/2021-02-28-185948_a-GST TRIM25_TH.jpg]

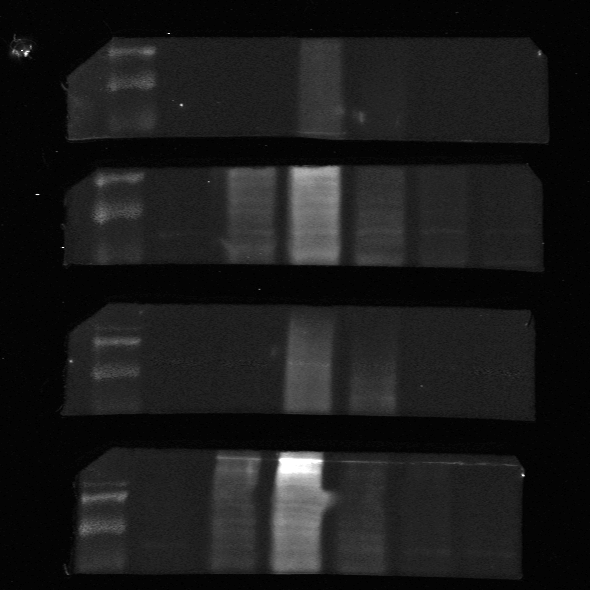

Supplement: Figure 5—figure supplement 1—source data 1. [file elife-68958-fig5-figsupp1-data1.zip › Figure 5-figure supplement 1ΓÇôsource data 1/Figure 5-figure supplement 1 full raw unedited blots files/original_files for C/2021-02-28-185948/800.TIF]

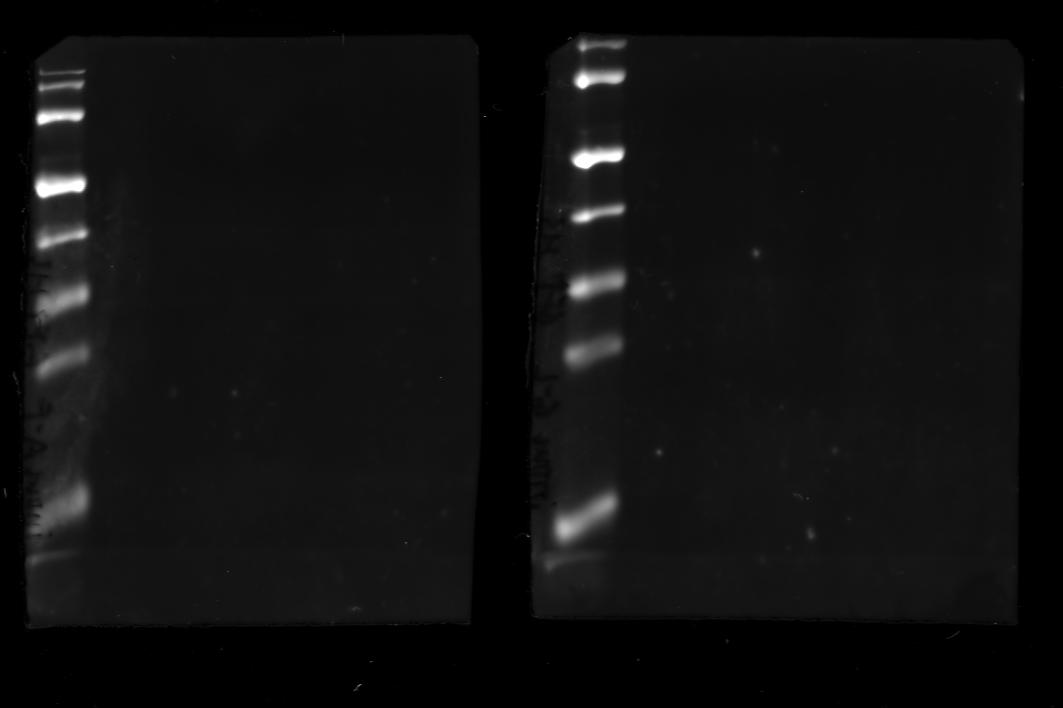

Supplement: Figure 5—figure supplement 1—source data 1. [file elife-68958-fig5-figsupp1-data1.zip › Figure 5-figure supplement 1ΓÇôsource data 1/Figure 5-figure supplement 1 full raw unedited blots files/original_files for C/2021-02-24-143343/700.TIF]

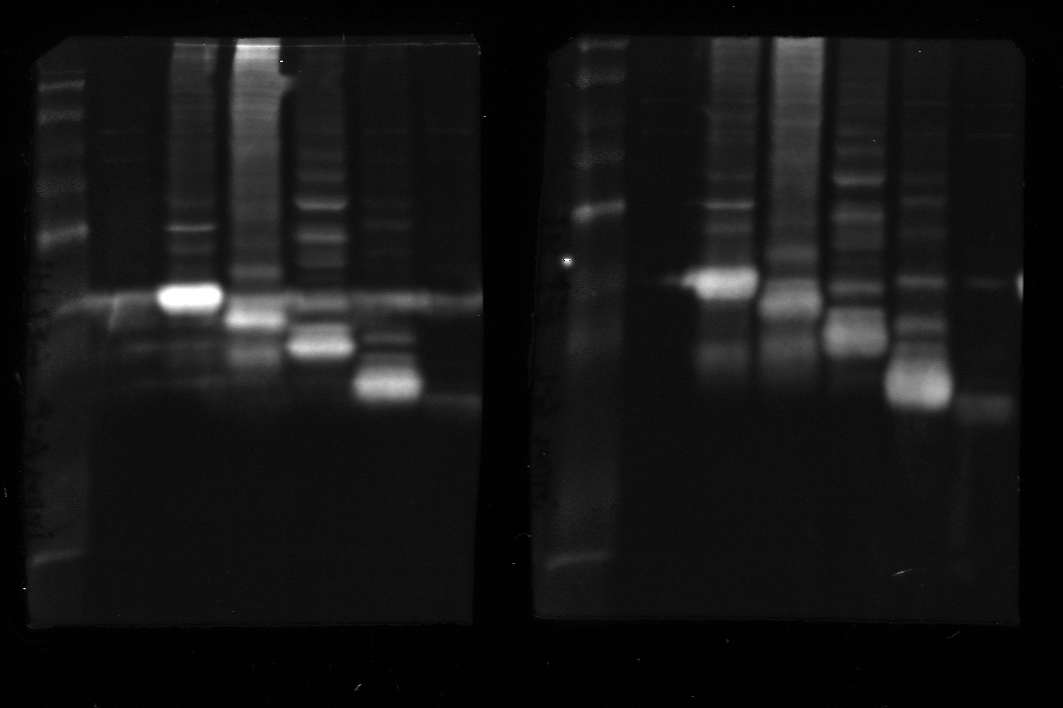

Supplement: Figure 5—figure supplement 1—source data 1. [file elife-68958-fig5-figsupp1-data1.zip › Figure 5-figure supplement 1ΓÇôsource data 1/Figure 5-figure supplement 1 full raw unedited blots files/original_files for C/2021-02-24-143343/800.TIF]

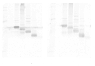

Supplement: Figure 5—figure supplement 1—source data 1. [file elife-68958-fig5-figsupp1-data1.zip › Figure 5-figure supplement 1ΓÇôsource data 1/Figure 5-figure supplement 1 full raw unedited blots files/original_files for C/2021-02-24-143343/2021-02-24-143343_a-GST MDA5 GST-TRIM25 4_TH.jpg]

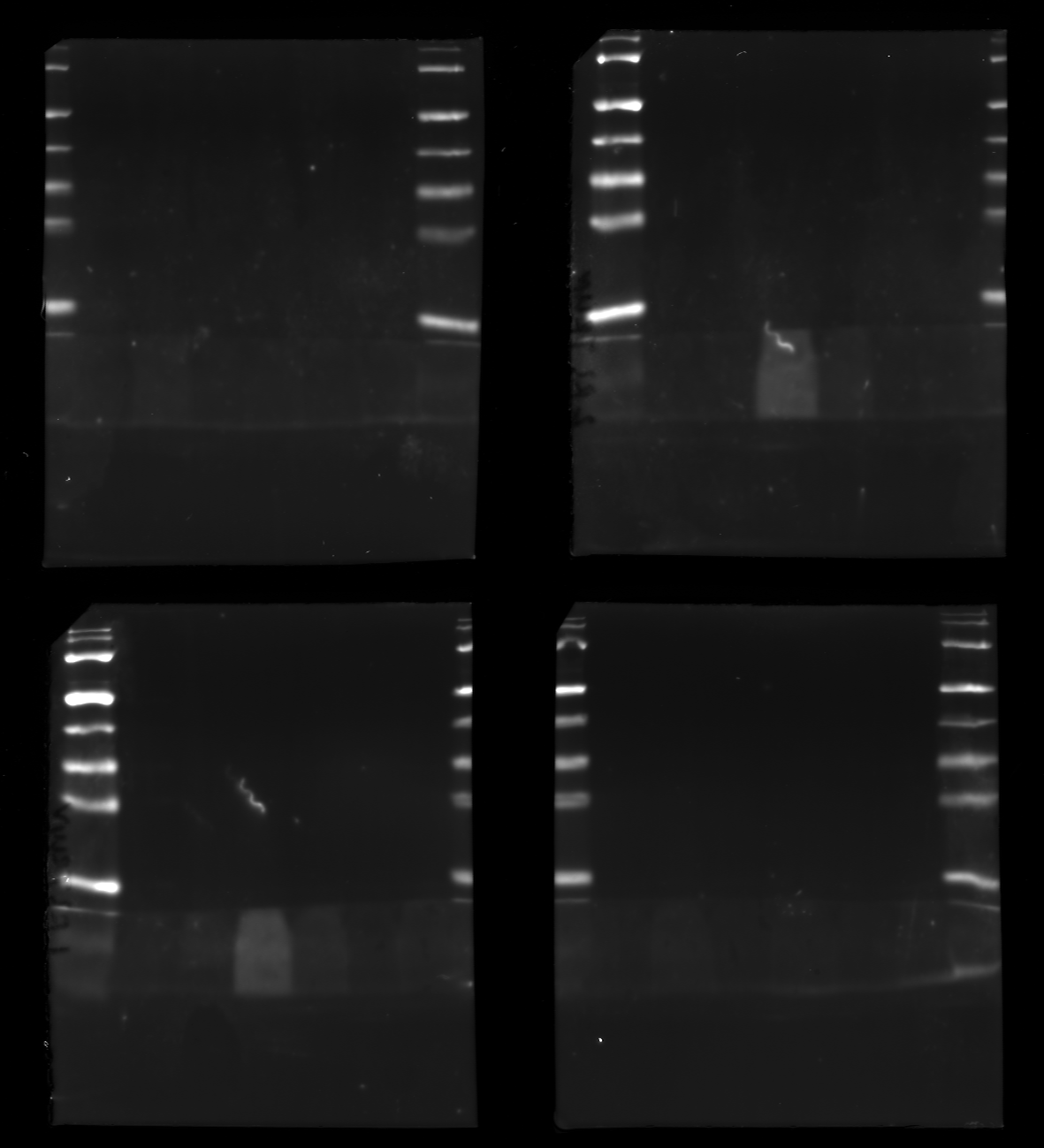

Supplement: Figure 5—figure supplement 1—source data 1. [file elife-68958-fig5-figsupp1-data1.zip › Figure 5-figure supplement 1ΓÇôsource data 1/Figure 5-figure supplement 1 full raw unedited blots files/original_files for B/2021-03-14-190820/700.TIF]

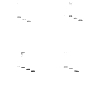

Supplement: Figure 5—figure supplement 1—source data 1. [file elife-68958-fig5-figsupp1-data1.zip › Figure 5-figure supplement 1ΓÇôsource data 1/Figure 5-figure supplement 1 full raw unedited blots files/original_files for B/2021-03-14-190820/2021-03-14-190820_a-RT3 TRUN_TH.jpg]

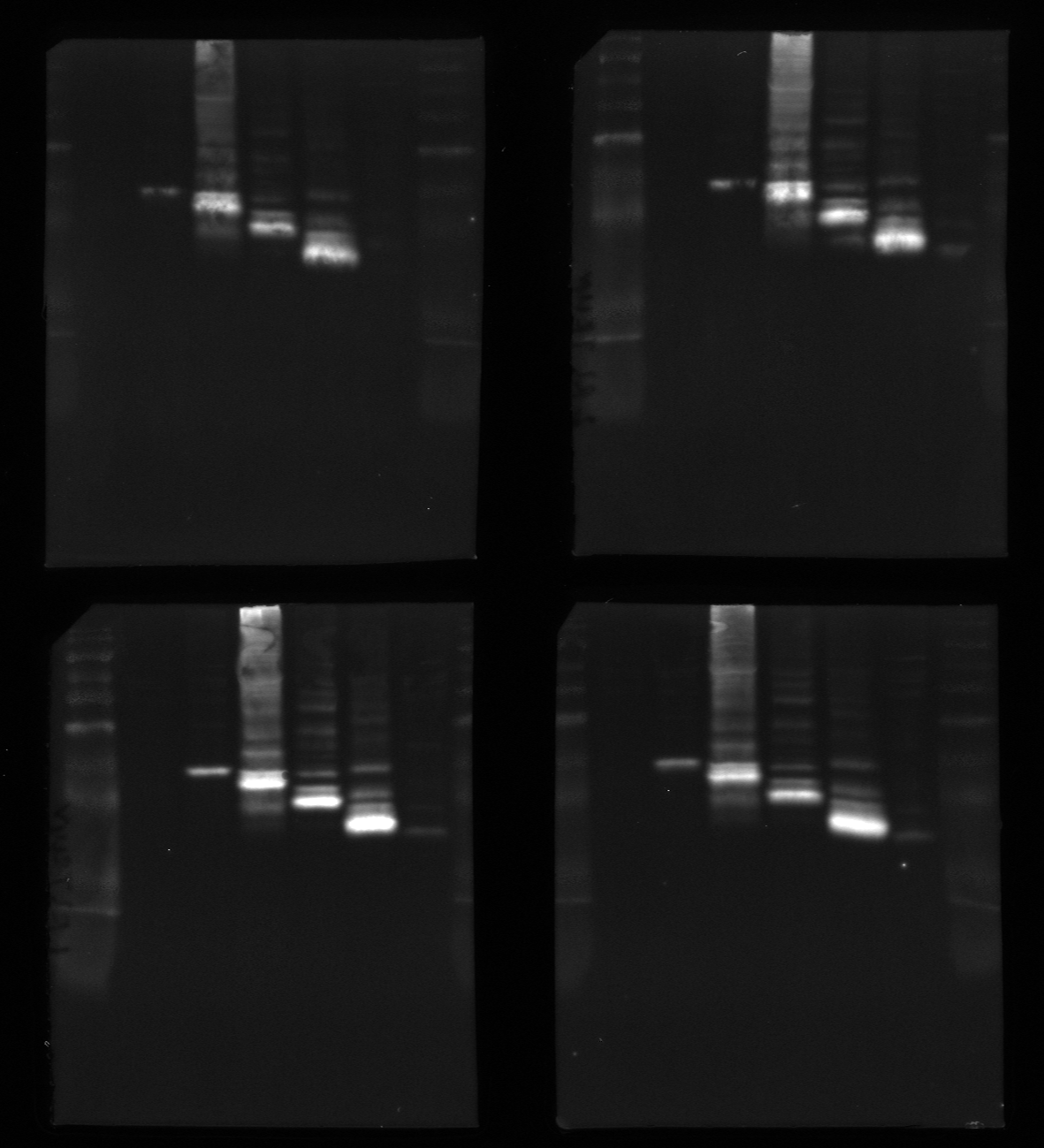

Supplement: Figure 5—figure supplement 1—source data 1. [file elife-68958-fig5-figsupp1-data1.zip › Figure 5-figure supplement 1ΓÇôsource data 1/Figure 5-figure supplement 1 full raw unedited blots files/original_files for B/2021-03-14-190820/800.TIF]

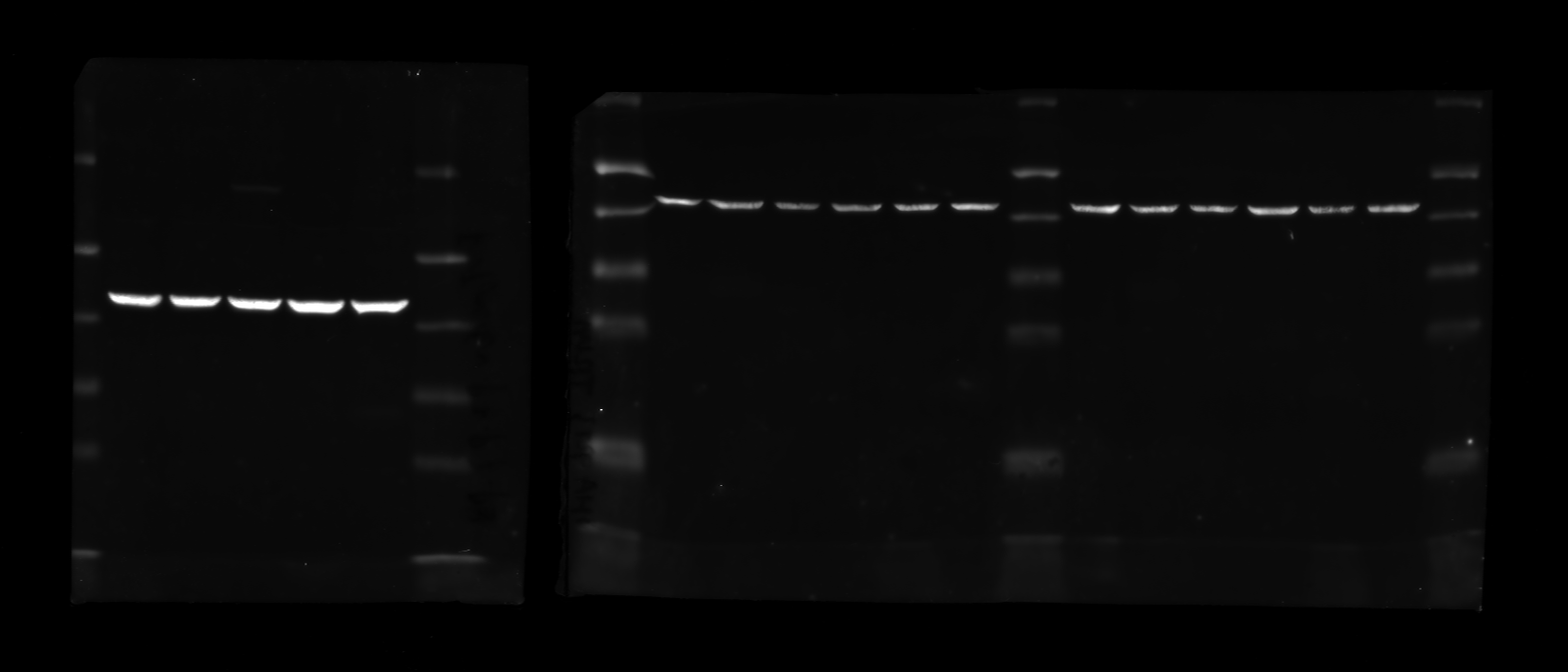

Supplement: Figure 5—figure supplement 1—source data 1. [file elife-68958-fig5-figsupp1-data1.zip › Figure 5-figure supplement 1ΓÇôsource data 1/Figure 5-figure supplement 1 full raw unedited blots files/original_files for B/2021-03-04-212108/700.TIF]

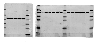

Supplement: Figure 5—figure supplement 1—source data 1. [file elife-68958-fig5-figsupp1-data1.zip › Figure 5-figure supplement 1ΓÇôsource data 1/Figure 5-figure supplement 1 full raw unedited blots files/original_files for B/2021-03-04-212108/2021-03-04-212108_a-Actin_TH.jpg]

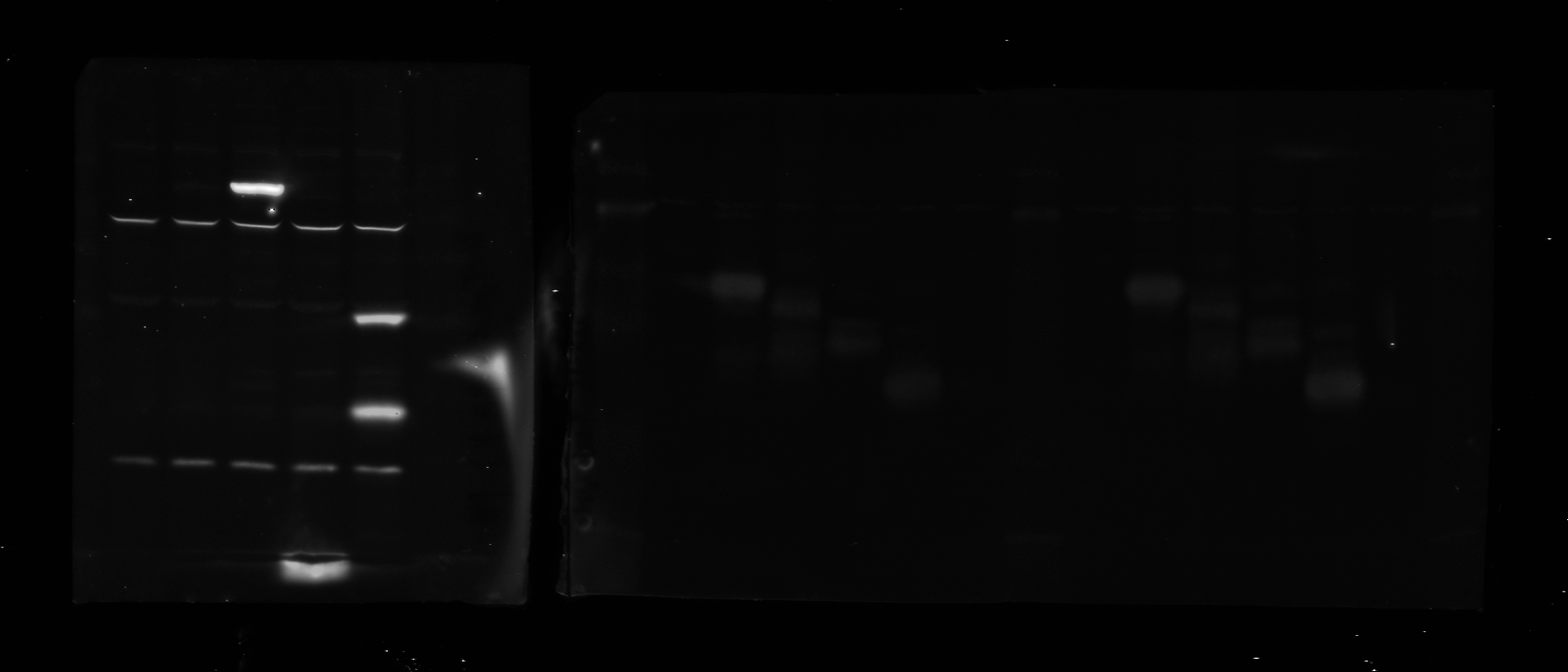

Supplement: Figure 5—figure supplement 1—source data 1. [file elife-68958-fig5-figsupp1-data1.zip › Figure 5-figure supplement 1ΓÇôsource data 1/Figure 5-figure supplement 1 full raw unedited blots files/original_files for B/2021-03-04-212108/800.TIF]

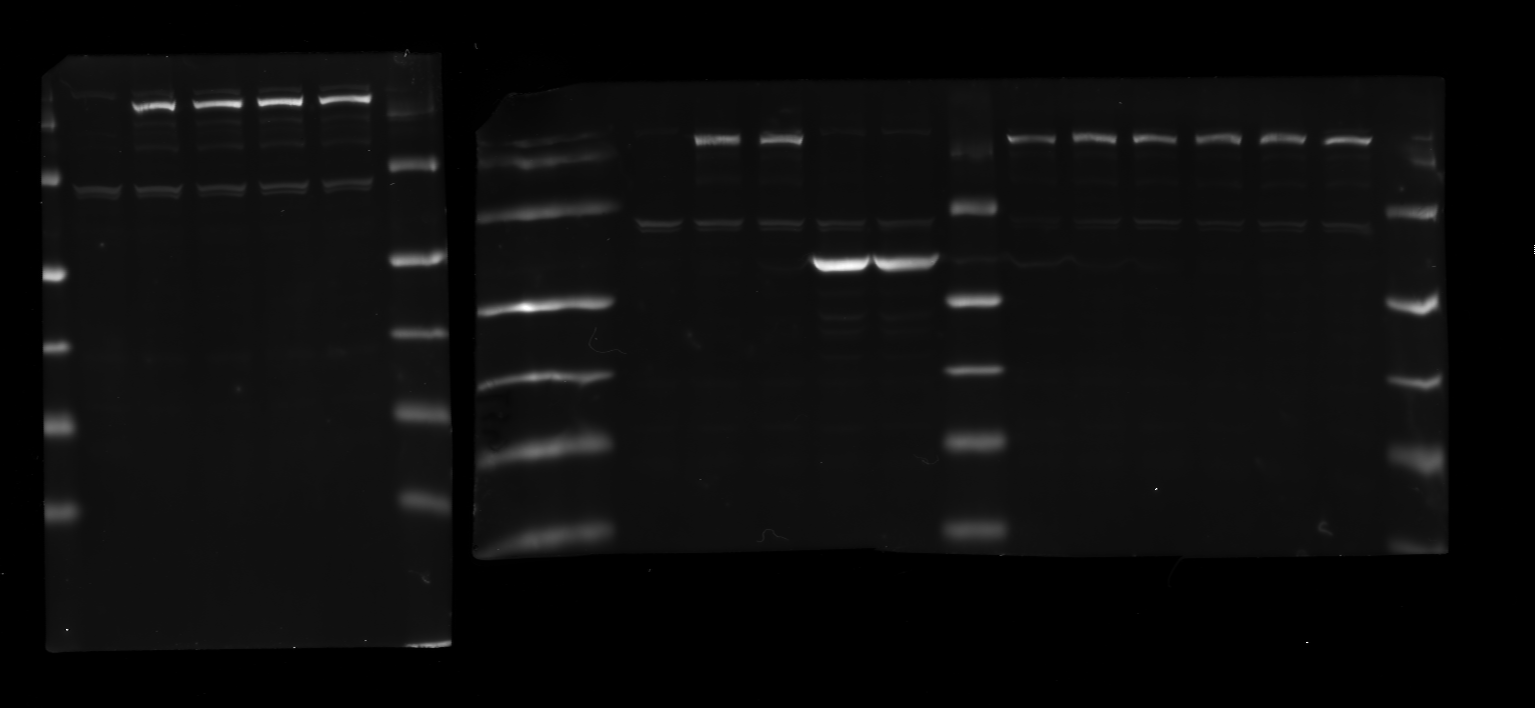

Supplement: Figure 5—figure supplement 1—source data 1. [file elife-68958-fig5-figsupp1-data1.zip › Figure 5-figure supplement 1ΓÇôsource data 1/Figure 5-figure supplement 1 full raw unedited blots files/original_files for B/2021-02-08-143629/700.TIF]

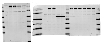

Supplement: Figure 5—figure supplement 1—source data 1. [file elife-68958-fig5-figsupp1-data1.zip › Figure 5-figure supplement 1ΓÇôsource data 1/Figure 5-figure supplement 1 full raw unedited blots files/original_files for B/2021-02-08-143629/2021-02-08-143629_a-GST MDA5 AND MDA5 CARD_TH.jpg]

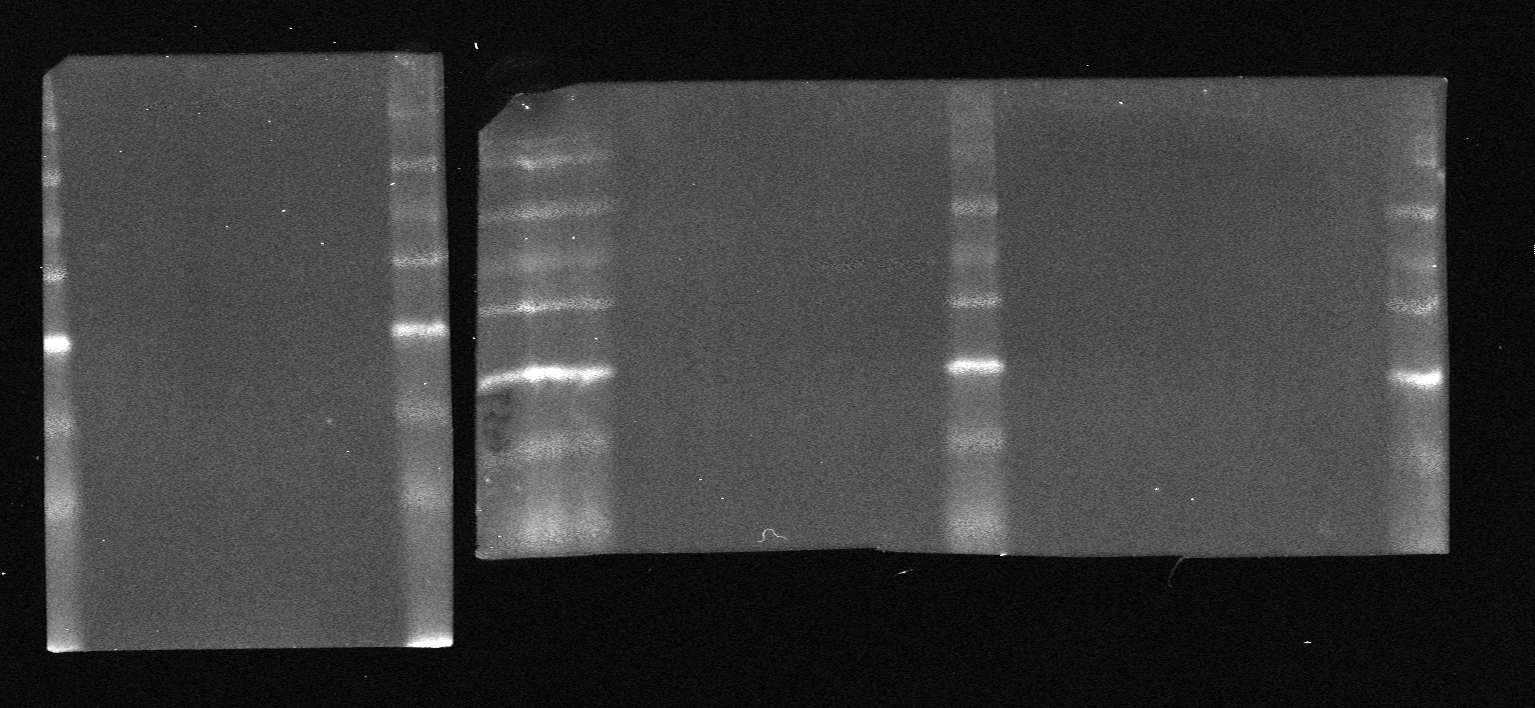

Supplement: Figure 5—figure supplement 1—source data 1. [file elife-68958-fig5-figsupp1-data1.zip › Figure 5-figure supplement 1ΓÇôsource data 1/Figure 5-figure supplement 1 full raw unedited blots files/original_files for B/2021-02-08-143629/800.TIF]

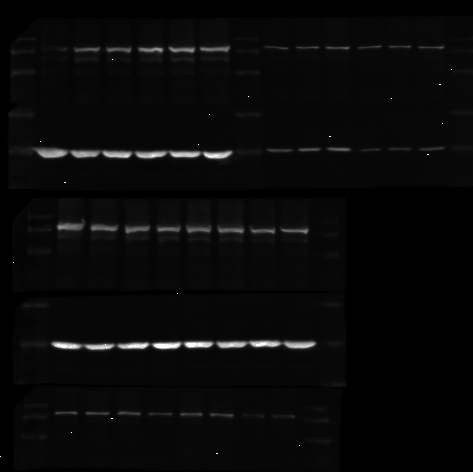

Supplement: Figure 5—figure supplement 1—source data 1. [file elife-68958-fig5-figsupp1-data1.zip › Figure 5-figure supplement 1ΓÇôsource data 1/Figure 5-figure supplement 1 full raw unedited blots files/original_files for A/2020-07-04-224236/700.TIF]

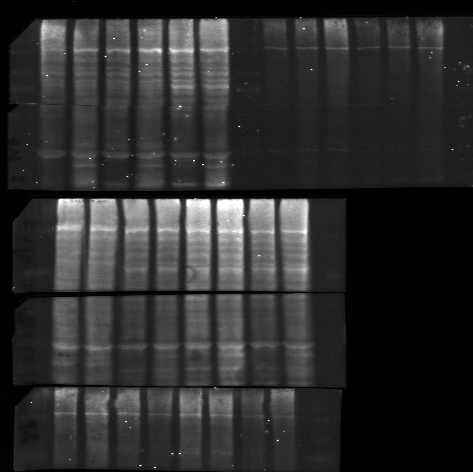

Supplement: Figure 5—figure supplement 1—source data 1. [file elife-68958-fig5-figsupp1-data1.zip › Figure 5-figure supplement 1ΓÇôsource data 1/Figure 5-figure supplement 1 full raw unedited blots files/original_files for A/2020-07-04-224236/800.TIF]

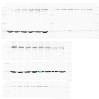

Supplement: Figure 5—figure supplement 1—source data 1. [file elife-68958-fig5-figsupp1-data1.zip › Figure 5-figure supplement 1ΓÇôsource data 1/Figure 5-figure supplement 1 full raw unedited blots files/original_files for A/2020-07-04-224236/2020-07-04-224236_a-GFP RIG I Actin_TH.jpg]

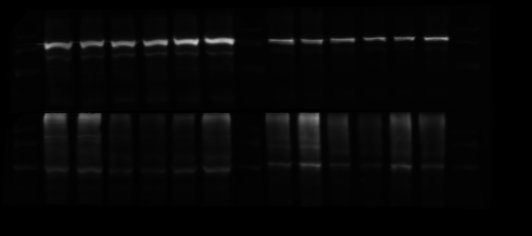

Supplement: Figure 5—figure supplement 1—source data 1. [file elife-68958-fig5-figsupp1-data1.zip › Figure 5-figure supplement 1ΓÇôsource data 1/Figure 5-figure supplement 1 full raw unedited blots files/original_files for A/2020-06-21-142240/700.TIF]

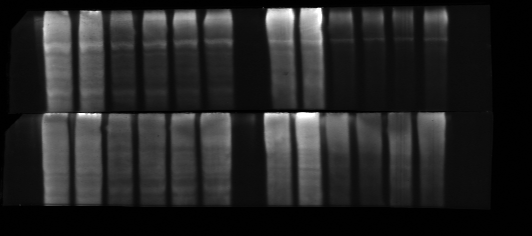

Supplement: Figure 5—figure supplement 1—source data 1. [file elife-68958-fig5-figsupp1-data1.zip › Figure 5-figure supplement 1ΓÇôsource data 1/Figure 5-figure supplement 1 full raw unedited blots files/original_files for A/2020-06-21-142240/800.TIF]

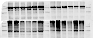

Supplement: Figure 5—figure supplement 1—source data 1. [file elife-68958-fig5-figsupp1-data1.zip › Figure 5-figure supplement 1ΓÇôsource data 1/Figure 5-figure supplement 1 full raw unedited blots files/original_files for A/2020-06-21-142240/2020-06-21-142240_a-GFP RIGI GFP T25_TH.jpg]

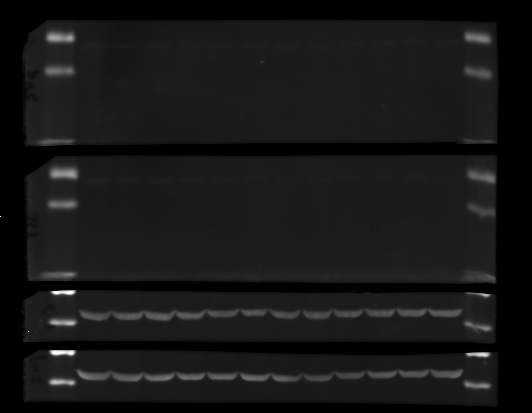

Supplement: Figure 6—source data 1. [file elife-68958-fig6-data1.zip › Figure 6ΓÇôsource data 1/Figure 6 full raw unedited blots files/original_files for D/2020-09-15-104145/700.TIF]

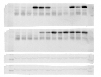

Supplement: Figure 6—source data 1. [file elife-68958-fig6-data1.zip › Figure 6ΓÇôsource data 1/Figure 6 full raw unedited blots files/original_files for D/2020-09-15-104145/2020-09-15-104145_a-HA Rt3 endo Rt3 Actin_TH.jpg]

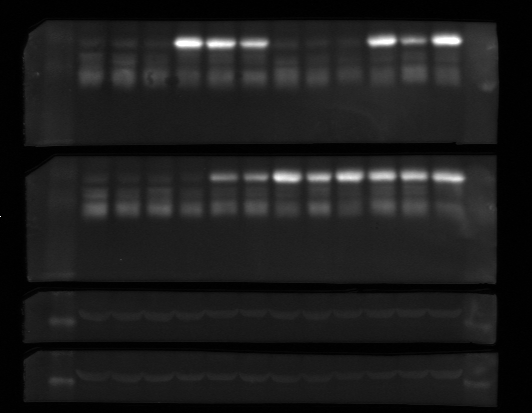

Supplement: Figure 6—source data 1. [file elife-68958-fig6-data1.zip › Figure 6ΓÇôsource data 1/Figure 6 full raw unedited blots files/original_files for D/2020-09-15-104145/800.TIF]

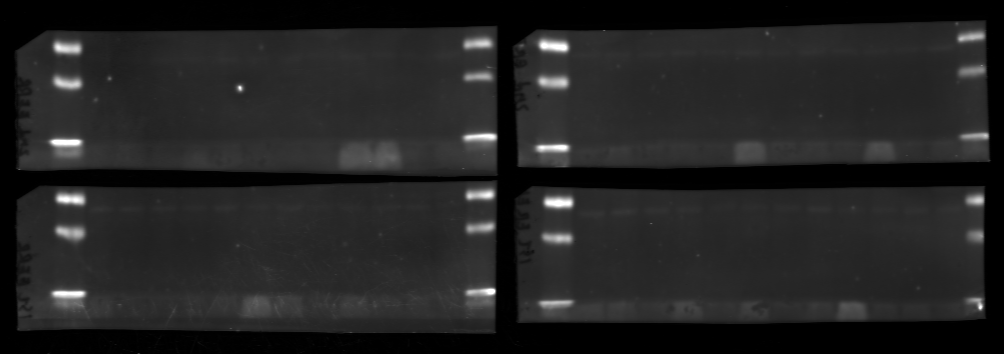

Supplement: Figure 6—source data 1. [file elife-68958-fig6-data1.zip › Figure 6ΓÇôsource data 1/Figure 6 full raw unedited blots files/original_files for D/2020-09-14-110707/700.TIF]

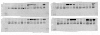

Supplement: Figure 6—source data 1. [file elife-68958-fig6-data1.zip › Figure 6ΓÇôsource data 1/Figure 6 full raw unedited blots files/original_files for D/2020-09-14-110707/2020-09-14-110707_a-HA-ev HA-Rt3_TH.jpg]

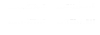

Supplement: Figure 6—source data 1. [file elife-68958-fig6-data1.zip › Figure 6ΓÇôsource data 1/Figure 6 full raw unedited blots files/original_files for D/2020-09-14-110707/2020-09-14-110707_a-HA-Rt3 DMEM VSV_TH.jpg]

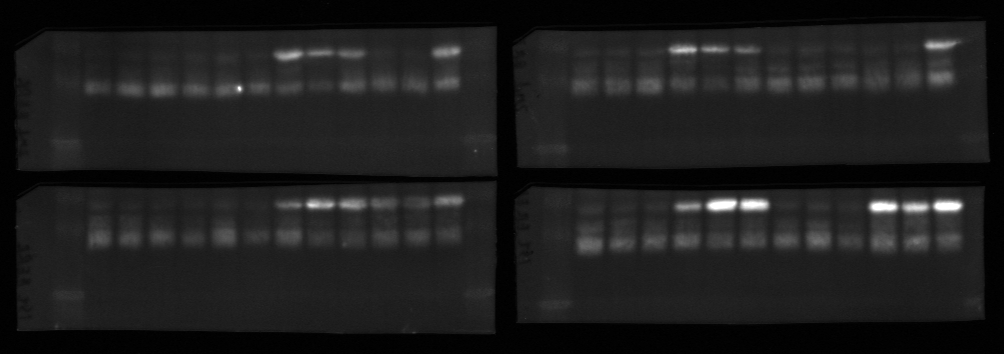

Supplement: Figure 6—source data 1. [file elife-68958-fig6-data1.zip › Figure 6ΓÇôsource data 1/Figure 6 full raw unedited blots files/original_files for D/2020-09-14-110707/800.TIF]

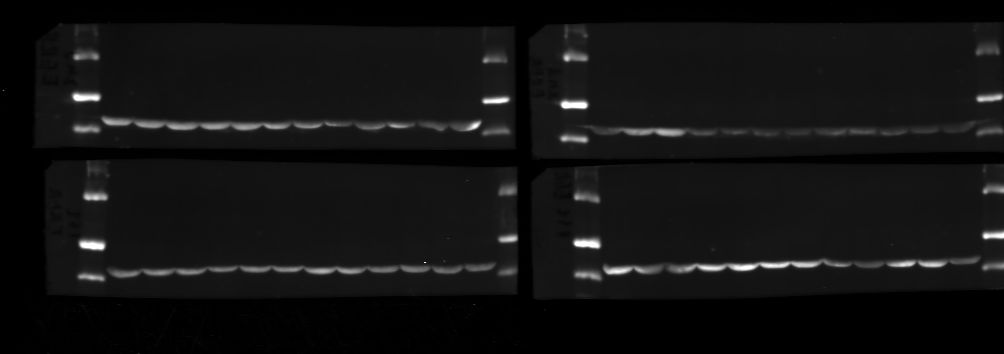

Supplement: Figure 6—source data 1. [file elife-68958-fig6-data1.zip › Figure 6ΓÇôsource data 1/Figure 6 full raw unedited blots files/original_files for D/2020-09-14-111915/700.TIF]

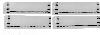

Supplement: Figure 6—source data 1. [file elife-68958-fig6-data1.zip › Figure 6ΓÇôsource data 1/Figure 6 full raw unedited blots files/original_files for D/2020-09-14-111915/2020-09-14-111915_a-Actin_TH.jpg]

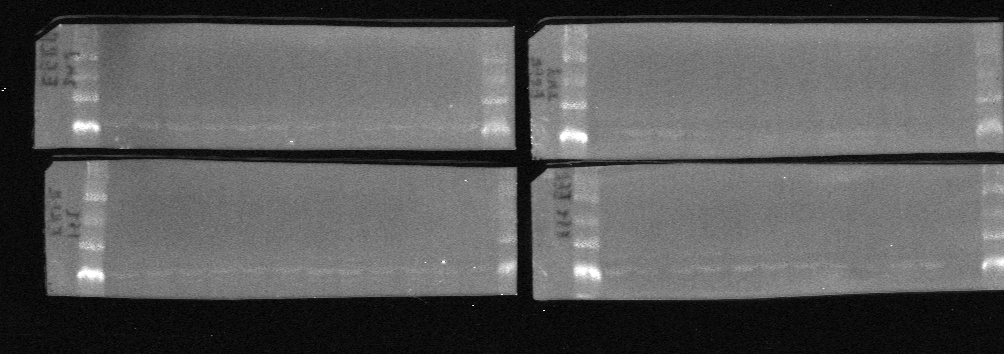

Supplement: Figure 6—source data 1. [file elife-68958-fig6-data1.zip › Figure 6ΓÇôsource data 1/Figure 6 full raw unedited blots files/original_files for D/2020-09-14-111915/800.TIF]

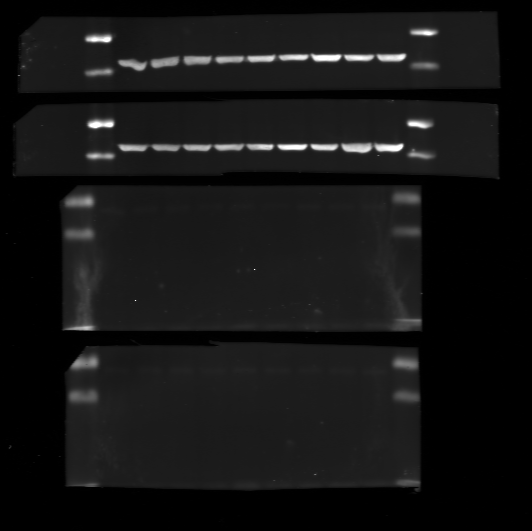

Supplement: Figure 6—source data 1. [file elife-68958-fig6-data1.zip › Figure 6ΓÇôsource data 1/Figure 6 full raw unedited blots files/original_files for B/2020-09-17-141511/700.TIF]

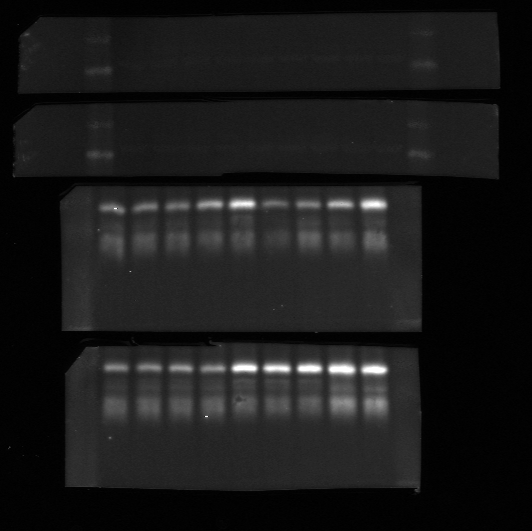

Supplement: Figure 6—source data 1. [file elife-68958-fig6-data1.zip › Figure 6ΓÇôsource data 1/Figure 6 full raw unedited blots files/original_files for B/2020-09-17-141511/800.TIF]

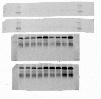

Supplement: Figure 6—source data 1. [file elife-68958-fig6-data1.zip › Figure 6ΓÇôsource data 1/Figure 6 full raw unedited blots files/original_files for B/2020-09-17-141511/2020-09-17-141511_a-Actin a-Rtn3_TH.jpg]
